# Towards the Preparation of Stable Cyclic Amino(ylide)Carbenes

**DOI:** 10.3390/molecules25040796

**Published:** 2020-02-12

**Authors:** Henning Steinert, Christopher Schwarz, Alexander Kroll, Viktoria H. Gessner

**Affiliations:** Faculty of Chemistry and Biochemistry, Chair of Inorganic Chemistry II, Ruhr University Bochum, Universitätsstr. 150, 44801 Bochum, Germany; henning.steinert@rub.de (H.S.); christopher.m.schwarz@rub.de (C.S.); Alexander.kroll@rub.de (A.K.)

**Keywords:** carbenes, ylides, DFT calculations, electronic structure, catalysis, ligands, structure–activity relationship

## Abstract

Cyclic amino(ylide)carbenes (CAYCs) are the ylide-substituted analogues of *N*-heterocyclic Carbenes (NHCs). Due to the stronger π donation of the ylide compared to an amino moiety they are stronger donors and thus are desirable ligands for catalysis. However, no stable CAYC has been reported until today. Here, we describe experimental and computational studies on the synthesis and stability of CAYCs based on pyrroles with trialkyl onium groups. Attempts to isolate two CAYCs with trialkyl phosphonium and sulfonium ylides resulted in the deprotonation of the alkyl groups instead of the formation of the desired CAYCs. In case of the PCy_3_-substituted system, the corresponding ylide was isolated, while deprotonation of the SMe_2_-functionalized compound led to the formation of ethene and the thioether. Detailed computational studies on various trialkyl onium groups showed that both the α- and β-deprotonated compounds were energetically favored over the free carbene. The most stable candidates were revealed to be α-hydrogen-free adamantyl-substituted onium groups, for which β-deprotonation is less favorable at the bridgehead position. Overall, the calculations showed that the isolation of CAYCs should be possible, but careful design is required to exclude decomposition pathways such as deprotonations at the onium group.

## 1. Introduction

**Figure 1:**
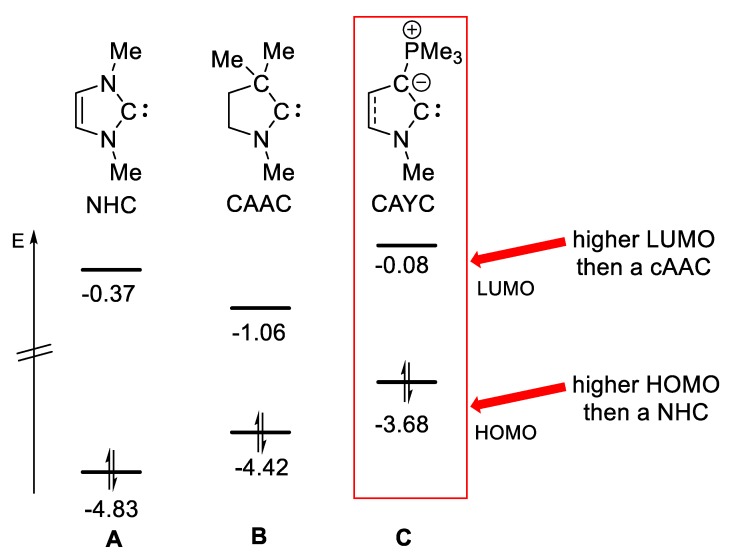
Schematic representation of the HOMO and LUMO properties of different singlet carbenes. Eigenvalues of the frontier orbitals were taken from Reference [21].

Since the isolation of the first stable singlet carbenes by Bertrand and Arduengo [1] 30 years ago, these divalent carbon ligands have led to a revolution in coordination chemistry and produced most remarkable developments in various fields of chemistry. Owing to their high donor strength, they are applied as potent ligands in transition metal catalysis [2,3,4,5] (e.g., in the second generation Grubbs catalyst [6]), in organo catalysis [2,7,8,9,10], or main group chemistry [2,11,12], such as for the stabilization of reactive [2,12], electron-deficient [2,12], or low-valent species [2,12]. The success of carbenes is mostly based on their thermal stability and high donor capacity, which usually surpasses that of phosphines or amines. Furthermore, detailed studies by various research groups over the world have demonstrated the straightforward tunability of the electronic structure, particularly the HOMO–LUMO gap, thus allowing for the adjustment of the donor and acceptor properties for the desired application. While the first years of the “carbene revolution” were defined by the Arduengo-type *N*-heterocyclic carbenes (NHC) **A** (Figure 1), the past years have seen a huge expansion of the carbene portfolio by variation of the substituents [13,14,15,16,17,18] or the geometry (cyclic versus acyclic) [18] at the carbenic carbon atom. This has led to drastic changes in the carbene properties and hence to a myriad of new fields of applications. For example, in a landmark report, Bertrand and coworkers demonstrated that the replacement of one nitrogen substituent in an NHC by a sp^3^-carbon substituent to form acyclic and cyclic alkyl(amino)carbenes (CAAC, **B**) significantly increases the donor and acceptor properties of these compounds [18]. This results in a sufficiently small HOMO–LUMO (highest molecular orbital, lowest molecular orbital, respectively) gap to allow for straightforward dihydrogen activation [19].

In recent years, many different types of carbenes with different donor/acceptor properties have been reported, many of which, however, suffer from insufficient stability for broad applications. One particularly interesting class of singlet carbenes is the amino(ylide)carbenes (CAYCs, **C**). Here, one of the nitrogen substituents of an NHC was replaced by an ylide group. Due to the lower electronegativity of carbon compared to nitrogen, such an ylide-substitution should result in increased HOMO and LUMO levels, thus making CAYCs particularly strong donors with interesting electronic properties [20,21,22,23,24].

**Figure 2:**
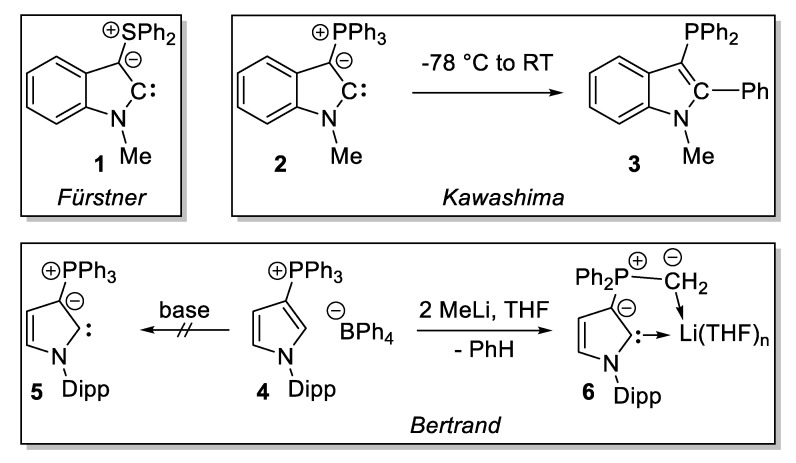
Attempts to isolate CAYCs (Dipp = 2,6-diisopropylphenyl) [25,26,27].

Initial attempts to isolate CAYCs were reported in 2008. Kawashima and Fürstner targeted the isolation of **1** and **2**, respectively (Figure 2) [25,26]. However, both compounds were found to decompose at low temperatures, and no room temperature stable CAYC has been reported until today. Nonetheless, the successful synthesis of the CAYCs could be unambiguously confirmed by trapping the carbenes in the coordination sphere of a metal. Isolation of an anionic CAYC was also reported by Bertand and coworkers. They likewise failed in the isolation of the CAYC **5** by deprotonation of phosphonium salt **4** with different bases (Figure 2). However, employment of two equiv. of methyllithium resulted in the formation of the anionic CAYC **6** via P-C cleavage and elimination of one of the phosphorus-bound phenyl groups [27]. In contrast to carbenes, a couple of ylide-substituted heavier tetrylenes have been isolated in recent years due to the generally higher stability of the heavier carbenes resulting from the increased stability of the +2 oxidation state as a consequence of the larger s,p orbital separation [28,29,30]. They impressively demonstrated unique reactivities owing to their high donor capacity imparted by the ylide-substituents.

During their attempts to isolate a CAYC, Kawashima and coworkers uncovered a decomposition reaction pathway of **2** which led to the formation of indole **3** via transfer of an aryl substituent of the onium group to the carbene center [25]. Due to the lower bond strength of a P-C_sp2_ and S-C_sp2_, respectively, compared to bonds with a sp^3^ hybridized carbon atom, such a transfer should be less likely for trialkyl substituted onium moieties. With this in mind, we set out to synthesize CAYCs **7** and **8** with a dialkylsulfonium and trialkylphosphonium group.

## 2. Results

### 2.1. Attempted Synthesis of CAYCs with Trialklyl Onium Groups

**Scheme 1:**
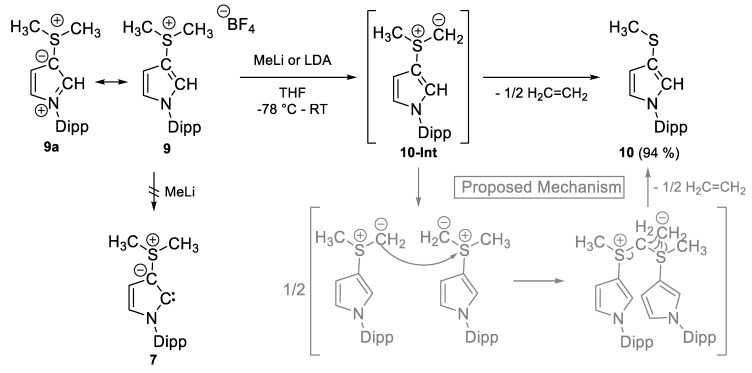
Deprotonation of the sulfonium salt **9** to form thioether **10** and proposed mechanism.

**Figure 3:**
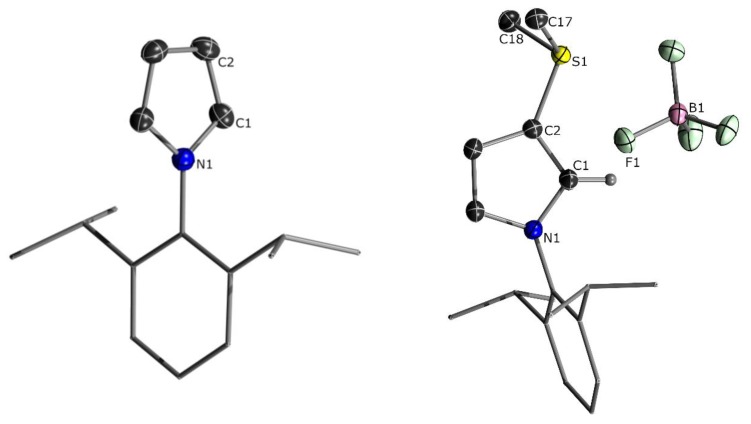
Molecular structure of *N*-(2,6-diisopropylphenyl)pyrrole (left) and **9**. Ellipsoids are drawn at the 50% probability level. Selected bond lengths (Å) and angles (°): N-(1,2-Diisopropylphenyl)pyrrole: N1–C1 1.374(2), N1–C4 1.375(2), C1–C2 1.370(2), C2–C3 1.416(3), C3–C4 1.367(2). **9**: S2–C2 1.748(1), N1–C1 1.362(1), C1–C2 1.376(2), C2–C3 1.412(2).

We started our investigations with the attempt to synthesize the dimethylsulfonium carbene **7**. The sulfonium salt **9** was synthesized according to a literature procedure [26,31,32] via treatment of *N*-(2,6-diisopropylphenyl)pyrrole [33] with DMSO and trifluoroacetic anhydride, and was isolated in 58% yield as colorless solid. The salt **9** exhibited a pseudo triplet in the ^1^H NMR spectrum at 7.35 ppm for the NCHCSMe_2_ moiety. Recently, Szostak and coworkers showed that the σ-donor strengths of carbenes can easily be estimated by measurement of the ^1^*J*_CH_ coupling constant in the protonated carbene precursors [34]. Measurement of the ^1^*J*_CH_ coupling constant of **9** revealed a value of 194.0 Hz, which suggests that the CAYC **7** is a stronger donor than typical NHCs and of a strength comparable to CAACs. The structure of **9** was unambiguously confirmed by single-crystal X-ray diffraction analysis (Figure 3). Deprotonation was next attempted at low temperatures. Addition of methyllithium (MeLi) or lithium diisopropylamide (LDA) at –78 °C to a solution of **9** in THF led to a color change from colorless to brown and the selective formation of a single new compound, as assessed by NMR spectroscopy (Scheme 1). Isolation revealed the product to be thioether **10**, which could be isolated as colorless oil in 94% yield. Compound **5** is characterized by a signal at 6.69 ppm in the ^1^H NMR spectrum for the imidazolium hydrogen atom and at 2.22 ppm for the sulfur-bound methyl group. The formation of **10** clearly resulted from the deprotonation of the sulfonium salt at the methyl group and the intermediate generation of yilde **10-Int**. This ylide intermediate presumably reacted in a kind of Corey–Chaykovsky reaction [35,36] with itself to form **8** under concomitant formation of ethene. Indeed, NMR monitoring of the formation of **10** clearly showed the formed ethene by appearance of a signal at 5.35 ppm in the ^1^H NMR spectrum.

Comparison of the structure of the sulfonium salt **9** with that of the pyrrole precursor *N*-(2,6-diisopropylphenyl)pyrrole revealed similar structural changes upon introduction of the onium moiety, as previously observed by Fürstner and coworkers. [26] As a result of the delocalization of the charge of the sulfonium moiety into the pyrrole ring of **9** and the thus more pronounced contribution of the ylidic resonance structure **9b** to the overall electronic structure of **9**, the C1–C2 bond in **9** (1.376(2) Å) was elongated compared the one found in *N*-(2,6-diisopropylphenyl)pyrrole (1.370(3) and 1.367(2) Å). Likewise, the C1–N1 bond shortened from approx. 1.374 Å in the unsubstituted pyrrole to 1.362(1) Å in **9**.

**Scheme 2:**
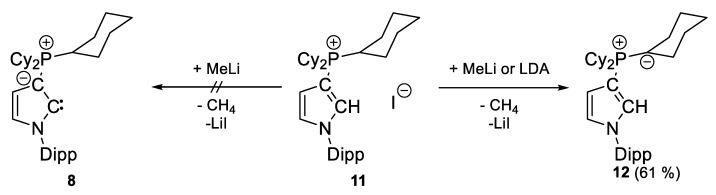
Deprotonation of the phosphonium salt **11** to form **12.**

**Figure 4:**
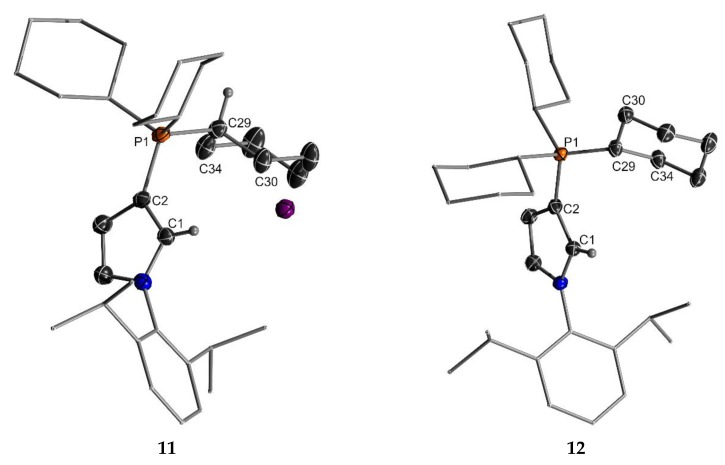
Molecular structures of (left) **11** and (right) **12**. Ellipsoids are drawn at the 50% probability level. Selected bond lengths are provided in Table 1.

Since deprotonation of **9** at the methyl group was clearly kinetically favored over the desired carbene formation, we next turned our attention towards a tricyclohexyl phosphonium salt. We envisioned that the cyclohexyl group should be less prone to ylide formation (due to electronic and steric reasons) and thus allow for the formation of carbene **8**. The phosphonium precursor **11** was synthesized by treatment of the corresponding iodo compound, obtained similarly to a known procedure [37] with tricyclohexylphosphine in the presence of 5 mol% Ni(cod)_2_ (cod = 1,5-cyclooctadiene) at elevated temperatures and isolated as colorless solid in 61% yield. Compound **11** was characterized by multi-nuclear NMR spectroscopy, elemental, and single-crystal X-ray diffraction analysis (Figure 4). The phosphonium salt exhibited a signal at 26.1 ppm in the ^31^P NMR spectrum and a multiplet at 7.23–7.26 ppm in the ^1^H NMR spectrum for the pyrrole hydrogen atom. The ^1^*J*_CH_ coupling constant of 189.2 Hz suggested a slightly stronger σ-donating character of **8** compared to **7** [34]. Treatment of **11** with an equiv. amount of methyllithium or lithium diisopropylamide at low temperatures resulted in the formation of a single new product, as evidenced by a new signal at 6.6 ppm in the ^31^P NMR spectrum (Scheme 2). Isolation again revealed selective deprotonation at the cyclohexyl group instead of formation of the desired carbene species. Phosphorus ylide **12** could be isolated in 60% yield as colorless solid. It was characterized by a doublet at 12.0 ppm with a coupling constant of 125.0 Hz in the ^13^C{^1^H} NMR spectrum corresponding to the ylidic carbon atom.

Single crystals of **12** were grown by diffusion of *n*-hexanes into a solution of **12** in benzene (Figure 4). The ylide crystallized in the *P*2_1_/c space group and confirmed the nature of the compound suggested by NMR spectroscopy. The ylidic carbon atom was—as expected for a non-stabilized ylide—pyramidalized, albeit the deviation from planarity was only small, with a sum of angles of 355.9(3)° (Table 1). In comparison to its protonated precursor **11**, **12** exhibited a shortened P-C29 bond due to the ylidic bonding situation, while the P1–C2 bond slightly elongated due to the weaker electrostatic interaction between the onium group and the π-electrons. Despite this weaker ylidic interaction in the P1–C2 linkage of **12**, the C1–C2 bond in **12** was slightly elongated compared to **11**, while the C–N bond changed insignificantly.

### 2.2. Computational Studies

**Figure 5:**
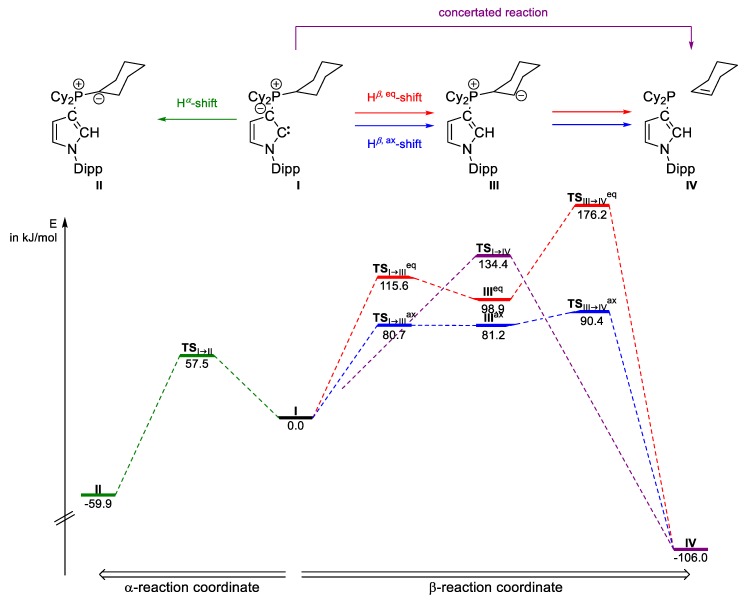
Different pathways for the decomposition of a PCy_3_-substituted CAYC. Energies are given relative to CAYC **I** (PBE0-def2svp/PBE0-def2tzvp; PCM with THF as solvent).

The unsuccessful formation of the CAYCs **7** and **8** led us to more closely look into the stability of different CAYCs. We particularly wondered whether there are stable CAYCs, or if proton shifts from any moiety in the carbene to the divalent carbon center is always energetically favorable. Thus, the energetics of possible proton transfers in the carbene were examined using computational methods. The calculations were performed on the PBE0-def2svp/PBE0-def2tzvp level of theory, including THF as solvent via the polarizable continuum model. At first, decomposition pathways for carbene **I** (onium group = PCy_3_ = **8**) were investigated (Figure 5). Besides the proton shift from the α-position of the onium group to the carbene center (via **TS**_I→II_) to form the ylide observed in experiment, we also probed the viability of a deprotonation in β-position (via **TS**_I→III_). For this β-deprotonation, three different pathways were considered: (i) abstraction of a proton in equatorial position and (ii) in axial position of the cyclohexyl ring followed by cyclohexene elimination (**TS**_III→IV_), as well as (iii) a concerted mechanism, in which proton abstraction and cyclohexene elimination occur simultaneously (**TS**_I→IV_). The results showed that from a thermodynamic point of view, both the α- and β-deprotonation (with subsequent cyclohexene elimination) were clearly favored over the free carbene **I**. Thereby, the cyclohexene elimination product was the most favored decomposition product, being 106 kJ/mol favored over the AYC and 46 kJ/mol over ylide **II** (onium group = PCy_3_ = **11**). This preference was expected, since the β-elimination product **IV** is lacking any reactive carbenic, ylidic or carbanionic site in the molecule and thus should be more stable. However, kinetically the transfer of the proton in α-position to the phosphorus atom was favored over the abstraction of the β-hydrogen atom, so that **II** was the kinetically favored species. With a barrier of only 58 kJ/mol, the required activation energy for TSα was small enough to be easily overcome at temperatures below room temperature, thus explaining the impossible isolation of **8** and the selective formation of **12**.

Next, detailed studies on the decomposition pathways with different onium moieties were performed to unveil molecular structures advantageous for the isolation of stable CAYCs. Besides phosphonium, sulfonium and sulfoxonium units with different alkyl substituents were also considered. The results for the different CAYCs **I** are summarized in Table 2. It should be noted that in some cases, the β-deprotonated species **III** was found to be not stable and decomposed directly under formation of the alkene and the corresponding sulfide, sulfoxide and phosphine, respectively (concerted mechanism). The calculations revealed that in every case, the deprotonation in α-position to the heteroatom was thermodynamically favored over the corresponding CAYC. For example, α-deprotonation at a cyclohexyl group was favored by approx. 60 kJ/mol for phosphonium, sulfonium, and sulfoxonium groups. Furthermore, the activation energy for the H_α_-shift from the methyl or cyclohexyl group to the carbene center was always low enough to be easily overcome at room temperature. Thus, substituents with *α*-H atoms seem to be unsuitable for the formation of isolable CAYCs.

Therefore, we turned our attention toward the α-hydrogen-free substituents, *tert*-butyl and adamantyl, where only deprotonation in β-position to the heteroatom can take place. In case of the *tert*-butyl groups, the β-deprotonated species **IV** were clearly thermodynamically favored over the CAYCs. However, the corresponding shifts of the H^β^-atoms were kinetically less favored then the H^α^-shifts. This was at least suggested by the results obtained from the cyclohexyl-substituted ylides. However, in the case of the *tert*-butyl substituents, the barriers for the β-deprotonation also appeared to be very low, so any attempt to isolate a *tert*-butyl-substituted CAYC **I** should lead to the formation of isobutene and the corresponding pyrrole. The same holds true for the compounds with cyclic sulfonium groups. The only exceptions are the adamantly-substituted compounds. Here, the products formed after deprotonation and elimination were thermodynamically only slightly downhill (sulfonium ylide) or even uphill. This was due to the required formation of a double bond at the bridgehead of the adamantly substituents (Bredt’s rule). Overall, the tris(adamantyl) phosphonium-substituted CAYC is thus the most promising candidate for isolation. It features a rather high barrier for intramolecular proton transfer and β-elimination is clearly disfavored by 47 kJ/mol.

## 3. Discussion and Conclusions

In conclusion, we reported herein on attempts to synthesize and isolate room-temperature-stable CAYCs. While former studies solely focused on aryl-substituted onium moieties, which partly underwent decomposition by cleavage of one of the aryl groups, we examined the impact of alkyl substituents on stability. The experimental and theoretical results clearly showed that the isolation of trialkyl-onium-substituted CAYCs is also preparatively highly challenging. Simple alkyl groups such as methyl or cyclohexyl are not suitable substituents due to their straightforward deprotonation in the α-position to the heteroatom. Thus, in case of the PCy_3_-substituted pyrrole, no carbene but ylide formation via deprotonation at the cyclohexyl moiety was observed. Likewise, the dimethylsulfonium compound underwent a kind of Corey–Chaykovsky reaction with formation of ethene and the thioether. The calculations demonstrated that even if accessible at low temperatures, trialkyl onium groups show high tendencies to undergo proton shifts not only from the α-position of the heteroatom, but also from the β-position. This observation is true for phosphonium, sulfonium, and sulfoxonium moieties. These results, together with the previously observed P-C bond cleavage of aryl-substituted onium groups, clearly show that a careful molecular design must be chosen in order to exclude decomposition pathways of these highly basic carbenes. Our calculations suggest that α-hydrogen-free substituents, particularly adamantyl moieties, might be the substituents of choice in terms of stability. Current experimental studies are now addressing the preparation of CAYCs with a molecular design proposed by our calculations. The steric bulk will presumably require additional structural changes for successful synthesis of the carbene precursors. If these difficulties can be handled, stable CAYCs should become available in the near future.

## 4. Materials and Methods

Detailed experimental procedures, NMR spectra, and crystallographic and computational data as well as the coordinates and energies of the optimized structures can be found in the Supplementary Information File available online.

### 4.1. Crystal Structure Determination

All data were collected with an Oxford *SuperNova*. The structures were resolved using direct methods; the *Shelx* software package [38,39,40] was used for refinement and expansion was carried out using Fourier techniques. An inert oil was used to mount the crystals and the crystal structure determination was performed at 100 K using Mo-K_α_ radiation. The corresponding data have been deposited with the Cambridge Crystallographic Data Centre as supplementary publication no. 1979639-1979642. Copies of the data can be obtained free of charge on application to Cambridge Crystallographic Data Centre, 12 Union Road, Cambridge CB2 1EZ; UK [Homepage: https://www.ccdc.cam.ac.uk/].

*Diamond 4.0* [41] by Crystal Impact and *GIMP* 2.10 [42] were used for graphic representation, Ellipsoids are drawn at the 50% probability level.

The structure of **9** contained a highly disordered solvent molecule that was treated using the PLATON/SQUEZZE routine. [43]

### 4.2. Computational Studies

All computational studies were carried out without symmetry restrictions. If it was not possible to obtain starting coordinates from crystal structures, either *GaussView 3.0* [44] or *GaussView 6.0* [45] were used. Calculations were done either with the *Gaussian09 Revision E.01* [46], the *Gaussian16 Revision B.01* [47], or the *Gaussian16 Revision C.01* [48] program packages using density-functional theory (DFT) [49,50] with PBE0 [51]/def2svp [52] with Grimmes D3 dispersion correction with Becke–Johnson damping [53,54,55]. To determine the nature of the structures, harmonic vibrational frequency analysis was performed on the same level of theory. [56] No imaginary frequencies were observed for the ground states; for transition states, one imaginary frequency corresponding to the translational motion was observed. Single point energies were calculated on PBE0 [51]/def2tzvp [52] level of theory. Additional, single-point energies were calculated on the same level of theory with the polarizable continuum model (PCM) the integral equation formalism variant (IEFPCM) [57], as implemented in Gaussian09 and Gaussian16 with the parameters for tetrahydrofurane. The values in the paper are by 7.925867 kJ/mol for each species to convert them to a 1 M standard state.

## Figures and Tables

**Table 1:** Comparison of the bond lengths in **11** and **12**.

Structural Parameters		11	12
bond length (Å)	C1–C2	1.358(4)	1.379(2)
	C1-N1	1.358(4)	1.364(1)
	C2–P1	1.765(3)	1.790(1)
	P1–C29	1.830(3)	1.679(1)
bond angle (°)	P1–C29–C30	110.9(2)	122.20(10)
	P1–C29–C34	111.1(2)	120.68(10)
	C30–C29–C34	110.0(2)	113.02(11)
	∑	332.0(6)	355.90(31)

**Table 2:** Comparison of the proton shifts from the α- and β-position of the onium group to the carbene center. Energies are given relative to the corresponding CAYC **I** (PBE0-def2svp/PBE0-def2tzvp; PCM with THF as solvent).

Onium Group	II (kJ/mol)	TS_I__→II_ (kJ/mol)	TS_I__→III_ (kJ/mol)	III (kJ/mol)	TS_III__→IV_ (kJ/mol)		IV (kJ/mol)
	α-deprotonation		β-deprotonation				
SCy_2_	−61.4	44.1	66.9 (concerted)			−239.9	
SOCy_2_	−61.7	50.6	102.7 (concerted)			−203.5	
			100.1^eq^	107.4^eq^	119.1^eq^		
PCy_3_	−59.9	57.5	134.4 (concerted)			−106.0	
			115.6^eq^	98.9^eq^	176.2^eq^		
			80.7^ax^	81.2^ax^	90.4^ax^		
SMe_2_	−52.2	54.8	–				
SOMe_2_	−85.0	60.3	–				
PMe_3_	−53.9	56.5	–				
S*^t^*Bu_2_	–		54.0 (concerted)			−236.3	
SO*^t^*Bu_2_	–		35.9 (concerted)			−248.0	
P*^t^*Bu_3_	–		71.1	40.9	58.5	−185.5	
SAd_2_	–		123.7			−8.3	
SOAd_2_	–		102.4			7.4	
PAd_3_	–		91.6	91.2	108.4	47.1	
S(CH_2_)_2_	–		38.0^a^	−48.6 ^a^	−40.4 ^a^	−275.0	
S(CH_2_)_3_	−11.9	65.5	105.0	75.4	83.0	−238.8	
S(CH_2_)_4_	−33.6	56.7	90.5 (concerted)			−180.0	

[a] Note that deprotonation always occurs in the α-position to sulfur. Thus, activation energies are lower than those usually seen for β-deprotonations.

## Supplementary Materials

The following are available online. Supporting information on experimental procedures, NMR spectra, and crystallographic and computational data as well as the coordinates and energies of the optimized structures.
