# Supplementary material for: Towards the Preparation of Stable Cyclic Amino(ylide)Carbenes
Source: Molecules. 2020 Feb 12;25(4):796. doi: 10.3390/molecules25040796 (PMC7070660; doi:10.3390/molecules25040796)
Supplement: Supplementary file 1 [file molecules-25-00796-s001.pdf]

Supporting Information to

Toward the Preparation of Stable Cyclic Amino(ylide)-carbenes

Henning Steinert, Christopher Schwarz, Alexander Kroll and Viktoria H. Gessner*

[1] Faculty of Chemistry and Biochemistry, Chair of Inorganic Chemistry II, Ruhr University Bochum, Universitätsstr. 150, 44801 Bochum (Germany)

* Correspondence: viktoria.daeschlein-gessner@rub.de

Table of contents

1. Synthetic procedures ... 2
- 1.1. N-(2,6-Diisopropylphenyl)-pyrrole ... 2
- 1.2 Dimethyl-(N-(2,6-Diisopropylphenyl)pyrrole)sulfonium tetrafluoroborate **6** ... 2
- 1.3 N-(2,6-Diisopropylphenyl)-3-methylthiopyrrol **8** ... 5
- 1.4 N-(2,6-Diisopropylphenyl)-3-iodopyrrole ... 8
- 1.5 Tricyclohexyl-(N-(2,6-diisopropylphenyl)pyrrol)phosphonium iodide **9** ... 9
- 1.6 Dipp-Pyrr-PCy_2=C_6H_{10} **11** ... 11

2. Crystal Structure Determination ... 14
- 2.1 Crystal data and structure refinements ... 14
- 2.2 N-(2,6-diisopropylphenyl)pyrrole ... 16
- 2.3 [Dipp-Pyrr-SMe_2][$BF]_4$ **6** ... 17
- 2.4 [Dipp-Pyrr-PCy_3]I **9** ... 19
- 2.5 Dipp-Pyrr-PCy_2=C_6H_{10} **11** ... 21

3. Computational Studies ... 24
- 3.1. General ... 24
- 3.2. Energies and coordinates for the AYC with a SCy2 onium moiety ... 24
- 3.3 Energies and coordinates for the AYC with a $SOCy_2$ onium moiety ... 32
- 3.4 Energies and coordinates for the AYC with a PCy_3 onium moiety ... 44
- 3.6 Energies and coordinates for the AYC with a SMe_2 onium moiety ... 63
- 3.7 Energies and coordinates for the AYC with a $SOMe_2$ onium moiety ... 66
- 3.8 Energies and coordinates for the AYC with a PMe_2 onium moiety ... 69
- 3.9 Energies and coordinates for the AYC with a $StBu_2$ onium moiety ... 73
- 3.10 Energies and coordinates for the AYC with a $SOtBu_2$ onium moiety ... 77
- 3.11 Energies and coordinates for the AYC with a $PtBu_3$ onium moiety ... 81
- 3.12 Energies and coordinates for the AYC with a SAd_2 onium moiety ... 89
- 3.13 Energies and coordinates for the AYC with a $SOAd_2$ onium moiety ... 95
- 3.14 Energies and coordinates for the AYC with a PAd_3 onium moiety ... 100
- 3.15 Energies and coordinates for the AYC with a $S(CH_2)_2$ onium moiety ... 112

4. Literature ... 129

1. Synthetic procedures

1.1. *N-(2,6-Diisopropylphenyl)-pyrrole*

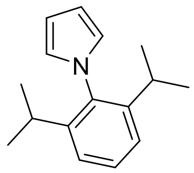

The compound was synthesized similar to a known procedure.[1]

18.3 mL (18.68 g, 145.81 mmol, 1.1 eq) of tetrahydro-2,5-dimethoxyfurane and 25.0 mL (23.50 g, 132.55 mmol, 1.0 eq) of 2,6-diisopropylaniline were solved in 50 mL glacial acetic acid and refluxed for 50 minutes at 130 °C, which led to the formation of a dark brown solution. Afterwards, 100 mL of water were added and the solution was extracted three times with 100 mL of dichloromethane. The combined organic layers were extracted with 400 mL of a concentrated sodium carbonate solution. After removal of all volatile compounds *in vacuo* the resulting dark brown colored solid was purified on a short silica column with cyclohexane/ethyl acetate (9:1). The solvent was removed *in vacuo* and the crude product was multiple times recrystallized from methanol. The pyrrole could be obtained as a colorless crystalline solid in 83 % (25.07 g, 110.28 mmol) yield.

^{1}H NMR (400.3 MHz, RT, $CDCl_3$): δ = 1.14 (d, $^{3}J_{HH}$ = 6.9 Hz, 12 H, 4 CH_3), 2.44 (hept, $^{3}J_{HH}$ = 6.9 Hz, 2 H, $CHMe_2$), 6.32 (t, $^{3}J_{HH}$ = 2.1 Hz, 2 H, 2 CH_{Pyr}), 6.64 (t, $^{3}J_{HH}$ = 2.1 Hz, 2 H, 2 CH_{Pyr}), 2.72-2.74 (m, 2 H, m-CH_{Aryl}), 7.34-7.43 (m, 1 H, p-CH_{Ar}).

^{13}C{^{1}H} NMR (100.7 MHz, RT, $CDCl_3$): δ = 24.7 (4 CH_3), 28,2 (2 $CHMe_2$), 108.3 (2 CH_{Ar}), 123.2 (2 CH_{Ar}), 123.5 (2 CH_{Ar}), 129.0 (p-CH_{Ar}), 137.1 (*i*-$C_{q, Ar}$), 147.1 (2 *o*-$C_{q, Ar}$).

The data are in good accordance to the literature.[1]

1.2 Dimethyl-(N-(2,6-Diisopropylphenyl)pyrrole)sulfonium tetrafluoroborate ***9***

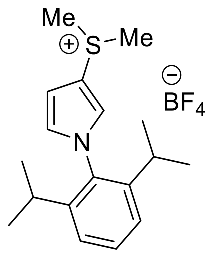

The compound was synthesized similar to a known procedure.[2–4]

A solution of 0.75 mL (825.0 mg, 10.56 mmol, 1.00 eq) dimethyl sulfoxide and 2.40 g (10.56 mmol, 1.00 eq) *N*-(2,6-diisopropylphenyl)pyrrole in 5 mL dichloromethane was cooled to –60 °C and 1.47 mL (2.22 g, 10.56 mmol, 1.00 eq) trifluoro acetic acid anhydride were added. Afterwards, the solution was slowly warmed to room temperature and the solution was extracted with 10 mL of a concentrated aqueous sodium tetrafluoroborate solution. After extraction of the aqueous phase with dichloromethane (3 x 10 mL) the combined organic layers were dried over magnesium sulphate. The volatile compounds were removed *in vacuo* and the crude product crystallized from dichloromethane/diethyl ether (1:1). The product was obtained as colourless crystalline solid in 58% (2.32 g, 10.56 mmol) yield.

Melting Point: 152.3 °C.

^{1}H NMR (400.3 MHz, RT, $CDCl_3$): δ = 1.13 (d, $^{3}J_{HH}$ = 7.1 Hz, 6 H, 2 CH_3), 1.15 (d, $^{3}J_{HH}$ = 7.1 Hz, 6 H, 2 CH_3), 2.27 (hept, $^{3}J_{HH}$ = 6.8 Hz, 2 H, $CHMe_2$), 3.32 (s, 6 H, 2 SCH_3), 6.89 (dd, $^{3}J_{HH}$ = 3.1 Hz, $^{4}J_{HH}$ = 2.1 Hz, 1 H, $NCH_{Pyr}CH_{Pyr}C_{q, Pyr}$), 6.94 (dd, $^{3}J_{HH}$ = 3.1 Hz, $^{4}J_{HH}$ = 2.1 Hz, 1 H, $NCH_{Pyr}CH_{Pyr}C_{q, Pyr}$), 7.25 (d, $^{3}J_{HH}$ = 7.8 Hz, 2 H, *m*-CH_{Ar}), 7.35 (t, $^{4}J_{HH}$ = 2.0 Hz, 1 H, $NCHC_q$), 7.45 (t, $^{3}J_{HH}$ = 7.8 Hz, 1 H, *p*-CH_{Ar}).

^{11}B{^{1}H} NMR (162.1 MHz, RT, $CDCl_3$): δ = -0.9.

$^{13}C\{^1H\}$ NMR (100.7 MHz, RT, $CDCl_3$): δ = 24.5, 24.6 (2 CH(CH$_3$)$_2$), 28.5 (2 CH(CH$_3$)$_2$), 31.4 (S(CH$_3$)$_2$), 102.8 ($C_{q,\,Pyr}$), 108.2 (NCH$_{Pyr}$CH$_{Pyr}$C$_{q,\,Pyr}$), 124.2 (m-CH$_{Ar}$), 128.5 (NCH$_{Pyr}$CH$_{Pyr}$C$_{q,\,Pyr}$), 128.9 (NCHC$_q$), 130.5 (p-CH$_{Ar}$), 134.7 (i-C$_{q,}$ Ar), 146.2 (o-C$_{q,}$ Ar).

$^{19}F\{^1H\}$ NMR (235.3 MHz, RT, $CDCl_3$): δ = -150.5.

IR (ATR): 3147 (w), 3118 (w), 3041 (w), 2960 (w), 2866 (w), 1508 (m), 1497 (m), 1433 (m), 1352 (m), 1159 (w), 1088 (s), 1054 (vs), 1027 (vs), 966 (s), 933 (m), 802 (s), 758 (s), 716 (m), 651 (m), 623 (m), 520 (m), 479 (w), 459 (w).

Elemental analysis for $C_{18}H_{26}BF_4NS$. Calculated: C 64.84, H 7.98, N 2.52, S 5.77; Found: C 65.13, H. 7.77, N 2.59, S 5.46.

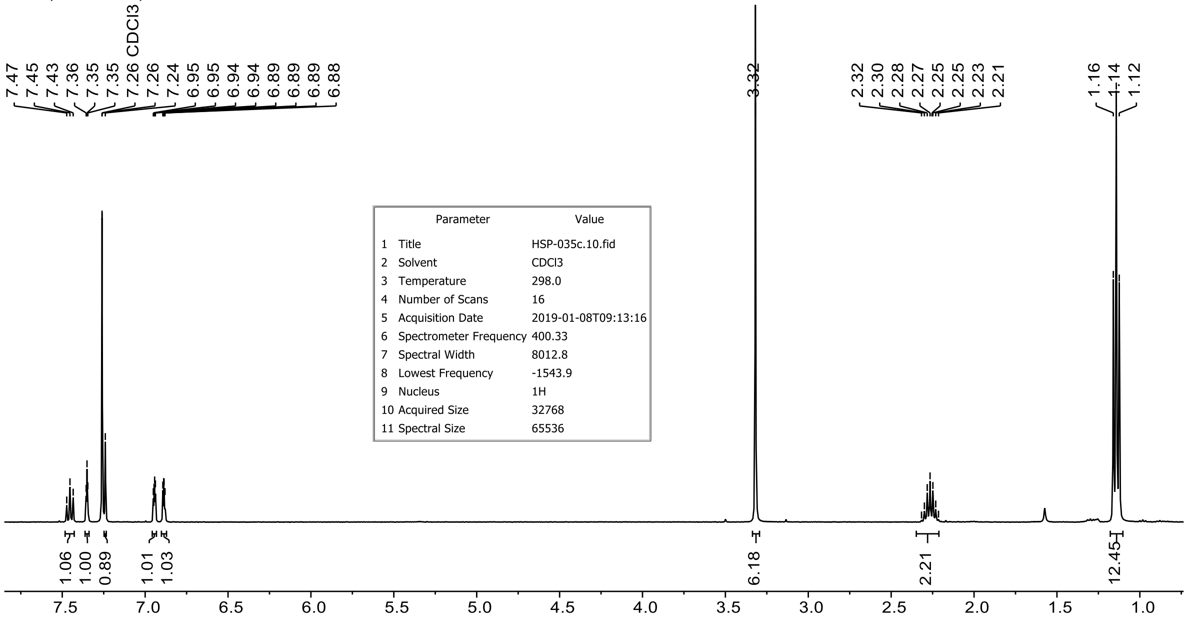

Figure 1. ^1H NMR spectrum of **9** in $CDCl_3$.

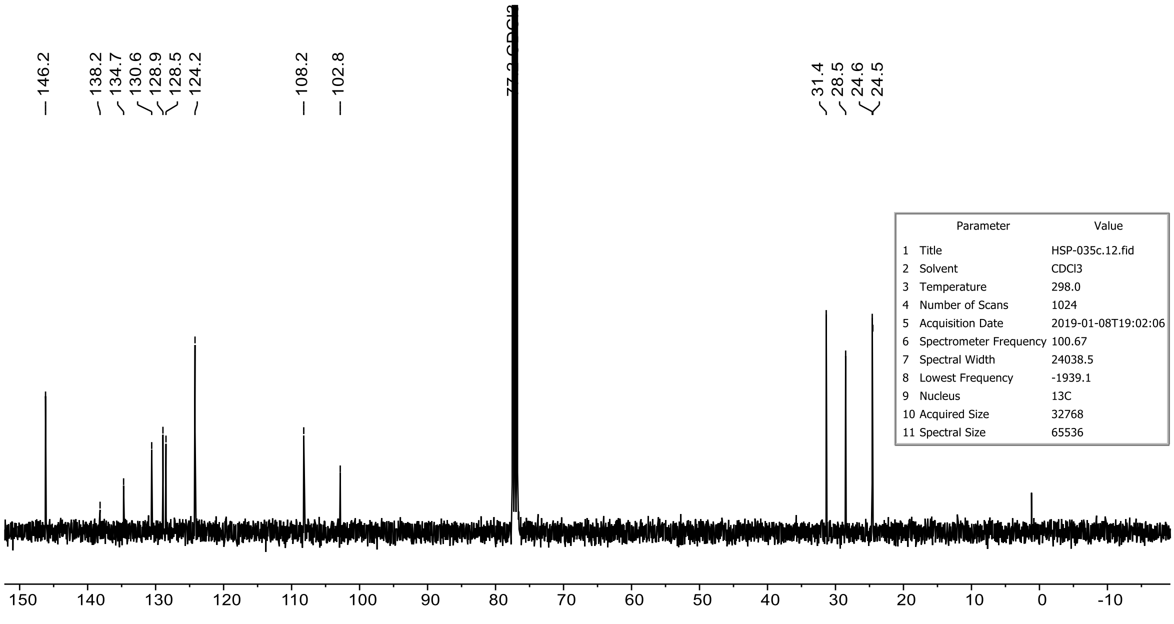

Figure 2. $^{13}C\{^1H\}$ NMR spectrum of **9** in $CDCl_3$.

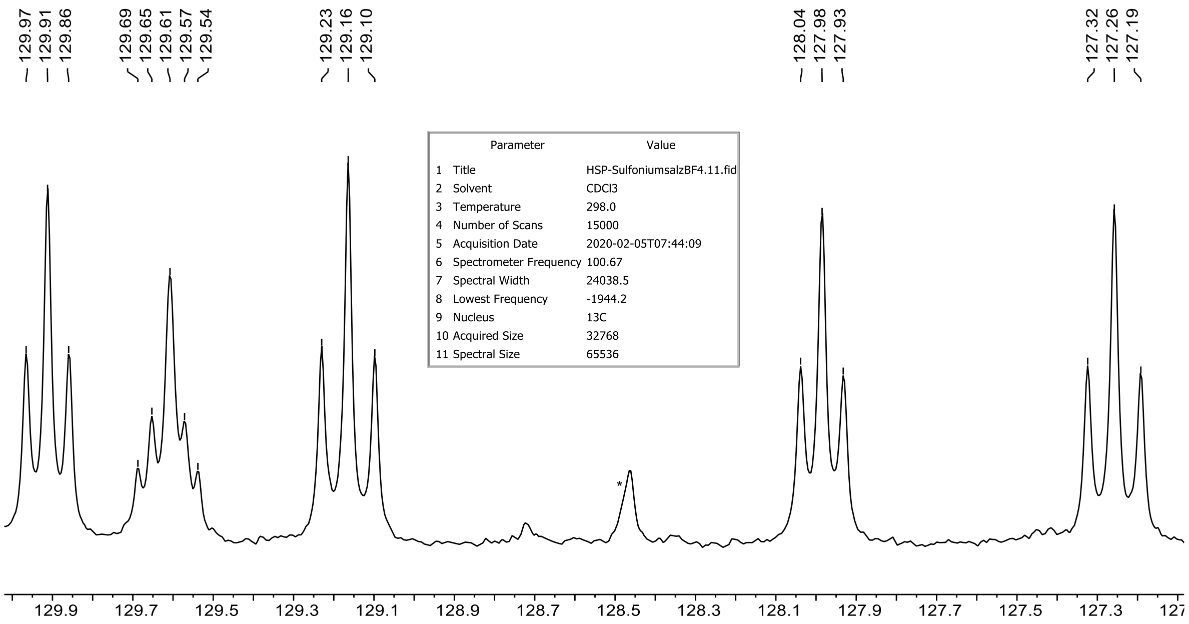

Figure 3. Section of the ^{13}C NMR spectrum of **9** in $CDCl_3$ (* = impurity).

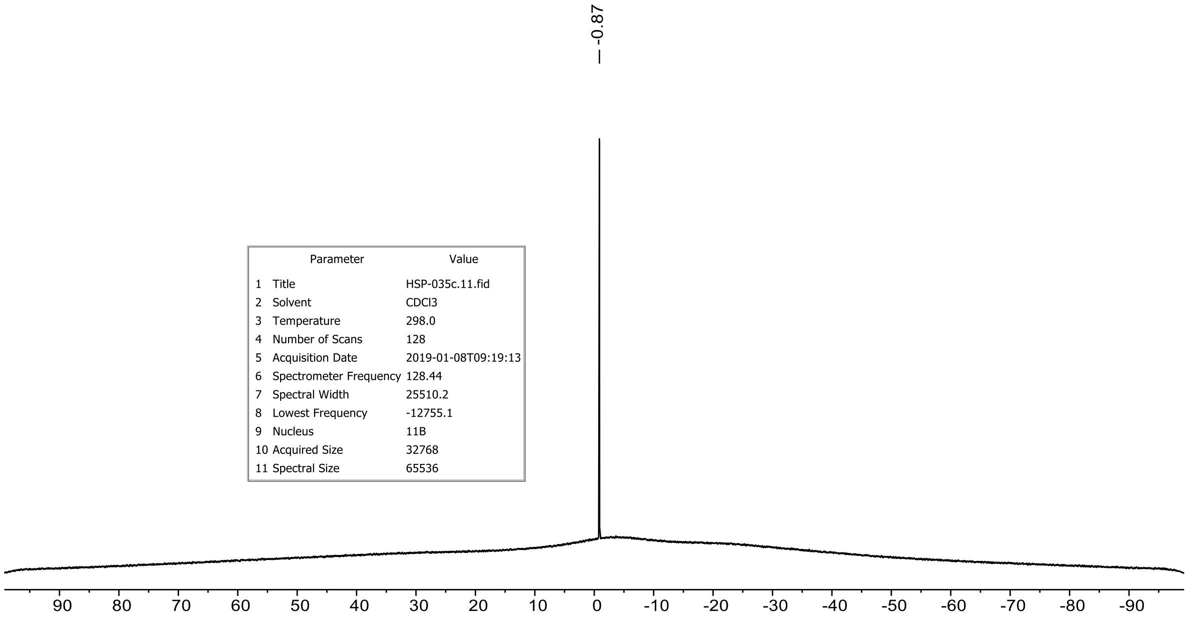

Figure 4. ^{11}B{^{1}H} NMR spectrum of **9** in $CDCl_3$.

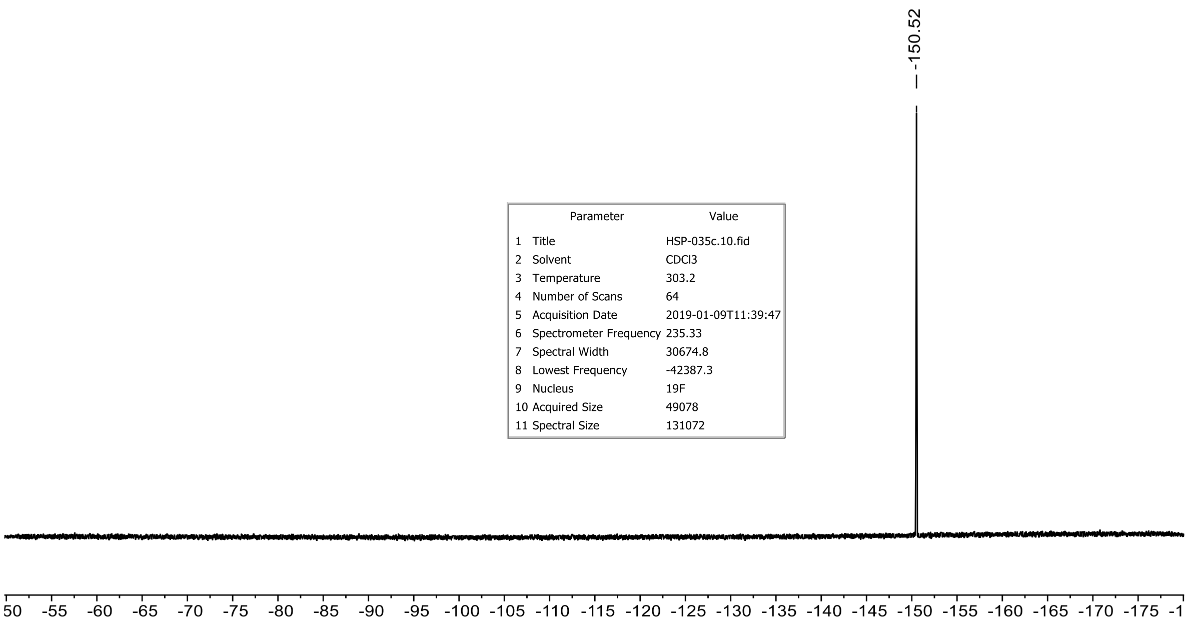

Figure 5. $^{19}F\{^1H\}$ NMR spectrum of **9** in $CDCl_3$.

1.3 N-(2,6-Diisopropylphenyl)-3-methylthiopyrrol ***10***

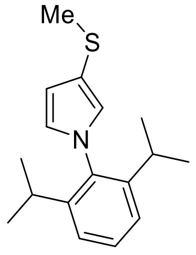

A) A solution of 1.14 g (3.04 mmol, 1.00 eq) dimethyl-(*N*-(2,6-diisopropylphenyl)pyrrol)-sulfonium tetrafluoroborate **9** in 10 mL THF was cooled to -78 °C and 3.40 mL (68.74 mg, 3.13 mmol, 1.03 eq) of a 0.92 M methyllithium solution in diethyl ether were added. After this, the solution turned brown, a solid precipitated and an evolution of gas was observed. After slowly warming the mixture to room temperature, the reaction was stirred for another 16 h. All volatile compounds were removed *in vacuo* and the brown crude product was purified by column chromatography (SiO_2, *n*-hexane/ethylacetate gradient). The product was thus obtained as colourless oil in 47 % (0.39 g, 1.42 mmol) yield.

B) A solution of 75.06 mg (200.0 µmol, 1.00 eq) dimethyl-(*N*-(2,6-diisopropylphenyl)pyrrol)-sulfonium tetrafluoroborate **9** in 2.5 mL THF was cooled to -78 °C and 0.10 mL (21.43 mg, 200.0 µmol, 1.00 eq) of a 2.0 M lithium diisopropylamine solution in tetrahydrofurane/heptane/ethylbenzene were added. After this, the solution turned brown and a solid precipitated. After warming the mixture to room temperature, the reaction was stirred for another 16 h. All volatile compounds were removed *in vacuo* and the brown crude product was dissolved in toluene and filtered. After removal of all volatile compounds the product was thus obtained as colourless oil in 94 % (51.6 mg, 188.7 µmol) yield.

^1H NMR (400.3 MHz, RT, C_6D_6): δ = 0.97-1.07 (m, 12 H, 2 CH(C*H*$_3$)$_2$), 2.21 (s, 3 H, SC*H*3), 2.56 (hept, $^3J_{HH}$ = 6.9 Hz, 2 H, C*H*Me$_2$), 6.47 (d, ^4J = 1.9 Hz, 2 H, NC*H*C*H*C$_q$), 6.69 (t, 4J = 1.9 Hz, 1 H, NC*H*C$_q$), 7.01 (d, 3J = 7.7 Hz, 2 H, 2 *m*-C*H*$_{Ar}$), 7.13-7.20 (m, 1 H, *p*-C*H*$_{Ar}$).

$^{13}C\{^1H\}$ NMR (100.7 MHz, RT, C_6D_6): δ = 20.4 (SCH_3), 24.5, 24.7 (2 CH(CH_3)$_2$), 28.4 (2 CH(CH_3)$_2$), 112.5 (CH$_{Pyr}$), 117.1 (C$_{q, Pyr}$), 123.2 (2 *m*-CH$_{Ar}$), 124.2 (CH$_{Pyr}$), 124.5 (NCH$_{Pyr}$C$_{q, Pyr}$), 129.5 (*p*-CH$_{Ar}$), 137.2 (2 *i*-C$_{q, Ar}$), 147.1 (*o*-C$_{q, Ar}$).

IR (ATR): 2963 (vs), 2924 (m), 2868 (m), 1490 (vs), 1475 (m), 1458 (m), 1384 (w), 1364 (m), 1355 (w), 1298 (w), 1254 (w), 1211 (w), 1178 (w), 1155 (w), 1105 (w), 1072 (m), 1058 (m), 1033 (w), 969 (w), 934 (m), 806 (m), 781 (m), 763 (s), 704 (w), 650 (w), 633 (w).

Elemental analysis for $C_{17}H_{23}NS$. Calculated: 64.67 % C, 8.46 % H, 5.12 % N, 11.72 % S; Found: 74.60 % C, 8.48 % H, 5.30 % N, 11.37 % S.

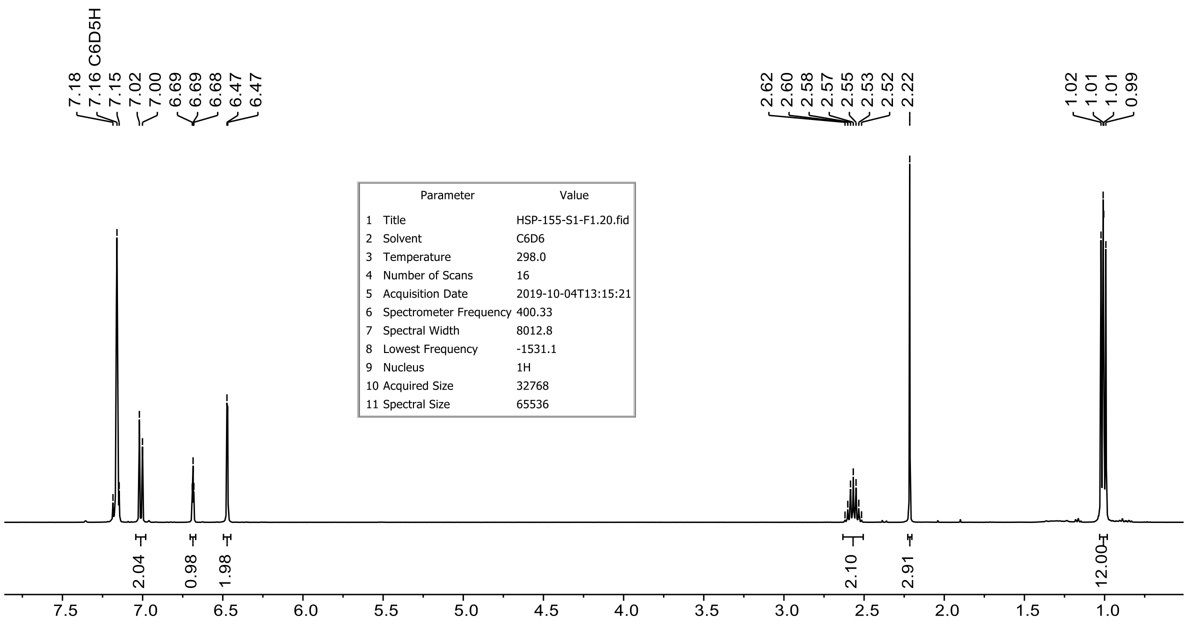

Figure 6. ^{1}H NMR spectrum of **10** in C_6D_6.

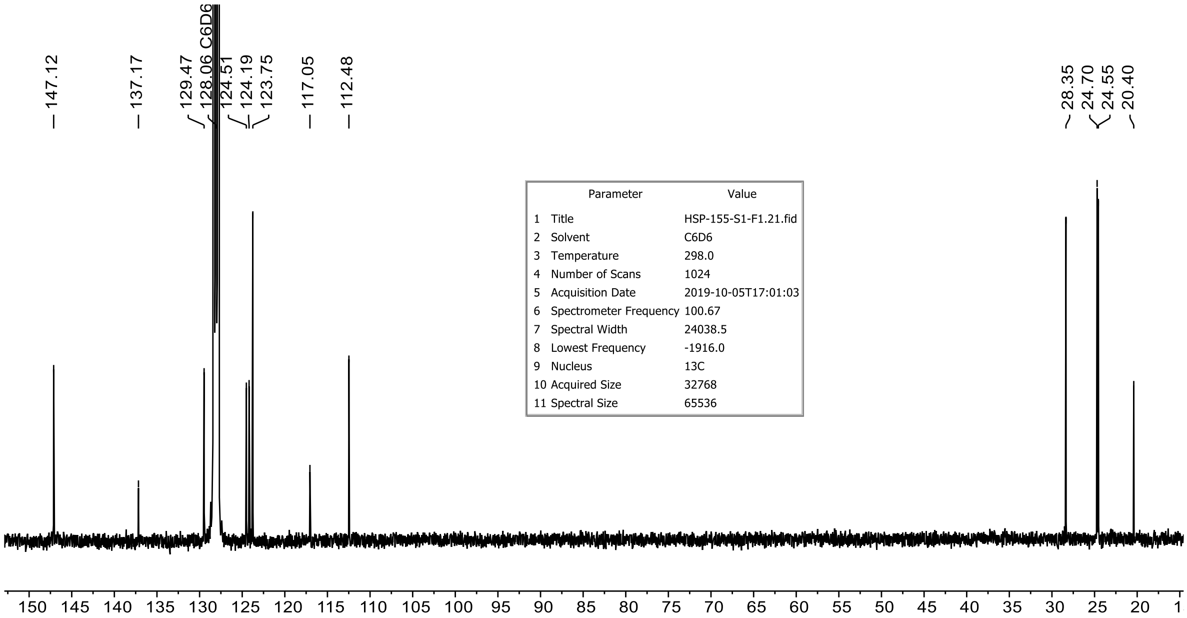

Figure 7. $^{13}C\{^{1}H\}$ NMR spectrum of **10** in C_6D_6.

For detection of ethylene, 47.50 mg (126.57 µmol, 1.0 eq) of **6** and 2.78 mg (126.57 µmol, 1.0 eq) methyllithium were placed in a J. Young NMR tube. After addition of 0.6 mL tetrahydrofurane-*d8* the NMR tube was closed immediately and spectra were recorded.

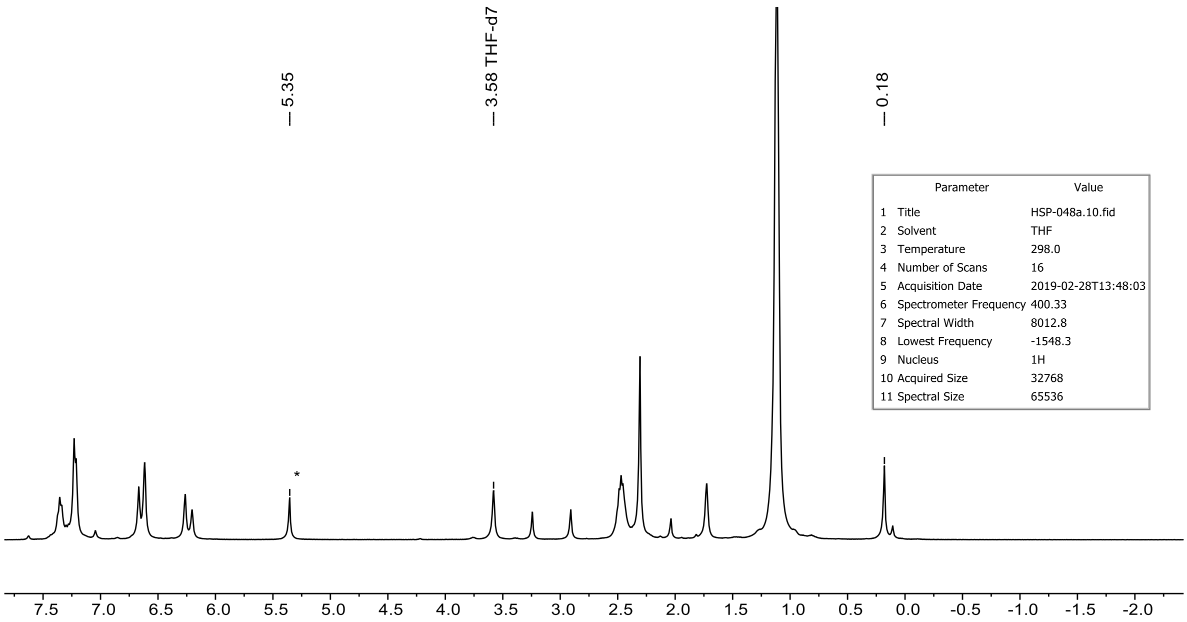

Figure 8. *In situ* ^{1}H NMR of the synthesis of **10** in THF-*d8* (* = ethylene).

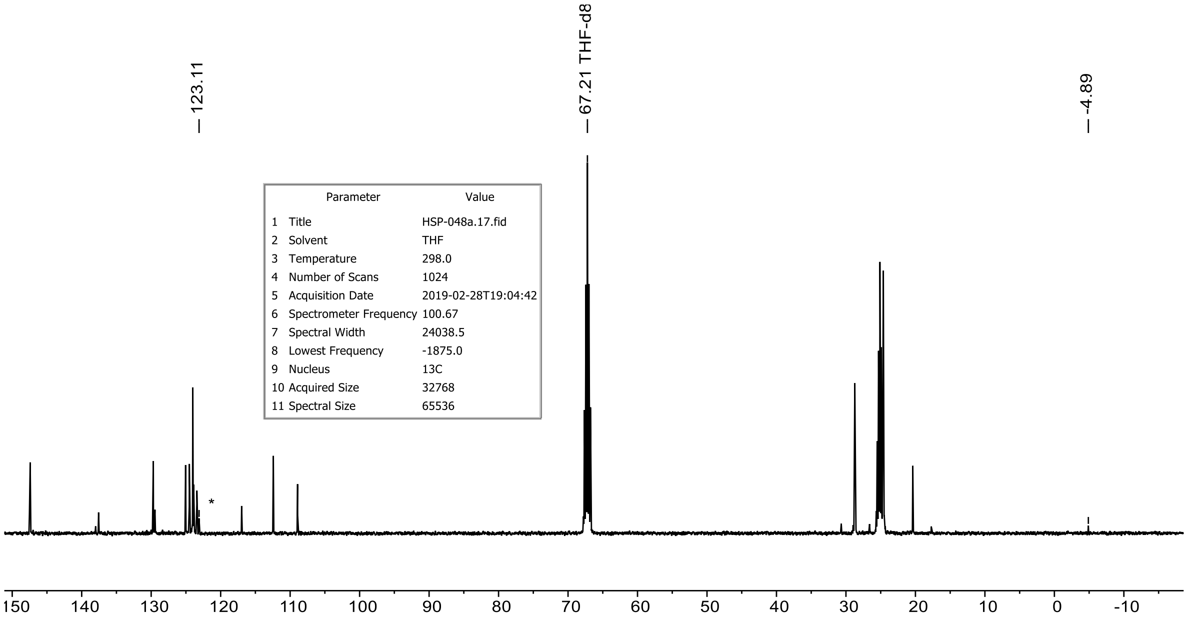

Figure 9. *In situ* ^{13}C{^{1}H} NMR spectrum of **10** in THF-*d8* (* = ethylene).

1.4 N-(2,6-Diisopropylphenyl)-3-iodopyrrole

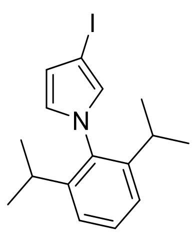

The compound was synthesized similar to a known procedure.[5]
At -70 °C, 2.00 g (8.80 mmol, 1.00 eq) *N*-(2,6-diisopropylphenyl)pyrrole and 2.38 g (10.56 mmol, 1.20 eq) *N*-iodosuccinimide were dissolved in 30 mL dichlormethane. The solution was slowly warmed to room temperature and stirred for another 16 h. After removal of all volatile compounds *in vacuo* the crude product was suspended in *n*-hexane and filtered over neutral alumina. 2.35 g (6.65 mmol) of the product were obtained as mixture of the 2-/3-iodo regioisomers in a ratio of 7/1 as a colorless oil in 85 % (2.35 g, 6.65 mmol) yield.
^{1}H NMR (400.3 MHz, RT, $CDCl_3$): δ = 1.12 (d, $^3J_{HH}$ = 6.7 Hz, 6 H, 2 CH(C*H*$_3$)$_2$), 1.14 (d, $^3J_{HH}$ = 6.7 Hz, 6 H, 2 *i*PrCH$_3$), 2.44 (hept, $^3J_{HH}$ = 6.9 Hz, 2 H, 2 C*H*(CH$_3$)$_2$), 6.39 (dd, J_{HH} = 1.6 Hz, J_{HH} = 2.8 Hz, 1 H, C*H*$_{Pyr}$), 6.50-6.52 (m, 1 H, C*H*$_{Pyr}$), 6.67 (dd, J_{HH} = 1.6 Hz, J_{HH} = 2.2 Hz, 1 H, C*H*$_{Pyr}$), 7.21 (d, ^3J = 7.7 Hz, 2 H, 2 *m*-C*H*$_{Ar}$), 7.39 (t, ^3J = 7.8 Hz, 1 H, *p*-C*H*$_{Ar}$).
^{13}C{^{1}H} NMR (100.7 MHz, RT, $CDCl_3$): δ = 24.6 (2 CH(*C*H$_3$)$_2$), 24.8 (2 CH(*C*H$_3$)$_2$), 28.14 (2 *C*H(CH$_3$)$_2$), 59.8 (*C*I), 116.0 (*C*H$_{Pyr}$), 123.7 (2 *m*-*C*H$_{Ar}$), 125.1 (*C*H$_{Pyr}$), 127.5 (*C*H$_{Pyr}$), 129.5 (*p*-*C*H$_{Ar}$), 136.3 (*i*-*C*$_{q,\,Ar}$), 147.0 (2 *o*-*C*$_{q,\,Ar}$).
IR (ATR): 2962 (s), 2926 (m), 2867 (m), 1487 (s), 1475 (m), 1458 (m), 1383 (m), 1363 (m), 1342 (s), 1293 (m), 1255 (w), 1179 (w), 1071 (m), 1058 (m), 1032 (w), 936 (m), 903 (s), 805 (m), 762 (s), 701 (m), 649 (m), 606 (w).
Elemental analysis for $C_{16}H_{20}NI$. Calculated: 54.40 % C, 5.71 % H, 3.97 % N; Found: 54.25 % C, 5.70 % H, 4.16 % N.
HRMS (EI): calculated for $C_{16}H_{20}NI^+$ ([M]$^+$): *m*/*z* = 353.06404, found: *m*/*z* = 353.06250.

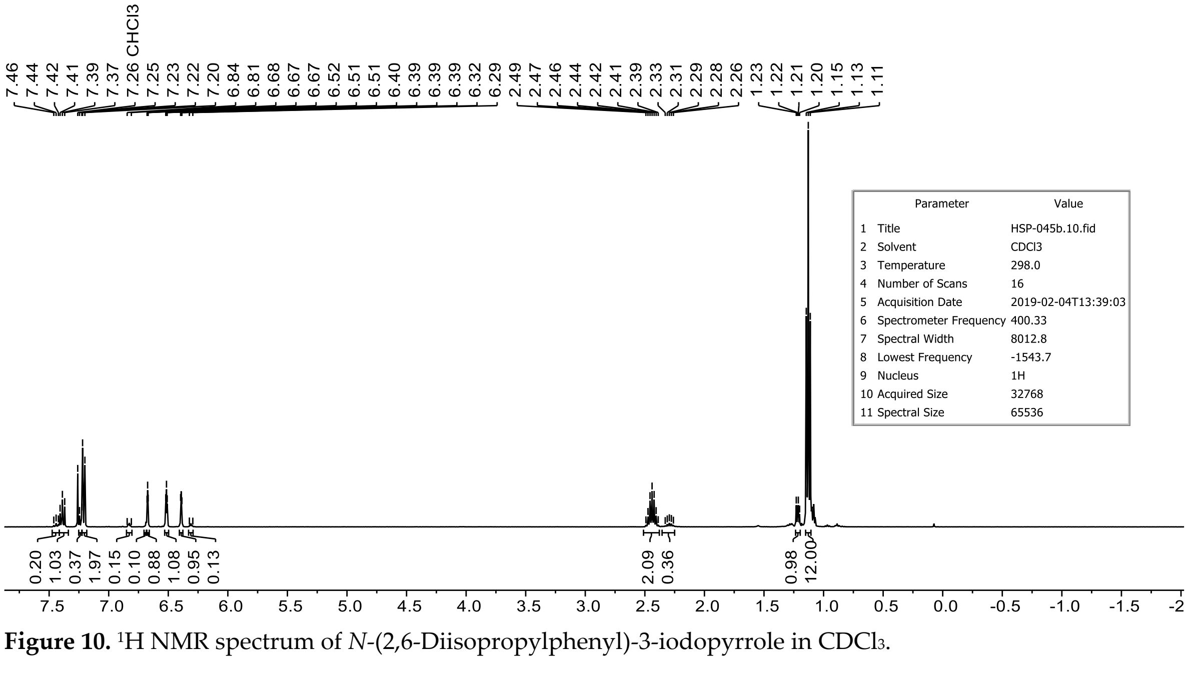

Figure 10. ^{1}H NMR spectrum of *N*-(2,6-Diisopropylphenyl)-3-iodopyrrole in $CDCl_3$.

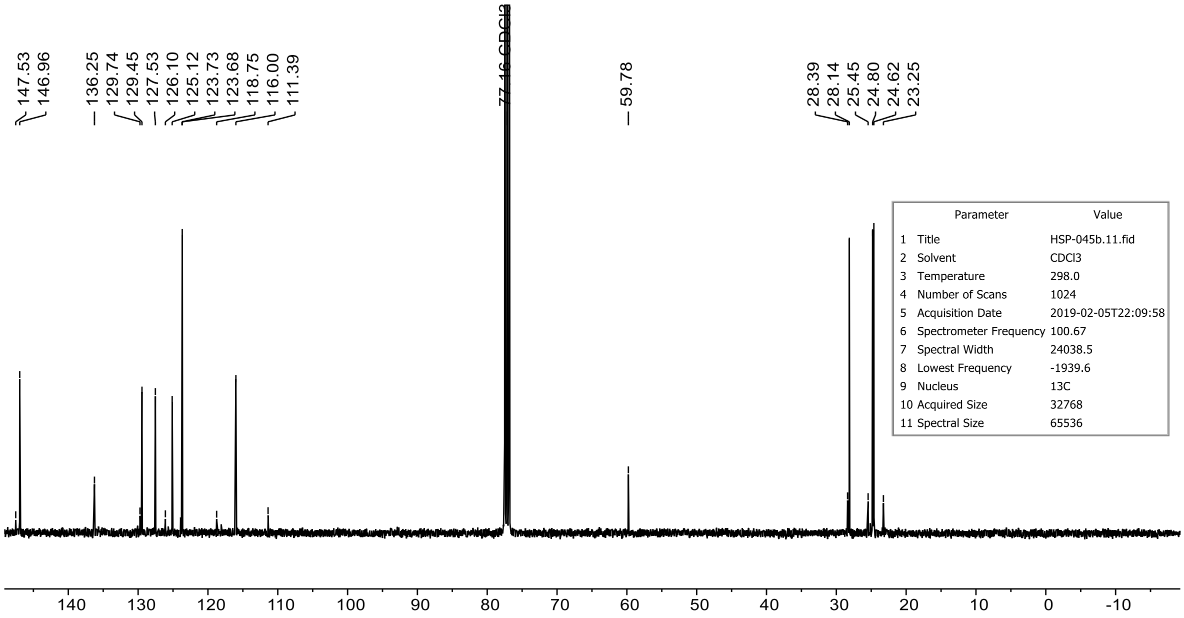

Figure 11. $^{13}C\{^{1}H\}$ NMR spectrum of *N*-(2,6-Diisopropylphenyl)-3-iodopyrrole in $CDCl_3$.

1.5 Tricyclohexyl-(N-(2,6-diisopropylphenyl)pyrrol)phosphonium iodide ***11***

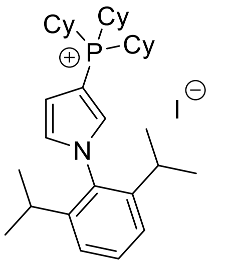

The compound was synthesized similar to a known procedure.[6]

2.46 g (879 mmol, 1.00 eq) tricyclohexylphosphine and 120.9 mg (439.5 µmol, 0.05 eq) bis(cycloocta-1,5-dien)nickel(0) were dissolved in 5 mL of ethanol. 3.11 g (8.79 mmol, 1.00 eq) *N*-(2,6-diisopropylphenyl)-3-iodopyrrol were dissolved in 25 mL ethanol and added to the solution. The mixture was then heated to reflux for 16 h, resulting in a color change to red. The solution was diluted with 100 mLwater and extracted with DCM (3 x 100 mL). The combined organic layers were dried over magnesium sulphate and all volatile compounds removed in vacuo. The resulting solid was purified by column chromatography (*n*-hexan/ethyl acetate: gradient). The crude product was again dissolved in dichloromethane and precipitated with diethyl ether. The product was obtained as a colorless solid in 61 % (3.40 g, 5.37 mmol) yield. Single crystals suitable for X-ray diffraction analysis were obtained by diffusion of diethyl ether into a solution of the product in dichloromethane.

Melting Point: 133.7 °C (dec.)

^{1}H NMR (400.3 MHz, RT, $CDCl_3$): δ = 1.14 (d, $^{3}J_{HH}$ = 6.9 Hz, 6 H, 2 CH_3), 1.18 (d, $^{3}J_{HH}$ = 6.9 Hz, 6 H, 2 CH_3), 1.23-1.33 (m, 3 H, 3 CH_2), 1.43-1.68 (m, 12 H, 12 CH_2), 1.79-1.88 (m, 3 H, 3 CH_2), 1.88-1.99 (m, 6 H, 6 CH_2), 2.05-2.18 (m, 6 H, 6 CH_2), 2.30 (hept, $^{3}J_{HH}$ = 6.9 Hz, 2 H, $CHMe_2$), 2.86-2.99 (m, 3 H, 3 PC*H*), 6.88-6.92 (m, 1 H, $NCH_{Pyr}CH_{Pyr}C_{q,Pyr}$), 6.94-6.98 (m, 1 H, $NCH_{Pyr}CH_{Pyr}C_{q,Pyr}$), 7.23-7.26 (m, 3 H, 2 *m*-CH_{Ar}, $NCH_{Pyr}C_q$), 7.45 (t, ^{3}J = 7.3 Hz, 1 H, *p*-CH_{Ar}).

$^{13}C\{^{1}H\}$ NMR (100.7 MHz, RT, $CDCl_3$): δ = 24.1, 24.9 (4 CH_3), 25.9 (3 CH_2), 26.6 (d, $^{2}J_{CP}$ = 12.3 Hz, 6 CH_2), 27.4 (d, $^{3}J_{CP}$ = 3.5 Hz, 6 CH_2), 28.7 ($CHMe_2$), 30.8 (d, $^{1}J_{CP}$ = 44.6 Hz, 3 PCH), 92.7 (d, $^{1}J_{CP}$ = 96.1 Hz, $C_{q,Pyr}$), 114.1 (d, $^{3}J_{CP}$ = 7.5 Hz, $NCH_{Pyr}CH_{Pyr}C_{q,Pyr}$), 124.2 (2 *m*-$CH_{Ar}$), 127.5 (d, $^{2}J_{CP}$ = 10.5 Hz,

NCH$_{Pyr}$CH$_{Pyr}$C$_{q, Pyr}$), 130.5 (*p*-CH$_{Ar}$), 131.1 (d, $^{2}J_{CP}$ = 14.9 Hz, NCH$_{Pyr}$C$_{q, Pyr}$), 135.0 (*i*-CH$_{q, Ar}$), 146.0 (2 *o*-C$_{q, Ar}$).
^{31}P{^{1}H} NMR (162.1 MHz, RT, CDCl$_3$): δ = 26.1.
IR (ATR): 3059 (w), 2929 (s), 2853 (m), 1504 (m), 1445 (m), 1363 (w), 1302 (w), 1268 (w), 1170 (s), 1110 (m), 1062 (m), 1006 (m), 922 (w), 898 (w), 851 (m), 805 (m), 758 (m), 728 (s), 697 (m), 660 (m), 636 (w), 565 (m), 555 (s), 534 (s), 523 (m), 496 (w), 461 (m), 441 (w).

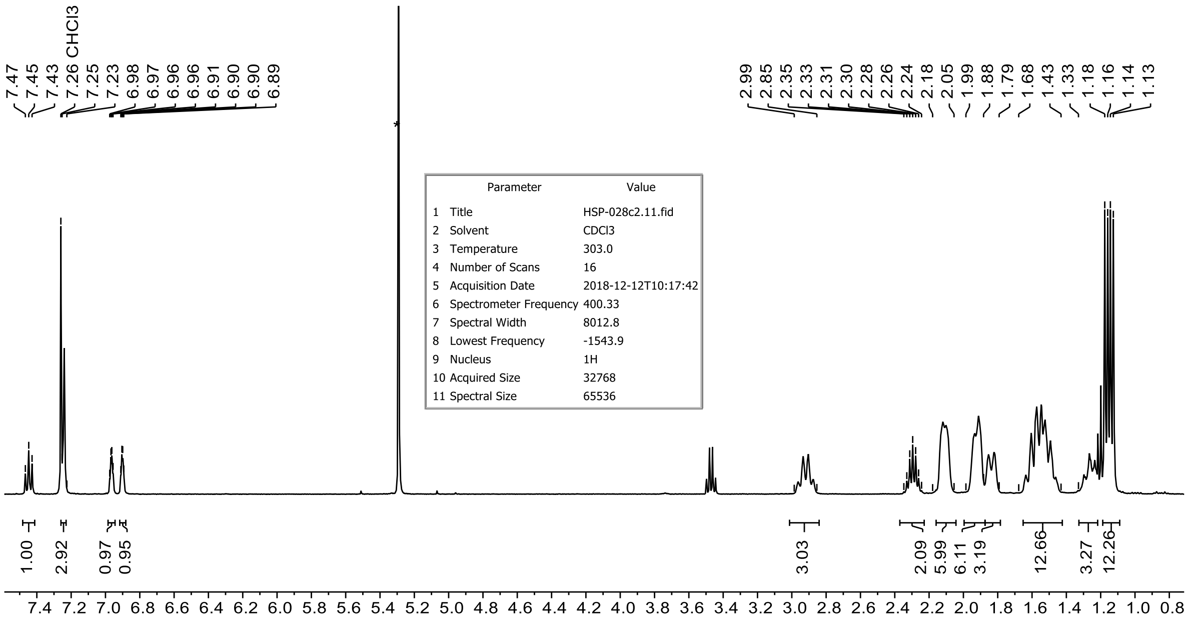

Figure 12. ^{1}H NMR spectrum of **11** in CDCl$_3$ (* = CH$_2$Cl$_2$).

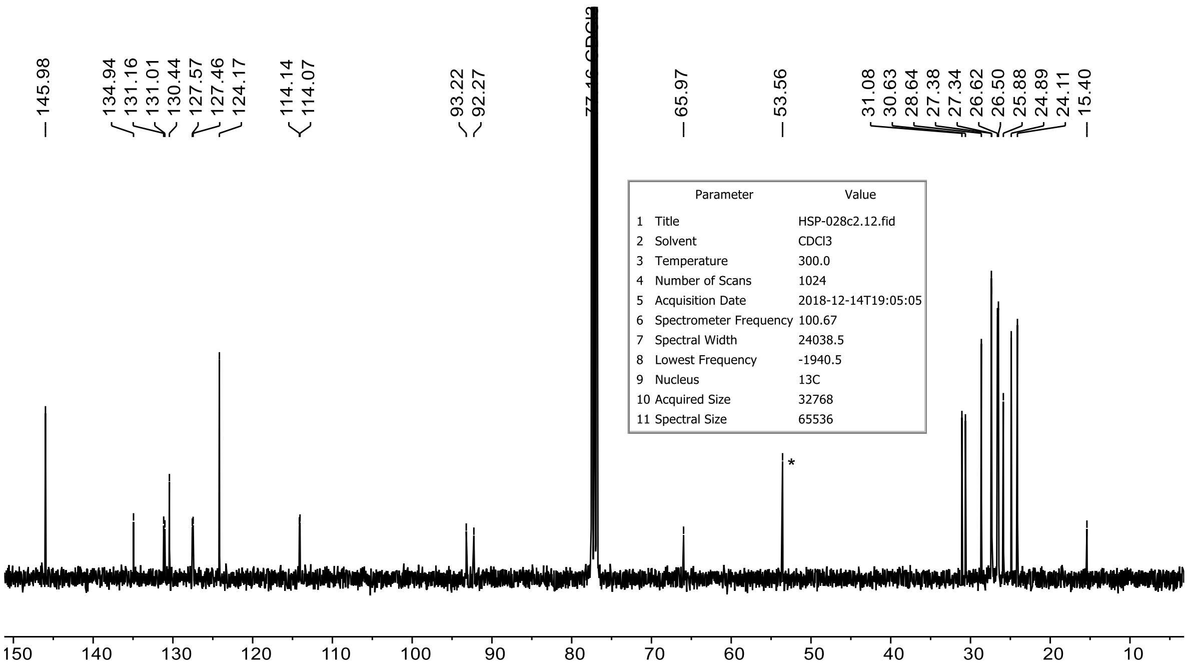

Figure 13. ^{13}C{^{1}H} NMR of11 in CDCl$_3$ (* = CH$_2$Cl$_2$).

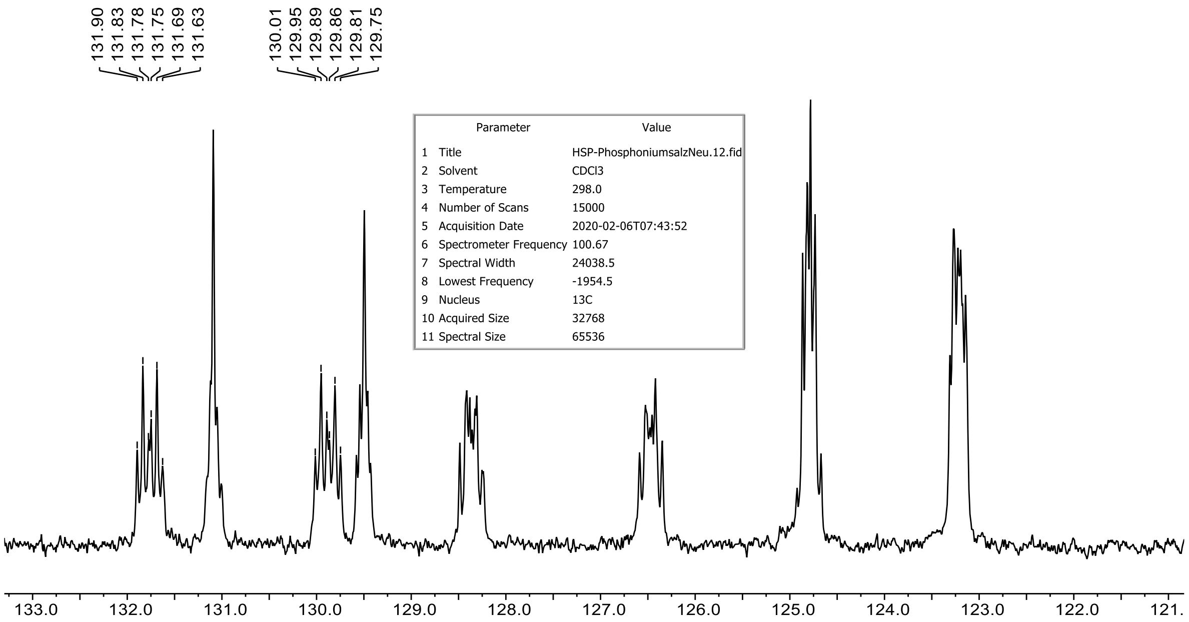

Figure 14. Section of the ^{13}C NMR spectrum of **11** in $CDCl_3$.

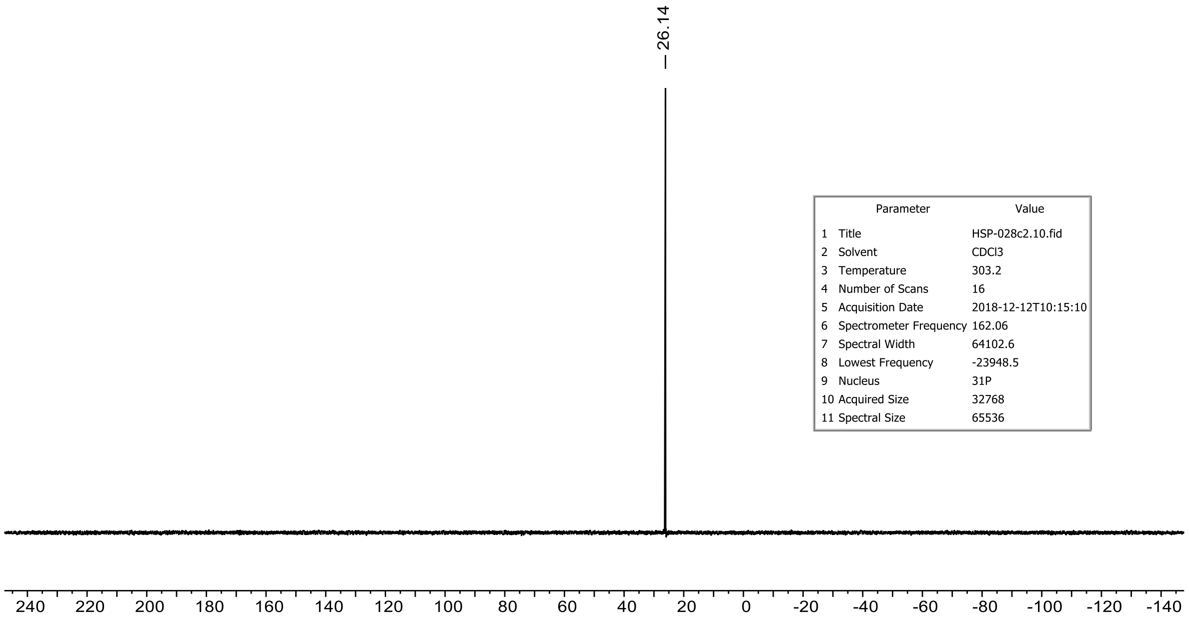

Figure 15. ^{31}P{^{1}H} NMR of **11** in $CDCl_3$.

1.6 Dipp-Pyrr-PCy$_2$=C$_6$H$_{10}$ **12**

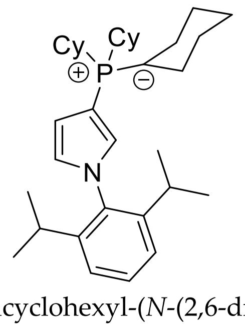

A) 1.00 g (1.58 mmol, 1.00 eq) tricyclohexyl-(*N*-(2,6-diisopropylphenyl)pyrrol)-phosphonium iodide **11** was suspended in 30 mL of toluene and cooled to -100 °C. After addition of 1.72 mL (34.68 mg, 1.58 mmol, 1.0 eq) of a 0.92 M solution of methyllithium in diethylether, the mixture was

allowed to warm to room temperature and stirred for 16 hours. After filtration all volatiles were removed *in vacuo*. The product **11** was obtained as yellow solid in 60 % (480.5 mg, 950.0 µmol) yield. The synthesis can also be performed in tetrahydrofuran. In this case the solvent must be exchanged to toluene before filtration. Single crystals suitable for X-ray diffraction analysis were obtained by diffusion of *n*-hexane in a solution of **12** in THF.

A) 126.74 g (200.0 µmol, 1.00 eq) tricyclohexyl-(*N*-(2,6-diisopropylphenyl)pyrrol)phosphonium iodide 1**1** was suspended in 2.5 mL of toluene and cooled to -70 °C. After addition of 0.1 mL (21.43 mg, 200.0 µmol, 1.0 eq) of a 2.0 M solution of lithium diisopropylamide in tetrahydrofurane/heptane/ethylbenzene, the mixture was allowed to warm to room temperature and stirred for 16 hours. Volatile compounds were removed *in vacuo* and the residue was dissolved in toluene. After filtration all volatiles were removed *in vacuo*. The crude product **11** was obtained as orange solid in 60 % (60.0 mg, 118.6 µmol) yield, containing 18 % of **11**.

Melting Point: 145.9 °C.
^{1}H NMR (400.3 MHz, RT, C_6D_6): δ = 1.05 (d, ^{3}J = 6.9 Hz, 6 H, 2 CH_3), 1.08 (d, ^{3}J = 6.9 Hz, 6 H, 2 CH_3), 1.14-1.41 (m, 9 H, 9 CH_2), 1.60-1.88 (m, 12 H, 12 CH_2), 1.89-1.99 (m, 4 H, 2 CH_2), 2.11-2.20 (m, 2 H, 2 CH_2), 2.53-2.64 (m, 5 H, 2 PCH, 3 CH_2), 6.43 (dd, $^{3}J_{HH}$ = 2.4 Hz, $^{4}J_{HH}$ = 2.4 Hz, 1 H, CH_{Pyr}), 6.57 (dd, $^{3}J_{HH}$ = 2.4 Hz, $^{4}J_{HH}$ = 2.4 Hz, 1 H, CH_{Pyr}), 7.00-7.06 (m, 3 H, CH_{Pyr}, 2 *m*-CH_{Ar}), 7.20 (t, $^{3}J_{HH}$ = 7.7 Hz, 1 H, *p*-CH_{Ar}).
^{13}C{^{1}H} NMR (100.7 MHz, RT, C_6D_6): δ = 12.0 (d, $^{1}J_{CP}$ = 125.0 Hz, PC_q(CH_2)$_2$), 24.2 (2 CH_3), 24.9 (2 CH_3), 26.2 (d, ^{3}J = 0.8 Hz, 2 CH_2), 27.0 (d, ^{3}J = 1.0 Hz, 2 CH_2), 27.2 (d, $^{3}J_{CP}$ = 1.4 Hz, 2 CH_2), 27.2 (d, $^{2}J_{CP}$ = 23.4 Hz, 2 CH_2), 27.3 (2 CH_2), 28.6 (2 CH(CH_3)$_2$), 29.6 (CH_2) 31.8 (d, ^{2}J = 8.2 Hz, 2 CH_2), 31.9 (d, ^{2}J = 10.6 Hz, 2 CH_2), 34.0 (d, $^{1}J_{CP}$ = 20.2 Hz, 2 PCH), 108.3 (d, $^{1}J_{CP}$ = 104.6, $C_{q, Pyr}$), 115.1 (d, $^{3}J_{CP}$ = 7.6 Hz, N$CH_{Pyr}CH_{Pyr}C_{q, Pyr}$), 123.9 (2 *m*-$CH_{Ar}$), 124.2 (d, $^{2}J_{CP}$ = 9.5 Hz, N$CH_{Pyr}CH_{Pyr}C_{q, Pyr}$), 129.6 (*p*-$CH_{Ar}$), 132.3 (d, $^{2}J_{CP}$ = 13.5 Hz, N$CH_{Pyr}C_{q, Pyr}$), 137.1 (*i*-$C_{q, Ar}$), 146.8 (2 *o*-$C_{q, Ar}$).
^{31}P{^{1}H} NMR (162.06 MHz, RT, C_6D_6): δ = 6.6.
IR(ATR): 2963 (m), 2926 (s), 2856 (m), 2725 (m), 1503 (m), 1488 (m), 1451 (m), 1383 (w), 1361 (m), 1346 (m), 1324 (m), 1300 (w), 1260 (m), 1188 (m), 1176 (m), 1159 (m), 1102 (m), 1076 (m), 1053 (vs), 1016 (m), 999 (m), 941 (m), 849 (w), 841 (w), 805 (vs), 760 (m), 732 (m), 709 (m), 695 (w), 655 (m), 635 (w), 622 (w), 564 (m), 548 (s), 534 (s), 526 (m), 490 (w), 462 (m), 442 (w), 426 (w).

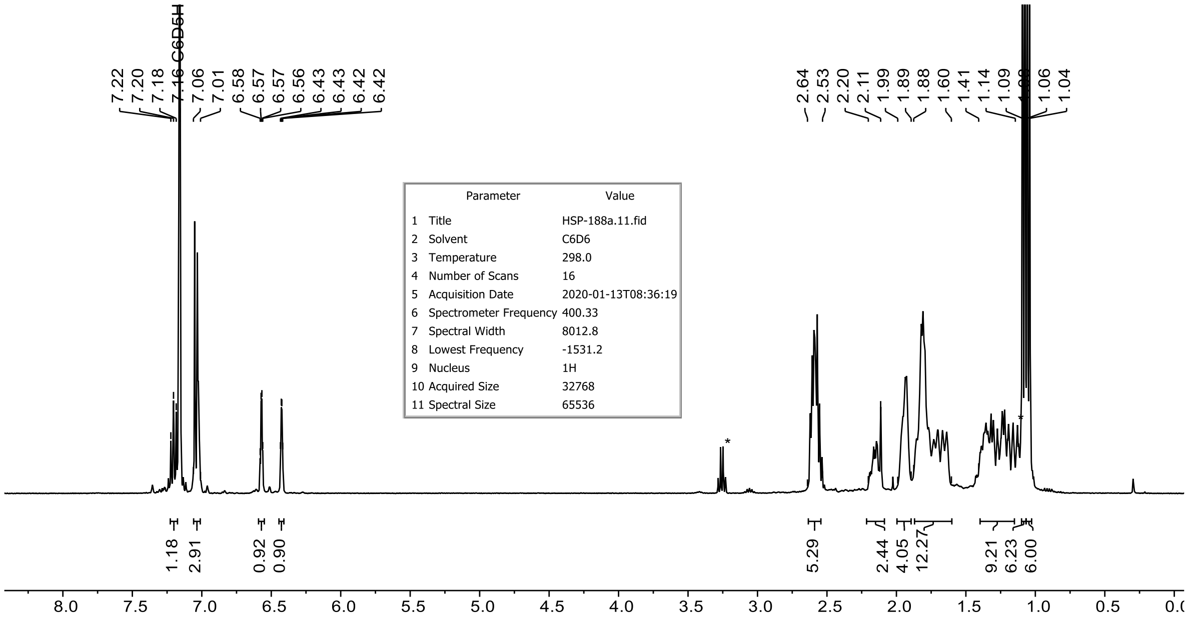

Figure 16. ^{1}H NMR spectrum of **12** in C_6D_6 (* = Et_2O).

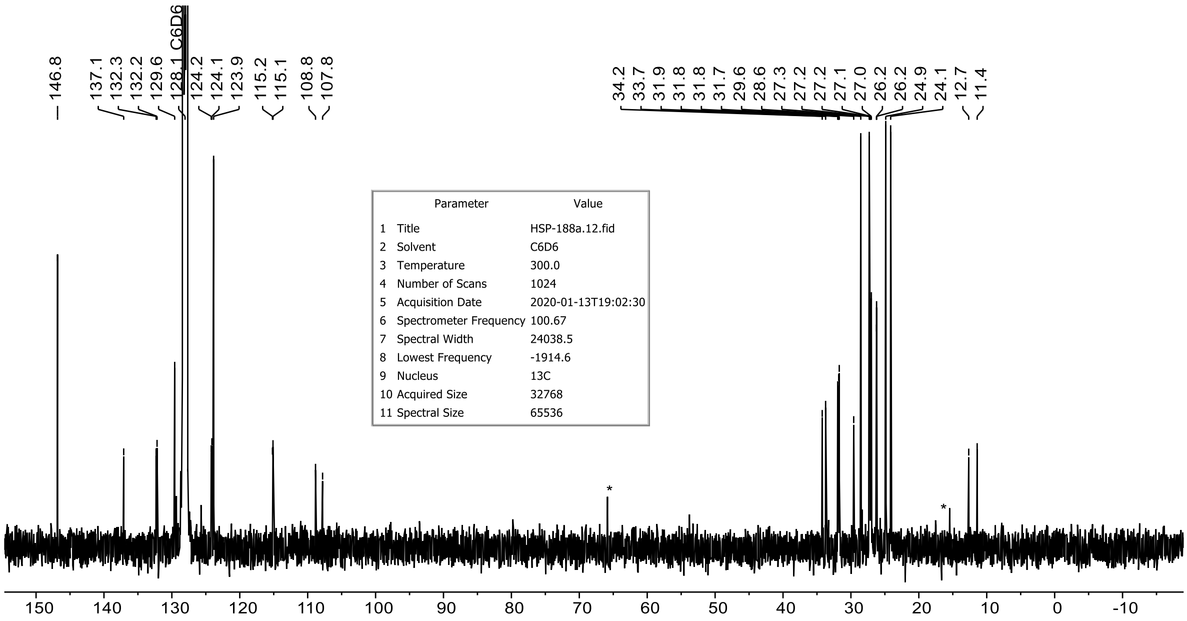

Figure 17. $^{13}C\{^{1}H\}$ NMR spectrum of **12** in C_6D_6 (* = Et_2O).

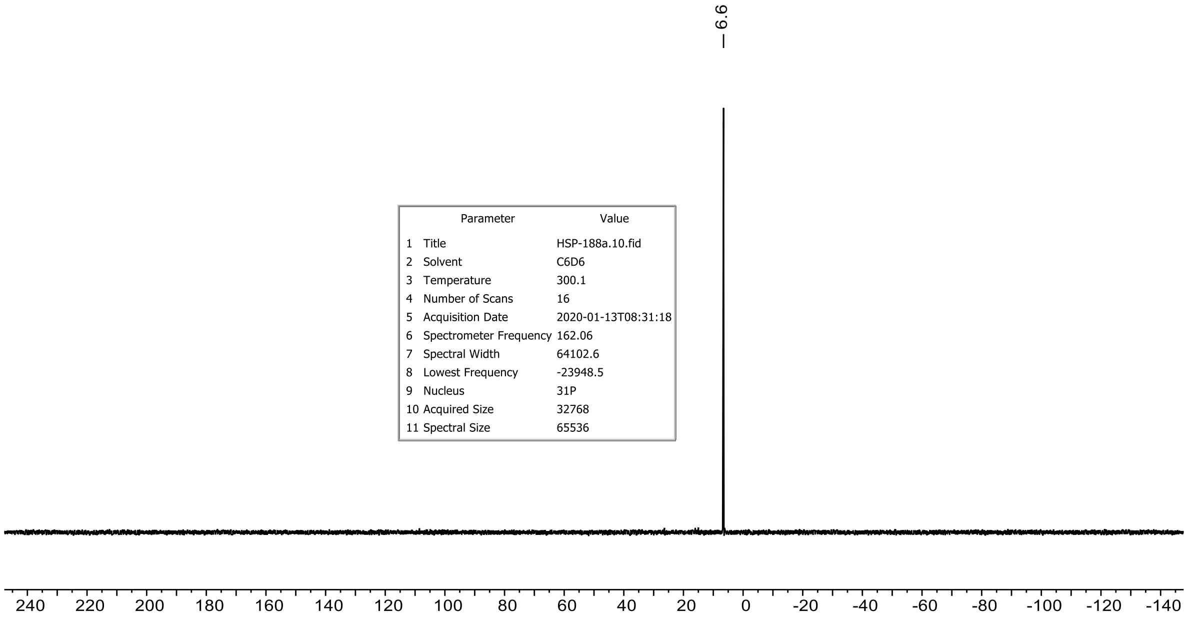

Figure 18. $^{31}P\{^{1}H\}$ NMR spectrum of **12** in C_6D_6.

2. Crystal Structure Determination

All data were collected with an OXFORD *SuperNova* diffractometer. The structures were solved using direct methods. The *Shelx* software package [7–9] was used for refinement and expansion was carried out using FOURIER techniques. An inert oil (perfluoropolyalkylether) was used to mount the crystals and the crystal structure determination was performed at 100 K using Mo-K_{α} radiation. The corresponding data have been deposited with the CAMBRIDGE CRYSTALLOGRAPHIC DATA CENTRE as supplementary publication no. CCDC-1979639-1979642. Copies of the data can be obtained free of charge on application to CAMBRIDGE CRYSTALLOGRAPHIC DATA CENTRE, 12 Union Road, Cambridge CB2 1EZ; UK [Homepage: https://www.ccdc.cam.ac.uk/].

Diamond 4.0 [10] by CRYSTAL IMPACT and *GIMP* 2.10 [11] were used for graphic representation, Ellipsoids are drawn at the 50 % probability level.

The structure of **9** contained a highly disordered solvent molecule that was treated by using the PLATON/SQUEZZE routine. [12]

2.1 Crystal data and structure refinements

Compound	*N*-(2,6-diisopropyl-phenyl)pyrrole	**6**
CCDC No	1979639	1979642
Empirical formula	$C_{16}H_{21}N$	$C_{18}H_{26}BF_4NS$
Formula weight [g/mol]	227.34	375.27
Temperature [K]	100(2)	100(2)
Wavelength [Å]	1.54184	1.54184
Crystal system	orthorhombic	monoclinic
Space group	$P2_12_12_1$	$P2_1/c$
Unit cell dimensions	a = 8.35296(13) Å	a = 21.2321(3) Å
	b = 11.27608(18) Å	b = 8.17102(9) Å
	c = 14.6485(2) Å	c = 11.51387(15) Å
	α = 90°	α = 90°
	β =90°	β = 104.7271(14)°
	γ = 90°	γ = 90°
Volume [Å^3]	1379.73(4)	1931.89(4)
Z	4	4
Density (calculated) [Mg/m^3]	1.094	1.290
Absorption coefficient [mm^{-1}]	0.471	1.824
F(000)	496	792
Crystal size [mm^3]	0.232 x 0.129 x 0.036	0.350 x 0.167 x 0.027
Theta range [°]	4.949 to 74.851	4.306 to 74.987
Index ranges	-10<=h<=9<br>-14<=k<=13<br>-18<=l<=18	-26<=h<=26<br>-8<=k<=10<br>-14<=l<=14
Reflections collected	9012	28827
Independent reflections	2817 [R_{int} = 0.0308]	3980 [R_{int} = 0.0428]
Data / restraints / parameters	2817 / 0 / 158	3980 / 0 / 232
Goodness-of-fit on F2	1.061	1.065
Final R indices [I>2sigma(I)]	R1 = 0.0348, wR2 = 0.0900	R1 = 0.0386, wR2 = 0.1098
R indices (all data)	R1 = 0.0357, wR2 = 0.0906	R1 = 0.0403, wR2 = 0.1116
Largest diff. peak and hole	0.203 and -0.187	0.594 and -0.240

Compound	**9**	**11**

CCDC No	1979641	1979640
Empirical formula	$C_{35}H_{55}Cl_2INP$	$C_{34}H_{52}NP$
Formula weight [g/mol]	718.57	2022.93
Temperature [K]	100(2)	100(2)
Wavelength [Å]	1.54184	1.54184
Crystal system	monoclinic	Monoclinic
Space group	*P*2₁/c	*P*2₁/c
Unit cell dimensions	a = 11.74361(11) Å	a = 9.82504(11) Å
	b = 19.74256(17) Å	b = 15.61527(17) Å
	c = 19.4546(2) Å	c = 20.0819(2) Å
	α = 90°	α= 90°
	β = 106.2513(10)°	β= 102.8877(10)°
	γ = 90°	γ = 90°
Volume [Å^3]	4330.30(7)	3003.36(6)
Z	4	1
Density (calculated) [Mg/m^3]	1.102	1.118
Absorption coefficient [mm^{-1}]	7.428	0.951
F(000)	1496	1112
Crystal size [mm^3]	0.338 x 0.328 x 0.116	0.226 x 0.138 x 0.054
Theta range [°]	3.257 to 74.995°	3.621 to 77.464
Index ranges	-14<=h<=14<br>-21<=k<=24<br>-24<=l<=20	-12<=h<=9<br>-19<=k<=19<br>-25<=l<=25
Reflections collected	36183	24513
Independent reflections	8849 [R(int) = 0.0492]	6176 [R(int) = 0.0409]
Data / restraints / parameters	8849 / 0 / 365	6176 / 0 / 329
Goodness-of-fit on F^2	1.02	1.06
Final R indices [I>2sigma(I)]	R1 = 0.0441, wR2 = 0.1187	R1 = 0.0408, wR2 = 0.1053
R indices (all data)	R1 = 0.0447, wR2 = 0.1192	R1 = 0.0458, wR2 = 0.1089
Largest diff. peak and hole	1.426 and -1.251	0.324 and -0.401

2.2 N-(2,6-diisopropylphenyl)pyrrole

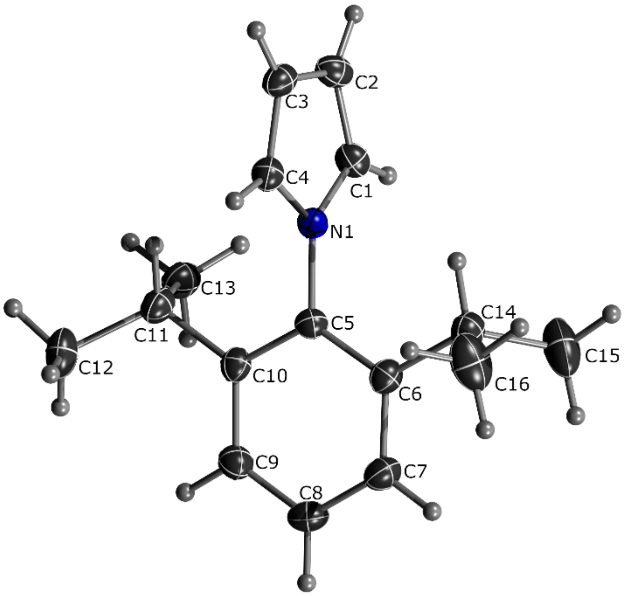

Figure 19. ORTEP plot of *N*-(2,6-Diisopropylphenyl)pyrrole

Table 1. Atomic coordinates (x 10^4) and equivalent isotropic displacement parameters (Å^{2}x 10^3) for *N*-(2,6-diisopropylphenyl)pyrrole. U(eq) is defined as one third of the trace of the orthogonalized U^{ij} tensor.

	x	y	z	U(eq)
N(1)	4184(2)	3947(1)	5328(1)	21(1)
C(1)	5525(2)	4584(2)	5096(1)	24(1)
C(2)	6128(2)	4125(2)	4302(1)	27(1)
C(3)	5128(2)	3168(2)	4044(1)	26(1)
C(4)	3941(2)	3080(2)	4683(1)	23(1)
C(5)	3251(2)	4105(1)	6144(1)	21(1)
C(6)	1978(2)	4919(2)	6135(1)	24(1)
C(7)	1086(2)	5047(2)	6934(1)	28(1)
C(8)	1442(2)	4390(2)	7707(1)	29(1)
C(9)	2714(2)	3598(2)	7698(1)	26(1)
C(10)	3649(2)	3440(1)	6919(1)	22(1)
C(11)	5073(2)	2601(2)	6925(1)	27(1)
C(12)	4780(3)	1493(2)	7497(1)	38(1)
C(13)	6595(2)	3244(2)	7254(1)	32(1)
C(14)	1565(2)	5641(2)	5289(1)	28(1)
C(15)	1717(4)	6972(2)	5451(2)	49(1)
C(16)	-91(3)	5324(2)	4947(2)	46(1)

Table 2. Anisotropic displacement parameters (Å^{2}x 10^3) for *N*-(2,6-diisopropylphenyl)pyrrole. The anisotropic displacement factor exponent takes the form: -2π [h^2 $a^{*2}U^{11}$ + ... + 2 h k a* b* U^{12}]

	U_{11}	U_{22}	U_{33}	U_{23}	U_{13}	U_{12}
N(1)	22(1)	20(1)	20(1)	0(1)	0(1)	2(1)
C(1)	21(1)	26(1)	25(1)	3(1)	-2(1)	-2(1)
C(2)	21(1)	36(1)	24(1)	5(1)	1(1)	2(1)
C(3)	28(1)	29(1)	21(1)	-2(1)	-1(1)	8(1)

C(4)	26(1)	22(1)	22(1)	-1(1)	-3(1)	1(1)
C(5)	20(1)	22(1)	19(1)	-1(1)	-1(1)	0(1)
C(6)	22(1)	24(1)	24(1)	-1(1)	-3(1)	2(1)
C(7)	26(1)	31(1)	28(1)	-3(1)	2(1)	6(1)
C(8)	28(1)	34(1)	24(1)	-4(1)	6(1)	1(1)
C(9)	32(1)	26(1)	20(1)	0(1)	-1(1)	0(1)
C(10)	25(1)	20(1)	22(1)	-2(1)	-3(1)	1(1)
C(11)	32(1)	28(1)	23(1)	-1(1)	-2(1)	9(1)
C(12)	45(1)	26(1)	42(1)	4(1)	-11(1)	6(1)
C(13)	28(1)	37(1)	30(1)	1(1)	0(1)	8(1)
C(14)	29(1)	27(1)	26(1)	1(1)	-2(1)	4(1)
C(15)	79(2)	28(1)	38(1)	5(1)	-18(1)	-9(1)
C(16)	43(1)	46(1)	50(1)	14(1)	-21(1)	-8(1)

2.3 [Dipp-Pyrr-SMe$_2$][BF]$_4$ ***9***

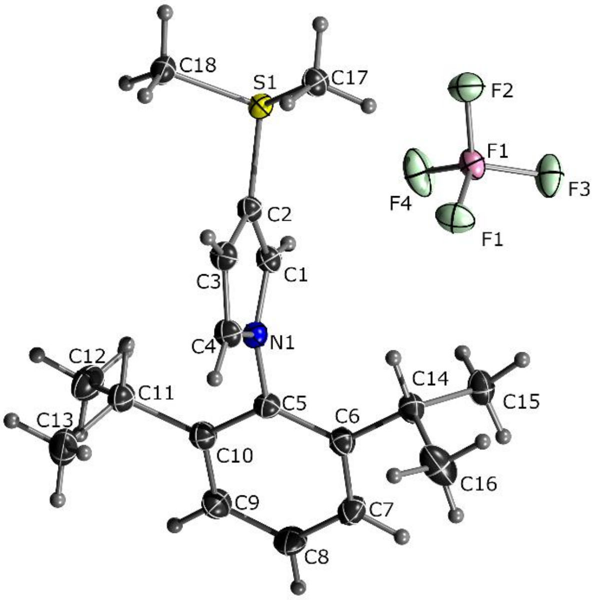

Figure 20. ORTEP plot of **9**.

Table 3. Atomic coordinates (x 10^4) and equivalent isotropic displacement parameters (Å^{2}x 10^3) for **9**. U(eq) is defined as one third of the trace of the orthogonalized U^{ij} tensor.

	x	y	z	U(eq)
S(1)	4054(1)	3063(1)	4361(1)	19(1)
F(1)	6051(1)	3666(1)	7996(1)	33(1)
F(2)	5225(1)	3332(1)	6340(1)	34(1)
F(3)	5740(1)	1097(1)	7300(1)	35(1)
F(4)	6259(1)	2750(1)	6259(1)	41(1)
N(1)	2390(1)	3744(1)	2056(1)	19(1)
B(1)	5825(1)	2714(2)	6986(1)	22(1)
C(1)	2867(1)	4160(2)	3048(1)	20(1)
C(2)	3341(1)	2974(1)	3199(1)	19(1)
C(3)	3151(1)	1803(2)	2263(1)	22(1)

C(4)	2560(1)	2318(2)	1575(1)	22(1)
C(5)	1800(1)	4660(2)	1581(1)	20(1)
C(6)	1808(1)	5911(2)	752(1)	22(1)
C(7)	1226(1)	6767(2)	301(1)	27(1)
C(8)	673(1)	6408(2)	673(1)	30(1)
C(9)	680(1)	5162(2)	1498(1)	28(1)
C(10)	1243(1)	4255(2)	1967(1)	23(1)
C(11)	1238(1)	2853(2)	2833(1)	26(1)
C(12)	956(1)	3360(2)	3876(1)	35(1)
C(13)	867(1)	1378(2)	2179(1)	37(1)
C(14)	2414(1)	6359(2)	354(1)	23(1)
C(15)	2608(1)	8137(2)	660(1)	28(1)
C(16)	2326(1)	6050(2)	-986(2)	40(1)
C(17)	4682(1)	2763(2)	3592(1)	23(1)
C(18)	4054(1)	1123(2)	5100(1)	24(1)

Table 4. Anisotropic displacement parameters (Å^{2}x 10^3) for **9**. The anisotropic displacement factor exponent takes the form: $-2\pi^2$[h^2 $a^{*2}U^{11}$ + ... + 2 h k a* b* U^{12}]

	U_{11}	U_{22}	U_{33}	U_{23}	U_{13}	U_{12}
S(1)	19(1)	18(1)	19(1)	0(1)	5(1)	0(1)
F(1)	33(1)	34(1)	29(1)	-11(1)	2(1)	-1(1)
F(2)	32(1)	34(1)	30(1)	-1(1)	-1(1)	2(1)
F(3)	42(1)	20(1)	48(1)	2(1)	22(1)	2(1)
F(4)	44(1)	37(1)	52(1)	-12(1)	32(1)	-11(1)
N(1)	19(1)	17(1)	21(1)	0(1)	5(1)	1(1)
B(1)	23(1)	19(1)	26(1)	-4(1)	9(1)	-1(1)
C(1)	21(1)	19(1)	21(1)	-2(1)	6(1)	-1(1)
C(2)	19(1)	20(1)	20(1)	1(1)	6(1)	0(1)
C(3)	23(1)	18(1)	25(1)	-2(1)	5(1)	2(1)
C(4)	24(1)	19(1)	23(1)	-3(1)	5(1)	0(1)
C(5)	19(1)	19(1)	21(1)	-1(1)	4(1)	1(1)
C(6)	22(1)	19(1)	24(1)	0(1)	7(1)	0(1)
C(7)	27(1)	23(1)	30(1)	7(1)	7(1)	4(1)
C(8)	22(1)	32(1)	35(1)	6(1)	6(1)	7(1)
C(9)	22(1)	34(1)	31(1)	5(1)	10(1)	3(1)
C(10)	23(1)	24(1)	23(1)	1(1)	6(1)	0(1)
C(11)	23(1)	29(1)	26(1)	6(1)	8(1)	1(1)
C(12)	41(1)	40(1)	27(1)	6(1)	14(1)	2(1)
C(13)	48(1)	32(1)	31(1)	4(1)	11(1)	-9(1)
C(14)	23(1)	20(1)	28(1)	1(1)	9(1)	-1(1)
C(15)	33(1)	23(1)	30(1)	-3(1)	12(1)	-6(1)
C(16)	39(1)	49(1)	37(1)	-19(1)	20(1)	-16(1)
C(17)	21(1)	26(1)	24(1)	1(1)	9(1)	0(1)
C(18)	28(1)	22(1)	23(1)	4(1)	7(1)	-1(1)

2.4 [Dipp-Pyrr-PCy₃]I ***11***

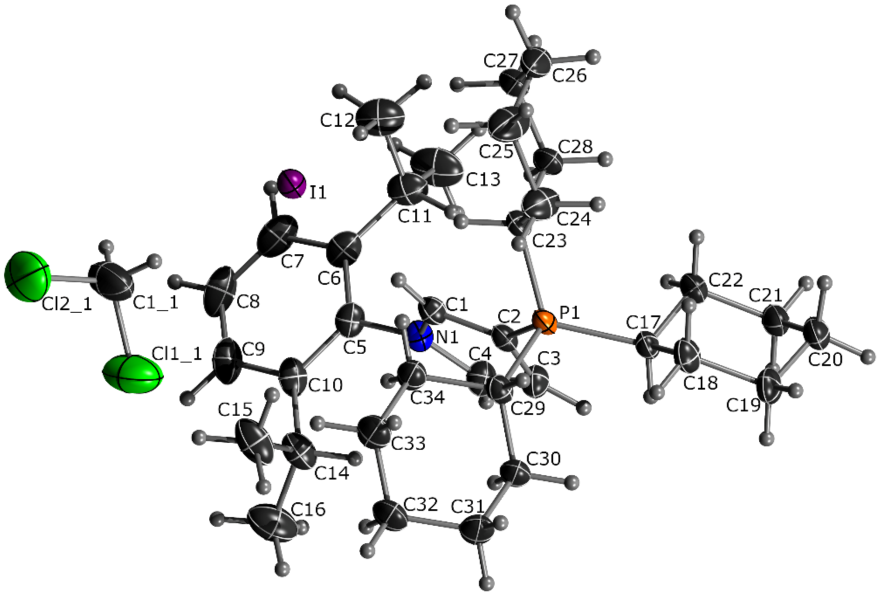

Figure 21. ORTEP plot of **11**.

Table 5. Atomic coordinates(x 10^4) and equivalent isotropic displacement parameters (Å^{2}x 10^3) for **11** U(eq) is defined as one third of the trace of the orthogonalized U^{ij} tensor.

	x	y	z	U(eq)
I(1)	3345(1)	7289(1)	5107(1)	27(1)
P(1)	2412(1)	7927(1)	2524(1)	22(1)
N(1)	3719(2)	6099(1)	2928(1)	25(1)
C(1)	3334(2)	6697(1)	3127(2)	24(1)
C(2)	2943(2)	7091(2)	2517(2)	23(1)
C(3)	3106(3)	6711(2)	1928(2)	30(1)
C(4)	3574(3)	6101(2)	2203(2)	31(1)
C(5)	4196(3)	5549(1)	3407(2)	26(1)
C(6)	3411(3)	5107(2)	3600(2)	32(1)
C(7)	3899(4)	4574(2)	4057(2)	42(1)
C(8)	5117(4)	4489(2)	4303(2)	45(1)
C(9)	5871(4)	4938(2)	4107(2)	40(1)
C(10)	5430(3)	5485(2)	3655(2)	31(1)
C(11)	2076(3)	5200(2)	3325(2)	38(1)
C(12)	1545(4)	5392(2)	3930(3)	53(1)
C(13)	1484(4)	4565(2)	2931(3)	58(1)
C(14)	6253(3)	6012(2)	3476(2)	37(1)
C(15)	6636(3)	6518(2)	4088(2)	48(1)
C(16)	7331(4)	5701(2)	3302(3)	57(1)
C(17)	1647(2)	8154(2)	1600(2)	25(1)
C(18)	1422(3)	8921(2)	1478(2)	31(1)
C(19)	951(3)	9059(2)	674(2)	35(1)
C(20)	-167(3)	8646(2)	331(2)	35(1)
C(21)	46(3)	7890(2)	480(2)	33(1)
C(22)	497(3)	7751(2)	1285(2)	29(1)

C(23)	1544(2)	7944(2)	3167(2)	25(1)
C(24)	1146(4)	8658(2)	3309(2)	42(1)
C(25)	549(4)	8638(2)	3916(2)	49(1)
C(26)	-476(3)	8134(3)	3753(2)	52(1)
C(27)	-68(3)	7428(2)	3610(2)	41(1)
C(28)	495(3)	7435(2)	2990(2)	37(1)
C(29)	3683(2)	8494(2)	2842(2)	26(1)
C(30)	4489(3)	8469(2)	2344(2)	33(1)
C(31)	5558(3)	8943(2)	2615(2)	44(1)
C(32)	6263(3)	8763(2)	3375(2)	42(1)
C(33)	5470(3)	8783(2)	3872(2)	40(1)
C(34)	4399(3)	8315(2)	3612(2)	32(1)
Cl11	7152(1)	7393(1)	5754(1)	86(1)
Cl21	7119(1)	7680(1)	7188(1)	86(1)
C11	6308(5)	7445(4)	6350(3)	73(2)

Table 6. Anisotropic displacement parameters (Å^{2}x 10^3) for **11**. The anisotropic displacement factor exponent takes the form: $-2\pi^2[h^2 a^{*2}U^{11} + ... + 2 h k a^* b^* U^{12}]$

	U_{11}	U_{22}	U_{33}	U_{23}	U_{13}	U_{12}
I(1)	24(1)	27(1)	30(1)	1(1)	5(1)	0(1)
P(1)	15(1)	21(1)	28(1)	4(1)	3(1)	1(1)
N(1)	25(1)	19(1)	28(1)	0(1)	4(1)	0(1)
C(1)	22(1)	21(1)	28(1)	-2(1)	5(1)	-1(1)
C(2)	16(1)	21(1)	30(1)	1(1)	3(1)	0(1)
C(3)	31(2)	31(2)	26(1)	2(1)	5(1)	5(1)
C(4)	36(2)	27(2)	29(2)	-1(1)	7(1)	3(1)
C(5)	34(2)	16(1)	27(1)	-2(1)	5(1)	-1(1)
C(6)	46(2)	20(1)	31(2)	-5(1)	12(1)	-6(1)
C(7)	69(3)	20(1)	38(2)	-2(1)	18(2)	-5(2)
C(8)	76(3)	21(2)	32(2)	2(1)	7(2)	13(2)
C(9)	51(2)	28(2)	35(2)	-1(1)	1(2)	14(2)
C(10)	35(2)	20(1)	32(2)	-2(1)	1(1)	5(1)
C(11)	42(2)	30(2)	44(2)	-7(1)	15(2)	-14(1)
C(12)	58(2)	46(2)	62(3)	-11(2)	29(2)	-10(2)
C(13)	53(2)	48(2)	76(3)	-26(2)	24(2)	-24(2)
C(14)	28(2)	30(2)	49(2)	-1(1)	3(1)	1(1)
C(15)	36(2)	32(2)	67(3)	-12(2)	-4(2)	-1(1)
C(16)	43(2)	44(2)	88(3)	-4(2)	25(2)	3(2)
C(17)	18(1)	28(1)	26(1)	4(1)	2(1)	2(1)
C(18)	28(2)	26(2)	34(2)	6(1)	2(1)	4(1)
C(19)	31(2)	35(2)	33(2)	11(1)	1(1)	6(1)
C(20)	25(2)	48(2)	28(2)	3(1)	2(1)	8(1)
C(21)	24(2)	46(2)	26(2)	-1(1)	2(1)	0(1)
C(22)	21(1)	34(2)	28(2)	0(1)	2(1)	-1(1)
C(23)	17(1)	29(2)	28(1)	5(1)	4(1)	3(1)
C(24)	47(2)	35(2)	51(2)	12(2)	26(2)	18(2)
C(25)	57(2)	44(2)	57(2)	12(2)	33(2)	24(2)

C(26)	32(2)	84(3)	43(2)	25(2)	18(2)	26(2)
C(27)	21(2)	67(2)	34(2)	9(2)	5(1)	-5(2)
C(28)	21(1)	58(2)	30(2)	3(2)	4(1)	-12(1)
C(29)	21(1)	24(1)	30(2)	3(1)	4(1)	-2(1)
C(30)	23(1)	44(2)	32(2)	5(1)	6(1)	-9(1)
C(31)	31(2)	58(2)	41(2)	12(2)	7(1)	-19(2)
C(32)	24(2)	55(2)	44(2)	1(2)	4(1)	-15(2)
C(33)	33(2)	45(2)	37(2)	-2(2)	3(1)	-19(2)
C(34)	28(2)	36(2)	28(2)	3(1)	3(1)	-11(1)
Cl11	68(1)	112(1)	91(1)	-25(1)	45(1)	-16(1)
Cl21	69(1)	118(1)	61(1)	-13(1)	0(1)	-36(1)
C11	39(2)	119(5)	58(3)	-22(3)	11(2)	-16(3)

2.5 Dipp-Pyrr-PCy$_2$=C$_6$H$_{10}$ ***12***

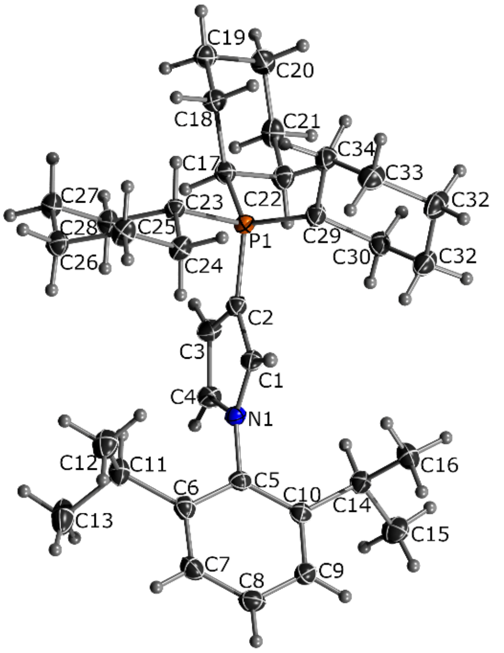

Figure 22. ORTEP plot of **12**.

Table 7. Atomic coordinates (x 10^4) and equivalent isotropic displacement parameters (Å^{2}x 10^3) for **12**. U(eq) is defined as one third of the trace of the orthogonalized U^{ij} tensor.

	x	y	z	U(eq)
P(1)	4621(1)	3762(1)	1841(1)	17(1)
N(1)	7601(1)	3818(1)	3523(1)	18(1)
C(1)	7075(1)	3653(1)	2847(1)	19(1)
C(2)	5660(1)	3818(1)	2694(1)	19(1)
C(4)	6533(1)	4090(1)	3813(1)	22(1)
C(3)	5325(1)	4100(1)	3316(1)	22(1)
C(27)	4988(1)	6350(1)	1340(1)	24(1)
C(7)	11300(2)	4454(1)	4116(1)	27(1)
C(6)	9865(1)	4525(1)	3868(1)	22(1)
C(5)	9067(1)	3776(1)	3835(1)	19(1)

C(9)	11093(1)	2943(1)	4306(1)	24(1)
C(8)	11916(2)	3672(1)	4329(1)	28(1)
C(23)	4976(1)	4733(1)	1383(1)	18(1)
C(24)	6444(1)	4722(1)	1234(1)	22(1)
C(28)	4761(1)	5564(1)	1758(1)	22(1)
C(29)	5010(1)	2881(1)	1443(1)	22(1)
C(17)	2815(1)	3940(1)	1967(1)	21(1)
C(22)	2361(1)	3133(1)	2302(1)	23(1)
C(18)	1796(1)	4110(1)	1277(1)	22(1)
C(26)	6436(1)	6342(1)	1184(1)	26(1)
C(25)	6686(2)	5512(1)	827(1)	26(1)
C(21)	852(2)	3204(1)	2377(1)	26(1)
C(19)	291(1)	4173(1)	1356(1)	24(1)
C(20)	-138(1)	3370(1)	1688(1)	26(1)
C(10)	9655(1)	2980(1)	4056(1)	20(1)
C(11)	9204(2)	5384(1)	3627(1)	26(1)
C(12)	9544(2)	5645(1)	2949(1)	34(1)
C(34)	4588(2)	2807(1)	671(1)	25(1)
C(33)	5651(2)	2276(1)	394(1)	29(1)
C(32)	5868(2)	1402(1)	744(1)	33(1)
C(31)	6321(2)	1494(1)	1521(1)	30(1)
C(30)	5256(2)	2020(1)	1798(1)	24(1)
C(13)	9674(2)	6084(1)	4164(1)	43(1)
C(14)	8771(1)	2173(1)	4005(1)	22(1)
C(15)	9074(2)	1662(1)	4670(1)	32(1)
C(16)	8989(2)	1616(1)	3410(1)	28(1)

Table 8. Anisotropic displacement parameters (Å^{2}x 10^3) for **12**. The anisotropic displacement factor exponent takes the form: $-2\pi^2[h^2 a^{*2}U^{11} + ... + 2 h k a^* b^* U^{12}]$

	U_{11}	U_{22}	U_{33}	U_{23}	U_{13}	U_{12}
P(1)	18(1)	17(1)	16(1)	1(1)	3(1)	1(1)
N(1)	19(1)	20(1)	16(1)	1(1)	2(1)	2(1)
C(1)	22(1)	20(1)	15(1)	1(1)	4(1)	1(1)
C(2)	21(1)	18(1)	18(1)	2(1)	4(1)	1(1)
C(4)	24(1)	25(1)	16(1)	-1(1)	6(1)	3(1)
C(3)	21(1)	27(1)	21(1)	0(1)	6(1)	3(1)
C(27)	27(1)	18(1)	28(1)	2(1)	7(1)	1(1)
C(7)	26(1)	24(1)	29(1)	-4(1)	4(1)	-7(1)
C(6)	28(1)	20(1)	19(1)	-2(1)	6(1)	-1(1)
C(5)	20(1)	22(1)	15(1)	-1(1)	3(1)	0(1)
C(9)	23(1)	23(1)	23(1)	0(1)	2(1)	3(1)
C(8)	21(1)	32(1)	28(1)	-2(1)	0(1)	-1(1)
C(23)	19(1)	18(1)	18(1)	2(1)	4(1)	1(1)
C(24)	22(1)	22(1)	24(1)	3(1)	7(1)	3(1)
C(28)	25(1)	19(1)	24(1)	1(1)	9(1)	1(1)
C(29)	28(1)	18(1)	19(1)	1(1)	4(1)	2(1)

C(17)	21(1)	20(1)	22(1)	1(1)	4(1)	-1(1)
C(22)	24(1)	22(1)	25(1)	4(1)	6(1)	-1(1)
C(18)	21(1)	24(1)	22(1)	2(1)	4(1)	1(1)
C(26)	26(1)	22(1)	29(1)	5(1)	6(1)	-3(1)
C(25)	26(1)	26(1)	28(1)	6(1)	12(1)	2(1)
C(21)	30(1)	24(1)	29(1)	1(1)	13(1)	-1(1)
C(19)	24(1)	24(1)	24(1)	-2(1)	4(1)	3(1)
C(20)	21(1)	27(1)	32(1)	-4(1)	10(1)	0(1)
C(10)	23(1)	21(1)	16(1)	-1(1)	4(1)	0(1)
C(11)	32(1)	19(1)	27(1)	2(1)	9(1)	0(1)
C(12)	48(1)	28(1)	25(1)	4(1)	8(1)	3(1)
C(34)	32(1)	21(1)	19(1)	0(1)	2(1)	3(1)
C(33)	35(1)	32(1)	21(1)	1(1)	7(1)	7(1)
C(32)	42(1)	30(1)	26(1)	-2(1)	7(1)	15(1)
C(31)	35(1)	27(1)	26(1)	2(1)	5(1)	10(1)
C(30)	33(1)	19(1)	22(1)	2(1)	6(1)	1(1)
C(13)	77(1)	23(1)	28(1)	-1(1)	12(1)	6(1)
C(14)	23(1)	20(1)	23(1)	2(1)	4(1)	0(1)
C(15)	36(1)	32(1)	27(1)	8(1)	5(1)	-2(1)
C(16)	32(1)	22(1)	30(1)	-4(1)	8(1)	-6(1)

3. Computational Studies

3.1. General

All computational studies were carried out without symmetry restrictions. If it was not possible to obtain starting coordinates from crystal structures either *GaussView 3.0*[13] or *GaussView 6.0*[14] were used. Calculations were performed either with the *Gaussian09 Revision E.01*[15], the *Gaussian16 Revision B.01*[16] or the *Gaussian16 Revision C.01*[17] program packages using Density-Functional Theory (DFT) [18,19]. Energy optimizations were carried out with the PBE0[20] functional and def2svp basis set [21] together with GRIMMES D3 dispersion correction with Becke-Johnson damping [22–24]. To determine the nature of the structure harmonic vibrational frequency analyses were performed on the same level of theory.[25] No imaginary frequencies were observed for the ground states, for transition states one imaginary frequency corresponding to the translational motion was observed. Single point energies were calculated on PBE0[20]/def2tzvp[21] level of theory. Additionally, single point energies were calculated on the same level of theory with the Polarizable Continuum Model (PCM) using the integral equation formalism variant (IEFPCM)[26] as implemented in Gaussian09 and Gaussian16 with the parameters for tetrahydrofurane. The energies in the manuscript are corrected for the solution change by 7.925867 kJ/mol for each species to convert them to a 1 M standard state. Values given in the following tables are not solvent corrected.

3.2. Energies and coordinates for the AYC with a SCy2 onium moiety

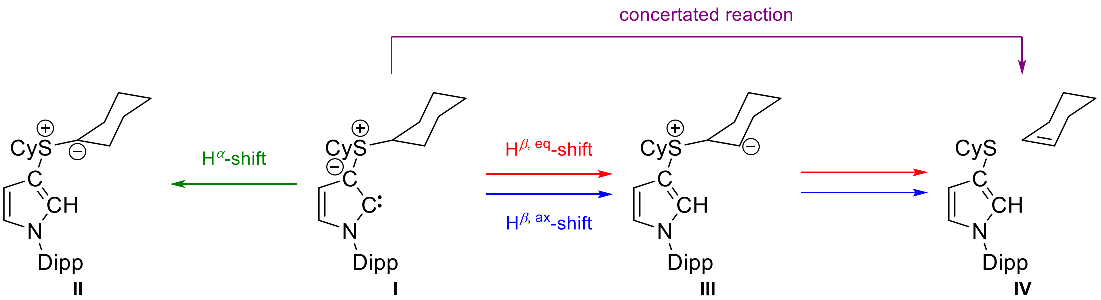

	Corr(H)	Corr(G)	E(SCF)	E(PCM)	ΔG in hartree	ΔG in kJ/mol
**I**	0.667489	0.573813	-1543.50584236	-1543.51981401	0	0
**TS**$_{I\rightarrow II}$	0.662041	0.568255	-1543.49198700	-1543.49744263	0.016813	44.143529
**II**	0.666700	0.570817	-1543.53560927	-1543.54019511	-0.023377	-61.376576
**TS**$_{I\rightarrow III}^{ax}$	n.d.	n.d.	n.d.		-	-
**III**^{ax}	n.d.	n.d.	n.d.		-	-
**TS**$_{III\rightarrow IV}^{ax}$	n.d.	n.d.	n.d.		-	-
**TS**$_{I\rightarrow III}^{eq}$	n.d.	n.d.	n.d.		-	-
**III**^{eq}	n.d.	n.d.	n.d.		-	-
**TS**$_{III\rightarrow IV}^{eq}$	n.d.	n.d.	n.d.		-	-
Cyclohexene	0.153142	0.118108	-234.450673317	-234.451720805	-0.094395	-247.833639
Sulfide	0.511769	0.428793	-1309.12987765	-1309.13557604		
**TS**$_{I\rightarrow IV}$	0.661755	0.569421	-1543.48380362	-1543.48995202	0.025470	66.871459

Coordinates of the optimized structure of **I**

E = -1543.50584236
C -0.871931 0.405351 -0.653254
C 0.299544 0.768716 0.029869
C 0.084920 1.035756 1.424952
C -1.265919 0.864844 1.585352

N -1.798979 0.483918 0.361992
H 0.783447 1.355357 2.196369
H -1.896561 1.003454 2.462939
C -3.179962 0.173236 0.198374
C -3.607878 -1.146249 0.428111
C -4.071838 1.191688 -0.179244
C -4.967116 -1.432942 0.272193
C -5.422357 0.859953 -0.323938
C -5.867073 -0.438735 -0.098278
H -5.328588 -2.449278 0.441860
H -6.138680 1.629841 -0.617773
H -6.927083 -0.679134 -0.213452
C -2.601205 -2.229850 0.760445
H -1.761325 -1.737048 1.276590
C -3.556886 2.583459 -0.484873
H -2.655196 2.732435 0.130649
C 3.044395 1.491486 0.047982
C 3.603137 0.827881 1.299997
C 4.153628 1.933959 -0.904772
H 2.464240 2.382335 0.347672
C 4.561680 1.782751 2.009092
H 4.152217 -0.084625 1.016454
H 2.791634 0.519183 1.972665
C 5.122941 2.871464 -0.189533
H 4.699631 1.048216 -1.271594
H 3.716599 2.425522 -1.787524
C 5.681069 2.243019 1.082307
H 4.975222 1.291781 2.904070
H 3.997129 2.661054 2.369860
H 5.937868 3.155206 -0.873878
H 4.593503 3.806311 0.067182
H 6.341536 2.954656 1.602396
H 6.310050 1.374774 0.814265
C 2.118878 -1.219621 -0.864526
C 3.463910 -1.561007 -1.487936
C 1.860717 -1.938046 0.448054
H 1.317684 -1.515716 -1.566744
C 3.560086 -3.073031 -1.693163
H 4.281346 -1.234307 -0.822791
H 3.593315 -1.030454 -2.445276
C 1.951560 -3.444602 0.214030
H 2.605040 -1.643942 1.203418
H 0.869839 -1.651276 0.832141
C 3.292989 -3.835115 -0.398835
H 4.550965 -3.331002 -2.099594
H 2.821046 -3.376987 -2.455194
H 1.788644 -3.978059 1.163769
H 1.133918 -3.754036 -0.460201
H 3.329812 -4.920312 -0.584515
H 4.100158 -3.617094 0.324305
C -3.119806 2.660724 -1.950294
H -2.353195 1.898077 -2.158226
H -3.978859 2.490280 -2.619903

H -2.699761 3.653833 -2.178789
C -4.545865 3.689797 -0.134720
H -5.431865 3.673829 -0.789580
H -4.894499 3.611412 0.906688
H -4.071986 4.675305 -0.262472
C -2.048409 -2.832241 -0.534008
H -2.852472 -3.318978 -1.109718
H -1.600591 -2.041915 -1.157033
H -1.281454 -3.593319 -0.312983
C -3.146592 -3.306814 1.691591
H -3.580299 -2.873025 2.605658
H -3.924484 -3.917307 1.205810
H -2.340059 -3.994739 1.989829
S 1.749704 0.591081 -0.884446

Coordinates of the optimized structure of the **TS**$_{\mathrm{I \rightarrow II}}$

E = -1543.49198700
C -0.477536 0.232655 -0.500378
C 0.534089 -0.111145 -1.373943
C 0.104844 -1.096557 -2.304479
C -1.200967 -1.363084 -1.948156
N -1.530760 -0.562252 -0.871345
H 0.655389 -1.563054 -3.119904
H -1.927309 -2.051296 -2.379733
C -2.806722 -0.567332 -0.237615
C -3.772148 0.368789 -0.646149
C -3.054457 -1.504960 0.780025
C -5.013812 0.348788 -0.004019
C -4.311343 -1.489836 1.391499
C -5.282487 -0.572544 1.002991
H -5.784011 1.065240 -0.297410
H -4.533274 -2.204332 2.187034
H -6.259979 -0.575030 1.491755
C -3.447314 1.401906 -1.705763
H -2.618362 0.996548 -2.306751
C -1.961371 -2.445848 1.246025
H -1.245441 -2.546461 0.415566
C 1.601021 1.573636 0.387083
C 1.894793 0.805802 1.658928
C 2.001907 3.036127 0.440408
H 0.234074 1.210826 0.212971
C 1.209484 1.508913 2.831940
H 2.979949 0.726177 1.874146
H 1.499719 -0.219017 1.576611
C 1.307508 3.711269 1.621305
H 3.098273 3.164469 0.562089
H 1.730019 3.544059 -0.499373
C 1.602657 2.978114 2.924718
H 1.446485 0.983456 3.771602
H 0.116589 1.433679 2.694441
H 1.620634 4.765921 1.691512
H 0.217954 3.711778 1.442207

H 1.079623 3.461644 3.765863
H 2.683637 3.053602 3.146144
C 3.350182 -0.427304 -1.134025
C 4.661697 0.251333 -0.758969
C 3.083274 -1.677507 -0.311932
H 3.393665 -0.711640 -2.202564
C 5.821991 -0.732347 -0.894515
H 4.595230 0.608961 0.282838
H 4.826795 1.139617 -1.389854
C 4.242837 -2.656087 -0.485644
H 2.994449 -1.409307 0.750748
H 2.125555 -2.129997 -0.610415
C 5.572658 -2.012960 -0.103894
H 6.758124 -0.251396 -0.569205
H 5.956674 -0.983638 -1.961925
H 4.061599 -3.557723 0.120656
H 4.285541 -2.993096 -1.537253
H 6.401848 -2.721911 -0.257563
H 5.561656 -1.776544 0.975421
C -1.207456 -1.819833 2.421616
H -0.808140 -0.833487 2.144817
H -1.874219 -1.687662 3.289096
H -0.365258 -2.458692 2.732789
C -2.470209 -3.842297 1.588070
H -3.123318 -3.838868 2.475175
H -3.037672 -4.281387 0.753506
H -1.624781 -4.510244 1.814296
C -2.943475 2.688013 -1.045789
H -2.061339 2.483887 -0.421383
H -2.661949 3.432272 -1.807707
H -3.724838 3.131543 -0.407346
C -4.611803 1.679476 -2.651300
H -4.997752 0.752086 -3.101162
H -5.449172 2.180924 -2.140419
H -4.288163 2.344744 -3.466488
S 2.013111 0.841127 -1.170227

Coordinates of the optimized structure of **II**

E = -1543.53560927
C 0.567675 0.251027 0.464720
C -0.556440 -0.240441 1.095074
C -0.198388 -1.471665 1.716937
C 1.132968 -1.685968 1.428245
N 1.586760 -0.635724 0.671797
H -0.834434 -2.126562 2.307559
H 1.799540 -2.503150 1.695473
C 2.885916 -0.527514 0.095530
C 3.909355 0.090082 0.834825
C 3.086083 -1.029442 -1.201814
C 5.166551 0.199846 0.234184
C 4.360288 -0.893847 -1.760606
C 5.390119 -0.286313 -1.050279

H 5.983642 0.677609 0.779062
H 4.548068 -1.269346 -2.769311
H 6.379710 -0.189001 -1.503358
C 3.642720 0.669499 2.209388
H 2.709694 0.217062 2.578823
C 1.956722 -1.665710 -1.987477
H 1.108416 -1.797070 -1.298523
C -2.138093 1.646381 -0.199498
C -1.873648 1.209832 -1.613332
C -2.730248 3.018360 -0.017547
H 0.695460 1.161015 -0.115180
C -1.078946 2.280450 -2.366516
H -2.818640 1.036792 -2.174954
H -1.325957 0.253378 -1.638267
C -1.903416 4.059189 -0.774581
H -3.772654 3.073979 -0.404366
H -2.788650 3.277865 1.052462
C -1.724082 3.656445 -2.235122
H -0.977452 2.005112 -3.430290
H -0.055314 2.327833 -1.952022
H -2.379266 5.052870 -0.710628
H -0.911590 4.141328 -0.295308
H -1.128173 4.411419 -2.774532
H -2.715081 3.636605 -2.726425
C -3.256536 -0.833866 1.101617
C -4.662251 -0.267451 1.215905
C -3.102877 -1.711598 -0.127869
H -3.004731 -1.395868 2.019282
C -5.712253 -1.375311 1.192866
H -4.824546 0.421970 0.366858
H -4.754880 0.339727 2.131359
C -4.139516 -2.833063 -0.129055
H -3.248876 -1.084472 -1.023266
H -2.081728 -2.119472 -0.187079
C -5.555456 -2.275404 -0.028204
H -6.722816 -0.936354 1.221767
H -5.613149 -1.985915 2.108558
H -4.030268 -3.446914 -1.038103
H -3.949632 -3.507573 0.725748
H -6.292118 -3.094518 0.002806
H -5.777237 -1.690984 -0.939469
C 1.492149 -0.739781 -3.112522
H 1.178005 0.239059 -2.721918
H 2.296918 -0.567322 -3.845144
H 0.635962 -1.178750 -3.648063
C 2.332826 -3.048333 -2.515832
H 3.149447 -2.997680 -3.253300
H 2.656624 -3.713449 -1.701215
H 1.469517 -3.515214 -3.014525
C 3.410381 2.178835 2.111045
H 2.573865 2.410992 1.435661
H 3.173838 2.601796 3.099813
H 4.306985 2.691235 1.726648

C 4.742668 0.338546 3.214207
H 4.923856 -0.745388 3.270672
H 5.696184 0.826874 2.958270
H 4.458759 0.689542 4.218050
S -2.094390 0.659018 1.124879

Coordinates of the optimized structure of the sulfide

E = -1309.12987765
C -0.107550 -0.879241 0.331627
C 0.874861 -1.495003 -0.424547
C 0.436133 -1.467317 -1.781774
C -0.783827 -0.829120 -1.799889
N -1.107635 -0.481299 -0.510001
H 0.968214 -1.879352 -2.636621
H -1.460218 -0.590748 -2.617901
C -2.249151 0.276585 -0.120747
C -2.134377 1.676973 -0.064693
C -3.439009 -0.396908 0.203211
C -3.255972 2.405413 0.341237
C -4.534073 0.375128 0.602283
C -4.444012 1.761524 0.672043
H -3.198003 3.494908 0.399814
H -5.472506 -0.118211 0.865700
H -5.310603 2.347506 0.987819
C -0.831167 2.375841 -0.398856
H -0.163989 1.627145 -0.852764
C -3.520864 -1.909401 0.169148
H -2.634266 -2.272098 -0.372729
C 3.524390 -0.769909 -0.155521
C 3.165496 0.500661 0.601668
C 4.939305 -1.231013 0.176717
H 3.458257 -0.576099 -1.240793
C 4.175181 1.610988 0.325781
H 3.146688 0.268032 1.681575
H 2.147918 0.822187 0.330040
C 5.953554 -0.122013 -0.091244
H 4.977379 -1.518360 1.243313
H 5.185365 -2.135072 -0.402930
C 5.596032 1.161716 0.650835
H 3.911757 2.512191 0.903524
H 4.118087 1.898670 -0.739901
H 6.963747 -0.461831 0.188976
H 5.985514 0.082784 -1.176644
H 6.318724 1.957991 0.409203
H 5.678466 0.985770 1.738739
H -0.158547 -0.691506 1.401461
C -3.454849 -2.478807 1.587331
H -2.535094 -2.161631 2.100762
H -4.311992 -2.139795 2.191250
H -3.468543 -3.579647 1.566175
C -4.753466 -2.410350 -0.579406
H -5.687750 -2.140682 -0.061977

H -4.800929 -1.995018 -1.597285
H -4.730964 -3.507928 -0.660117
C -0.149275 2.886623 0.871310
H -0.766476 3.646481 1.377158
H 0.027954 2.068681 1.585204
H 0.823809 3.344291 0.633192
C -1.021442 3.494187 -1.420990
H -1.504976 3.121106 -2.336202
H -1.640697 4.313796 -1.023339
H -0.048147 3.925554 -1.701368
S 2.376235 -2.156208 0.200846

Coordinates of the optimized structure of cyclohexene

E = -234.450673317
C 0.692381 1.183518 0.318448
C 1.489993 -0.044822 -0.109326
C -1.489992 -0.044904 0.109303
C -0.692443 1.183495 -0.318425
H 1.880551 0.091545 -1.136077
H 2.385182 -0.162303 0.525139
H 0.582060 1.178539 1.417340
H 1.240795 2.104016 0.063182
H -2.385150 -0.162432 -0.525197
H -1.880600 0.091416 1.136042
H -0.582129 1.178579 -1.417317
H -1.240905 2.103951 -0.063110
C 0.666567 -1.298212 -0.057064
C -0.666497 -1.298245 0.057030
H 1.198800 -2.254117 -0.114244
H -1.198662 -2.254174 0.114447

Coordinates of the optimized structure of $\mathbf{TS}_{I \rightarrow IV}$

E = -1543.48380362
C -0.250415 0.044012 0.458254
C 0.506105 0.184491 1.634053
C -0.291333 -0.008291 2.804058
C -1.548950 -0.278683 2.323602
N -1.506904 -0.232259 0.938735
H 0.016633 0.026268 3.847576
H -2.466355 -0.542586 2.847776
C -2.631943 -0.501608 0.109836
C -2.772918 -1.783540 -0.453999
C -3.565430 0.521048 -0.125987
C -3.884427 -2.023049 -1.265867
C -4.667922 0.232793 -0.937152
C -4.827463 -1.026758 -1.502724
H -4.017701 -3.007717 -1.719373
H -5.409974 1.011119 -1.133288
H -5.693044 -1.235237 -2.136497
C -1.722466 -2.851864 -0.226400
H -1.171988 -2.570149 0.684919
C -3.387801 1.904611 0.466122

H -2.417365 1.912673 0.983847
C 1.774933 2.613702 0.404257
C 3.036404 2.965054 -0.343028
C 0.550842 2.518345 -0.365198
H 1.693458 3.145781 1.358940
C 3.206421 2.313876 -1.725733
H 3.930000 2.816670 0.284556
H 2.964206 4.060068 -0.480640
C 0.700174 2.676783 -1.869402
H 0.119933 1.348387 -0.224817
H -0.274956 3.095742 0.072132
C 1.885691 1.860341 -2.362167
H 3.899232 1.460708 -1.664928
H 3.703889 3.043962 -2.383542
H -0.221562 2.335388 -2.367945
H 0.831051 3.739935 -2.148835
H 1.965526 1.899952 -3.459426
H 1.681345 0.805485 -2.109922
C 2.947093 -0.404095 0.355409
C 4.458034 -0.233452 0.479303
C 2.538691 -1.846136 0.621950
H 2.605409 -0.117129 -0.649421
C 5.180723 -1.167029 -0.488814
H 4.754655 -0.468925 1.516679
H 4.756165 0.810391 0.298104
C 3.254241 -2.779699 -0.349708
H 2.798090 -2.110338 1.662549
H 1.448653 -1.939434 0.523807
C 4.768721 -2.619696 -0.279687
H 6.270254 -1.050840 -0.373663
H 4.944210 -0.864548 -1.525198
H 2.964274 -3.821789 -0.140341
H 2.906573 -2.563977 -1.376018
H 5.261786 -3.267837 -1.021896
H 5.122757 -2.955630 0.711593
C -3.336443 2.976980 -0.620898
H -2.551136 2.755201 -1.358849
H -4.292627 3.055711 -1.162480
H -3.123883 3.964431 -0.181322
C -4.465841 2.207401 1.505993
H -5.471283 2.205583 1.054888
H -4.460117 1.458799 2.312395
H -4.303797 3.197738 1.960190
C -0.717619 -2.854599 -1.380894
H -1.214459 -3.105000 -2.332442
H -0.252387 -1.862189 -1.477927
H 0.076570 -3.598777 -1.206052
C -2.317387 -4.237951 0.003075
H -3.060193 -4.229790 0.815168
H -2.809530 -4.632096 -0.900314
H -1.523853 -4.951483 0.274136
S 2.128224 0.747085 1.534076

3.3 Energies and coordinates for the AYC with a SOCy2 onium moiety

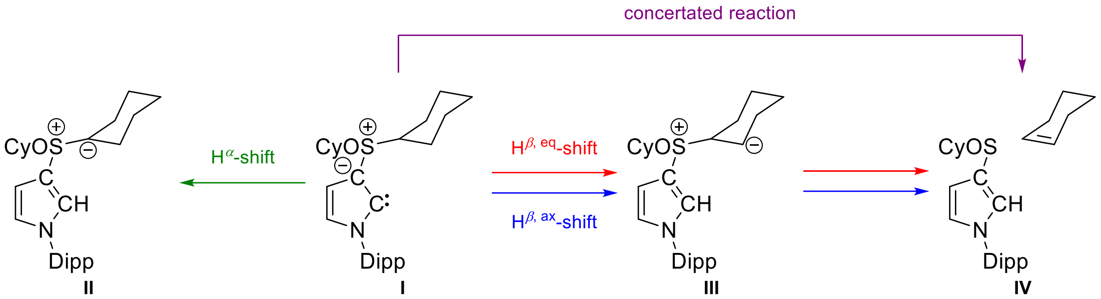

	Corr(H)	Corr(G)	dE(SCF)	E(PCM)	ΔG in hartree	ΔG in kJ/mol
**I**	0.673163	0.577060	-1618.67944897	-1618.69045880	0	0
**TS**I→II	0.667950	0.572916	-1618.65878245	-1618.66703332	0.019281	50.623526
**II**	0.672801	0.575261	-1618.70277293	-1618.71216038	-0.023501	-61.700773
**TS**I→III^{ax}	n.d.	n.d.	n.d.	n.d.	-	-
**III**^{ax}	n.d.	n.d.	n.d.	n.d.	-	-
**TS**III→IV^{ax}	n.d.	n.d.	n.d.	n.d.	-	-
**TS**I→III^{eq}	0.666732	0.571450	-1618.63949720	-1618.64672324	0.038126	100.098658
**III**^{eq}	0.671025	0.576933	-1618.64067585	-1618.64943401	0.040898	107.377148
**TS**III→IV^{eq}	0.669818	0.575899	-1618.63594209	-1618.64392206	0.045376	119.134005
Cyclohexene	0.153142	0.118108	-234.450673317	-234.451720805	-0.080544	-211.469152
Sulfoxide	0.516688	0.431474	-1384.28228984	-1384.29180433		
**TS**I→IV	0.667031	0.570910	-1618.63627910	-1618.64521030	0.039099	102.653112

Coordinates of the optimized structure of **I**

E = -1618.67944897
C 0.709991 0.393210 -0.259761
C -0.291399 0.112313 -1.214331
C 0.166149 -0.757266 -2.264895
C 1.469182 -1.009244 -1.939962
N 1.760598 -0.325760 -0.763442
H -0.367406 -1.121857 -3.141223
H 2.223048 -1.609607 -2.447733
C 3.035709 -0.414583 -0.129433
C 3.235155 -1.400811 0.851680
C 4.052021 0.477301 -0.512679
C 4.493875 -1.480195 1.454682
C 5.295429 0.359654 0.115355
C 5.515636 -0.610390 1.088165
H 4.678625 -2.234327 2.222634
H 6.105078 1.038454 -0.159665
H 6.495026 -0.688472 1.566887
C 2.092274 -2.296103 1.286919
H 1.383155 -2.343698 0.445145
C 3.769686 1.573958 -1.519653
H 2.982484 1.199406 -2.193144
C -2.992830 -0.697813 -1.119747
C -2.584290 -1.922751 -0.315721
C -4.434654 -0.270547 -0.878847

H -2.876026 -0.922452 -2.197187
C -3.531196 -3.074366 -0.649327
H -2.644476 -1.705985 0.760765
H -1.539363 -2.187340 -0.535879
C -5.371173 -1.435630 -1.190824
H -4.558851 0.024906 0.177048
H -4.669229 0.606958 -1.498808
C -4.988584 -2.690298 -0.412410
H -3.260928 -3.957840 -0.050141
H -3.394114 -3.362771 -1.706914
H -6.410352 -1.143787 -0.972003
H -5.329949 -1.648646 -2.273843
H -5.653424 -3.525124 -0.685062
H -5.144302 -2.509823 0.666710
C -1.851002 1.553146 0.564731
C -2.851457 2.699313 0.576158
C -1.967830 0.642988 1.776837
H -0.806265 1.923520 0.540135
C -2.645475 3.520503 1.846839
H -3.882024 2.304896 0.553506
H -2.726591 3.315846 -0.324605
C -1.758048 1.482411 3.035207
H -2.966072 0.174813 1.813380
H -1.209052 -0.149346 1.718573
C -2.730237 2.655104 3.100147
H -3.387848 4.333314 1.884817
H -1.653837 4.004110 1.802946
H -1.860193 0.841733 3.925298
H -0.719427 1.856417 3.036453
H -2.535883 3.263541 3.997683
H -3.760498 2.268143 3.204930
C 3.200490 2.798943 -0.798586
H 2.297306 2.525002 -0.232564
H 3.941209 3.212598 -0.094816
H 2.935462 3.587694 -1.520782
C 4.975332 1.940242 -2.377787
H 5.767775 2.429459 -1.789090
H 5.413407 1.055248 -2.864364
H 4.677300 2.649412 -3.165098
C 1.355560 -1.651281 2.463654
H 2.015699 -1.575473 3.342981
H 1.024861 -0.638377 2.188385
H 0.474013 -2.248482 2.749968
C 2.524994 -3.722728 1.607472
H 3.081960 -4.174568 0.772546
H 3.164163 -3.769191 2.503462
H 1.643802 -4.351327 1.809542
S -1.861003 0.728044 -1.052652
O -2.357031 1.654271 -2.093457

Coordinates of the optimized structure of the **TS**$_{I \rightarrow II}$

E = -1618.65878245

C 0.556930 -0.212583 -0.392223
C -0.485729 0.081705 -1.263019
C -0.058521 0.966720 -2.296206
C 1.265407 1.206530 -2.017724
N 1.614309 0.497634 -0.877399
H -0.624011 1.366829 -3.135392
H 1.999969 1.818003 -2.540803
C 2.917439 0.519627 -0.297041
C 3.833728 -0.483665 -0.654928
C 3.235341 1.541839 0.613465
C 5.102767 -0.443367 -0.069744
C 4.517198 1.542333 1.170601
C 5.442363 0.560102 0.831679
H 5.837777 -1.209646 -0.324360
H 4.795658 2.321238 1.883421
H 6.440274 0.576372 1.276814
C 3.431335 -1.602988 -1.593628
H 2.592300 -1.230594 -2.201969
C 2.192501 2.559614 1.030897
H 1.447137 2.612290 0.221951
C -1.540349 -1.375086 0.693615
C -1.781764 -0.459502 1.877559
C -2.011369 -2.801211 0.899316
H -0.146032 -1.083928 0.438968
C -1.102297 -1.064164 3.107230
H -2.859792 -0.319507 2.096809
H -1.355298 0.538474 1.686225
C -1.322721 -3.375844 2.134677
H -3.109450 -2.855139 1.049656
H -1.787572 -3.407137 0.010234
C -1.554761 -2.497337 3.359008
H -1.300233 -0.433361 3.989233
H -0.010000 -1.050163 2.946558
H -1.683944 -4.400223 2.321863
H -0.238637 -3.449985 1.938484
H -1.033492 -2.912600 4.236821
H -2.632353 -2.500156 3.607535
C -3.284990 0.453899 -1.032644
C -4.599494 -0.208525 -0.640295
C -3.057254 1.783068 -0.330183
H -3.273258 0.607853 -2.128426
C -5.762946 0.740117 -0.915614
H -4.568748 -0.458543 0.434658
H -4.713666 -1.153073 -1.191646
C -4.225283 2.719709 -0.634358
H -2.997960 1.623919 0.756771
H -2.099898 2.224597 -0.645869
C -5.559815 2.093362 -0.241572
H -6.705599 0.278064 -0.582497
H -5.857128 0.885380 -2.006623
H -4.077602 3.676924 -0.109926
H -4.232937 2.954189 -1.714018
H -6.389731 2.773087 -0.492111

H -5.587150 1.960689 0.855129
C 1.466478 2.070486 2.286407
H 1.024936 1.077398 2.119499
H 2.163383 1.994300 3.136557
H 0.658875 2.764889 2.568554
C 2.759418 3.962151 1.224662
H 3.444530 4.015509 2.085479
H 3.309323 4.302824 0.334316
H 1.945386 4.677748 1.417319
C 2.913756 -2.799075 -0.790288
H 2.067130 -2.505640 -0.152618
H 2.573595 -3.601631 -1.463403
H 3.707236 -3.207221 -0.143421
C 4.545815 -2.015148 -2.550274
H 4.944552 -1.151635 -3.104076
H 5.385485 -2.496433 -2.024166
H 4.165434 -2.743598 -3.282444
S -1.942709 -0.772969 -0.894804
O -2.304301 -1.846613 -1.845833

Coordinates of the optimized structure of **II**

E = -1618.70277293
C -0.679137 -0.199119 -0.306250
C 0.494829 -0.824816 0.071062
C 0.167372 -1.808583 1.048981
C -1.192174 -1.724509 1.244860
N -1.691416 -0.746290 0.417186
H 0.842492 -2.494536 1.553626
H -1.855002 -2.277110 1.906808
C -3.057031 -0.336417 0.350862
C -3.908726 -0.972746 -0.567861
C -3.488373 0.696924 1.199602
C -5.235307 -0.536292 -0.626410
C -4.824036 1.097379 1.102543
C -5.688284 0.487841 0.199051
H -5.924532 -1.004633 -1.332447
H -5.192397 1.901606 1.743566
H -6.728760 0.815638 0.136712
C -3.399189 -2.055456 -1.497689
H -2.428434 -2.396393 -1.106517
C -2.537035 1.385408 2.157905
H -1.612121 0.789430 2.190746
C 2.077185 1.114802 -1.014481
C 1.632149 2.141877 -0.011001
C 2.924474 1.611391 -2.154950
H -0.842551 0.591507 -1.033924
C 0.939060 3.301097 -0.730322
H 2.496951 2.561083 0.548151
H 0.963493 1.708245 0.749169
C 2.206119 2.767288 -2.852099
H 3.912572 1.981365 -1.803537
H 3.118296 0.796349 -2.867373

C 1.805132 3.853512 -1.858510
H 0.688561 4.101162 -0.012920
H -0.015384 2.940065 -1.154403
H 2.846156 3.194053 -3.642844
H 1.301522 2.370731 -3.345293
H 1.281157 4.674718 -2.375125
H 2.719527 4.297472 -1.422037
C 3.226624 -1.285852 0.395445
C 4.572913 -1.313559 -0.315412
C 3.296917 -0.516301 1.705035
H 2.866743 -2.318363 0.547636
C 5.661015 -1.871119 0.597089
H 4.834053 -0.281051 -0.612249
H 4.484342 -1.896845 -1.242150
C 4.384229 -1.080404 2.616598
H 3.526779 0.538219 1.471224
H 2.321904 -0.518098 2.217172
C 5.737818 -1.109646 1.915723
H 6.632711 -1.844650 0.078112
H 5.449063 -2.935842 0.803318
H 4.440234 -0.486869 3.543396
H 4.108339 -2.106419 2.920877
H 6.501544 -1.554479 2.573921
H 6.063212 -0.072463 1.716862
C -2.166240 2.776176 1.640731
H -1.721834 2.725582 0.636189
H -3.052464 3.428298 1.583871
H -1.434011 3.256931 2.307958
C -3.090648 1.443702 3.579771
H -3.992411 2.072785 3.643601
H -3.353436 0.441191 3.949516
H -2.342975 1.874378 4.263286
C -3.152146 -1.484492 -2.895502
H -2.437036 -0.649316 -2.866786
H -2.739458 -2.256501 -3.562924
H -4.088480 -1.112102 -3.341276
C -4.323914 -3.269156 -1.544836
H -4.511548 -3.671743 -0.538049
H -5.298145 -3.027698 -1.998338
H -3.871732 -4.067567 -2.152556
S 2.009959 -0.503551 -0.770023
O 2.177755 -1.317610 -1.996814

Coordinates of the optimized structure of $\mathbf{TS}_{\mathrm{I}\rightarrow\mathrm{III}}{}^{\mathrm{eq}}$

E = -1618.63949720
C -0.486521 0.075934 0.281820
C 0.436141 -0.028665 1.322141
C -0.193159 -0.456818 2.525877
C -1.519345 -0.591341 2.191870
N -1.676952 -0.261902 0.855918
H 0.268498 -0.619207 3.496605
H -2.373135 -0.899406 2.792687

C -2.904551 -0.393231 0.139871
C -3.181784 -1.620064 -0.487937
C -3.780548 0.702425 0.082457
C -4.383992 -1.730858 -1.191794
C -4.971473 0.543268 -0.632374
C -5.270405 -0.660547 -1.261873
H -4.631588 -2.668571 -1.693314
H -5.675037 1.375408 -0.699796
H -6.206472 -0.766069 -1.815759
C -2.178801 -2.756739 -0.446525
H -1.556134 -2.605885 0.449741
C -3.403514 2.027655 0.712514
H -2.684695 1.810245 1.518267
C 2.722418 -0.958256 -0.015668
C 4.184646 -0.716448 -0.365792
C 2.509783 -2.282637 0.711618
H 2.075650 -0.889246 -0.906046
C 4.745013 -1.891624 -1.164401
H 4.752680 -0.584067 0.570668
H 4.287387 0.211076 -0.946047
C 3.074722 -3.438433 -0.107343
H 3.018231 -2.230515 1.689755
H 1.437579 -2.432294 0.909049
C 4.544143 -3.218932 -0.444879
H 5.812876 -1.715846 -1.368409
H 4.242883 -1.929576 -2.147467
H 2.938013 -4.379874 0.447604
H 2.492702 -3.539237 -1.041045
H 4.926049 -4.048869 -1.060277
H 5.135725 -3.222202 0.488031
C 1.992389 1.910813 0.100263
C 3.293912 2.702446 0.063106
C 1.289727 1.636923 -1.201831
H 1.298784 2.451397 0.779154
C 2.956702 4.046441 -0.590772
H 4.044270 2.177855 -0.548609
H 3.720141 2.844831 1.070152
C 1.000261 2.995358 -1.826242
H 1.981504 1.081370 -1.869208
H -0.006608 0.704472 -0.698779
C 2.250734 3.880205 -1.936846
H 3.876273 4.643544 -0.702097
H 2.301465 4.615631 0.094008
H 0.544933 2.868811 -2.822898
H 0.245565 3.534228 -1.219163
H 2.002389 4.875391 -2.346242
H 2.951229 3.413773 -2.653619
C -2.684905 2.904702 -0.316076
H -1.799861 2.398382 -0.727073
H -3.355310 3.148156 -1.156017
H -2.354693 3.851133 0.140710
C -4.587888 2.757754 1.337120
H -5.307699 3.101496 0.577512

H -5.128914 2.117682 2.050487
H -4.239772 3.651503 1.876947
C -1.250349 -2.688452 -1.662255
H -1.817682 -2.830358 -2.596154
H -0.743412 -1.713817 -1.715667
H -0.481668 -3.476631 -1.608339
C -2.831344 -4.129988 -0.323033
H -3.523199 -4.174256 0.531599
H -3.395432 -4.402059 -1.229107
H -2.061924 -4.904013 -0.179321
S 2.089016 0.364232 1.057330
O 2.883845 0.429484 2.300085

Coordinates of the optimized structure of **III**^{eq}

E = -1618.64067585
C -0.295112 -0.032770 1.083618
C 0.367825 -0.278149 2.319694
C 1.698754 -0.414162 2.003480
H -0.097226 -0.336483 3.300241
H 2.556534 -0.640685 2.631229
C -2.712118 -1.004026 -0.031941
C -2.375134 -2.330095 0.638804
C -4.215189 -0.781836 -0.149237
H -2.204621 -0.887501 -1.003700
C -3.037943 -3.485387 -0.104788
H -2.733586 -2.297817 1.682343
H -1.284294 -2.469461 0.674520
C -4.868683 -1.951526 -0.881286
H -4.637022 -0.682134 0.865491
H -4.416272 0.157488 -0.683925
C -4.544122 -3.285891 -0.221856
H -2.808855 -4.431445 0.410801
H -2.597865 -3.565431 -1.114934
H -5.957645 -1.792468 -0.923642
H -4.514608 -1.963976 -1.927321
H -4.998433 -4.113950 -0.788894
H -4.993196 -3.312936 0.787072
C -1.633627 1.521018 -1.398642
C -3.281539 2.730989 0.187976
C -1.325890 2.839976 -2.093057
H -2.507799 1.060348 -1.906008
C -2.979916 4.041904 -0.554787
H -4.158388 2.238441 -0.262043
H -3.505498 2.920758 1.252320
C -2.506547 3.825650 -1.996146
H -1.067227 2.684108 -3.154816
H -0.433748 3.315416 -1.635261
H -3.868558 4.693691 -0.532566
H -2.192453 4.580061 0.005041
H -2.258746 4.805191 -2.444974
H -3.345787 3.416249 -2.587878
C 0.638759 -0.033986 0.062177

H 0.455117 0.212795 -0.985618
C -2.034336 1.883442 -0.020358
H -1.197638 2.405831 0.491730
N 1.847782 -0.265240 0.641314
C 3.082072 -0.343340 -0.072936
C 3.912112 0.793748 -0.120089
C 3.416823 -1.549553 -0.721046
C 5.115447 0.684589 -0.826738
C 4.632590 -1.594334 -1.412022
C 5.478438 -0.493829 -1.464836
H 5.773801 1.555903 -0.877581
H 4.913450 -2.521057 -1.919372
H 6.422452 -0.553421 -2.011421
C 3.630997 2.127567 0.560112
H 4.277383 2.839477 0.019902
C 2.208530 2.676910 0.449462
H 1.803344 2.578695 -0.567987
H 1.511504 2.185510 1.142970
H 2.216116 3.747880 0.704489
C 4.113489 2.126801 2.013226
H 3.486462 1.476967 2.640735
H 5.154708 1.779834 2.091707
H 4.058764 3.143317 2.433167
C 2.543984 -2.797039 -0.777708
H 3.238928 -3.592747 -1.094589
C 1.936198 -3.267941 0.542311
H 2.678653 -3.273974 1.353966
H 1.088016 -2.647961 0.863719
H 1.563156 -4.297044 0.422978
C 1.479996 -2.695957 -1.874722
H 0.706815 -1.956697 -1.620556
H 1.923263 -2.403167 -2.837919
H 0.981314 -3.668821 -2.009774
S -1.986666 0.334365 0.959364
O -2.606662 0.330176 2.305667

Coordinates of the optimized structure of $\mathbf{TS}_{III\rightarrow IV}$

E = -1618.63594209
C -0.321460 -0.060201 1.148562
C 0.373782 -0.294720 2.367960
C 1.699448 -0.423131 2.025443
H -0.074203 -0.360683 3.356053
H 2.570634 -0.649033 2.634487
C -2.688119 -1.008356 -0.013664
C -2.342295 -2.371191 0.568454
C -4.191904 -0.802547 -0.146910
H -2.170884 -0.818833 -0.969137
C -2.976538 -3.485554 -0.258078
H -2.715047 -2.408800 1.607011
H -1.250208 -2.496659 0.613410
C -4.816347 -1.927876 -0.967614
H -4.631858 -0.779955 0.865050

H -4.400941 0.170728 -0.615453
C -4.483261 -3.298202 -0.390462
H -2.744735 -4.460256 0.200186
H -2.518220 -3.496732 -1.263479
H -5.906919 -1.782561 -1.020512
H -4.442967 -1.867055 -2.005404
H -4.916142 -4.093675 -1.017907
H -4.949453 -3.396024 0.606247
C -1.659847 1.653978 -1.388592
C -3.294532 2.813768 0.126535
C -1.156474 2.941598 -2.021113
H -2.533586 1.282122 -1.959455
C -2.913727 4.147290 -0.567329
H -4.145037 2.346862 -0.395849
H -3.581545 2.988871 1.178871
C -2.303462 3.979869 -1.967786
H -0.824753 2.807797 -3.065747
H -0.283620 3.337077 -1.466737
H -3.799688 4.801256 -0.616579
H -2.186161 4.667301 0.081813
H -1.975462 4.969519 -2.335542
H -3.095373 3.640997 -2.659761
C 0.593385 -0.053721 0.109343
H 0.422341 0.169545 -0.941966
C -2.041546 2.005303 -0.058360
H -1.215909 2.495668 0.495280
N 1.819437 -0.277306 0.661864
C 3.037060 -0.360414 -0.079184
C 3.874960 0.770744 -0.139598
C 3.352094 -1.566839 -0.737811
C 5.060884 0.657758 -0.874498
C 4.550561 -1.615032 -1.458362
C 5.401108 -0.519185 -1.527540
H 5.723843 1.524938 -0.935446
H 4.815004 -2.541920 -1.974190
H 6.331468 -0.581853 -2.096737
C 3.620117 2.102573 0.554506
H 4.261052 2.812512 0.005193
C 2.200240 2.662349 0.474300
H 1.781249 2.577035 -0.538547
H 1.510174 2.166771 1.171693
H 2.218370 3.730592 0.740270
C 4.132094 2.090672 1.997399
H 3.510871 1.445668 2.635339
H 5.170811 1.731990 2.053069
H 4.097555 3.106226 2.421845
C 2.479046 -2.815395 -0.768977
H 3.166911 -3.610558 -1.102267
C 1.909299 -3.282627 0.569023
H 2.676392 -3.290467 1.357417
H 1.073967 -2.659085 0.915798
H 1.528991 -4.310479 0.462383
C 1.385471 -2.722635 -1.837158

H 0.613175 -1.991006 -1.560106
H 1.800042 -2.425960 -2.811950
H 0.890966 -3.699343 -1.958940
S -2.028845 0.291271 1.082051
O -2.619482 0.114828 2.441685

Coordinates of the optimized structure of the sulfoxide

E = -1384.28228984
C 0.185824 -0.687013 -0.236226
C -0.854773 -1.093154 0.573701
C -0.476202 -0.864856 1.926057
C 0.781548 -0.300722 1.886229
N 1.171679 -0.201053 0.573092
H -1.052787 -1.092440 2.820132
H 1.441459 0.034175 2.683497
C 2.413853 0.332373 0.118644
C 2.496053 1.710345 -0.147219
C 3.500807 -0.539998 -0.058718
C 3.715749 2.208766 -0.612954
C 4.699259 0.006227 -0.527701
C 4.806053 1.365432 -0.802352
H 3.813410 3.273946 -0.833799
H 5.562760 -0.644758 -0.681924
H 5.751151 1.773271 -1.169045
C 1.290461 2.614782 0.014997
H 0.557659 2.075403 0.634728
C 3.370743 -2.026731 0.203517
H 2.437289 -2.180596 0.766093
C -3.372553 -0.355398 -0.194042
C -4.573264 -0.706358 -1.061772
C -3.782171 0.154855 1.177798
H -2.736616 0.382664 -0.714144
C -5.512319 0.488456 -1.189767
H -5.115892 -1.551565 -0.598221
H -4.218426 -1.054308 -2.042838
C -4.734879 1.342469 1.048142
H -4.284451 -0.660932 1.729534
H -2.893765 0.441564 1.761927
C -5.941987 1.007969 0.178292
H -6.391614 0.211316 -1.793025
H -4.998079 1.295405 -1.742129
H -5.057352 1.673668 2.048461
H -4.188479 2.192086 0.600590
H -6.590629 1.892090 0.069264
H -6.550579 0.236373 0.683947
H 0.281327 -0.741402 -1.317557
C 3.242030 -2.792949 -1.114293
H 2.377867 -2.443224 -1.697880
H 4.143287 -2.666435 -1.735540
H 3.107337 -3.869334 -0.926132
C 4.514052 -2.569175 1.057737
H 5.481595 -2.513506 0.534322

H 4.609716 -2.012145 2.001923
H 4.337194 -3.627607 1.302682
C 0.635080 2.877445 -1.342492
H 1.325560 3.406690 -2.018680
H 0.339653 1.937796 -1.831976
H -0.267568 3.497543 -1.225604
C 1.625649 3.918354 0.734778
H 2.109841 3.729224 1.704616
H 2.298874 4.556254 0.140740
H 0.708473 4.498488 0.918920
S -2.307694 -1.860141 -0.099018
O -1.973869 -2.178348 -1.536797

Coordinates of the optimized structure of cyclohexene

E = -234.450673317
C 0.692381 1.183518 0.318448
C 1.489993 -0.044822 -0.109326
C -1.489992 -0.044904 0.109303
C -0.692443 1.183495 -0.318425
H 1.880551 0.091545 -1.136077
H 2.385182 -0.162303 0.525139
H 0.582060 1.178539 1.417340
H 1.240795 2.104016 0.063182
H -2.385150 -0.162432 -0.525197
H -1.880600 0.091416 1.136042
H -0.582129 1.178579 -1.417317
H -1.240905 2.103951 -0.063110
C 0.666567 -1.298212 -0.057064
C -0.666497 -1.298245 0.057030
H 1.198800 -2.254117 -0.114244
H -1.198662 -2.254174 0.114447

Coordinates of the optimized structure of **TS**$_{I \rightarrow IV}$

E = -1618.63627910
C -0.303517 0.047773 -0.408741
C 0.507825 -0.040717 -1.554041
C -0.210856 0.242334 -2.754758
C -1.475466 0.544558 -2.317198
N -1.515747 0.414182 -0.935990
H 0.155845 0.240032 -3.778339
H -2.353426 0.873698 -2.870597
C -2.695387 0.613538 -0.163044
C -2.868916 1.833185 0.515969
C -3.647693 -0.417943 -0.099598
C -4.034640 2.003509 1.267276
C -4.803877 -0.197956 0.655881
C -4.997035 1.000354 1.333603
H -4.194597 2.938814 1.808304
H -5.562674 -0.982002 0.718454
H -5.904974 1.154332 1.921975
C -1.799905 2.906059 0.475551
H -1.165440 2.694095 -0.398928

C -3.441056 -1.732672 -0.825279
H -2.415688 -1.726932 -1.223783
C 3.093023 0.728681 -0.768626
C 2.597490 1.440116 0.477112
C 4.557131 0.322984 -0.691359
H 2.952304 1.386188 -1.646113
C 3.462709 2.672056 0.732046
H 2.659720 0.764363 1.346748
H 1.535533 1.702536 0.367727
C 5.409381 1.563142 -0.428348
H 4.703335 -0.400713 0.127345
H 4.850349 -0.177103 -1.625804
C 4.943240 2.312408 0.816400
H 3.129870 3.171155 1.655910
H 3.307684 3.397816 -0.086350
H 6.467503 1.272182 -0.333272
H 5.349457 2.234347 -1.303623
H 5.550092 3.219722 0.965450
H 5.110968 1.677521 1.705385
C 1.776512 -2.395243 0.057261
C 2.865343 -2.281712 1.106802
C 0.389809 -2.438176 0.470248
H 2.053246 -2.991393 -0.823415
C 2.492224 -3.019134 2.389766
H 3.013085 -1.226031 1.379855
H 3.822277 -2.635592 0.694115
C 0.046575 -2.803452 1.913404
H -0.006640 -1.255710 0.330264
H -0.236924 -2.976218 -0.253767
C 1.160279 -2.493221 2.903517
H 3.291255 -2.880879 3.135559
H 2.419069 -4.104899 2.201460
H -0.880219 -2.285689 2.213595
H -0.176449 -3.884517 1.967279
H 0.917938 -2.927669 3.886330
H 1.237989 -1.401437 3.054273
C -3.558216 -2.928765 0.117610
H -2.867477 -2.834444 0.968270
H -4.577403 -3.033886 0.522225
H -3.318268 -3.862578 -0.414645
C -4.395400 -1.861652 -2.012483
H -5.446393 -1.864334 -1.681120
H -4.269364 -1.026616 -2.717514
H -4.212293 -2.799157 -2.560668
C -0.911307 2.805524 1.717272
H -1.493471 2.998312 2.633115
H -0.472175 1.799752 1.791296
H -0.093151 3.542874 1.673845
C -2.369318 4.311221 0.304092
H -3.025883 4.375686 -0.576742
H -2.951373 4.631902 1.182621
H -1.553474 5.039214 0.174930
S 2.058160 -0.696699 -1.298809

O 2.699525 -1.251205 -2.522597

3.4 Energies and coordinates for the AYC with a PCy_3 onium moiety

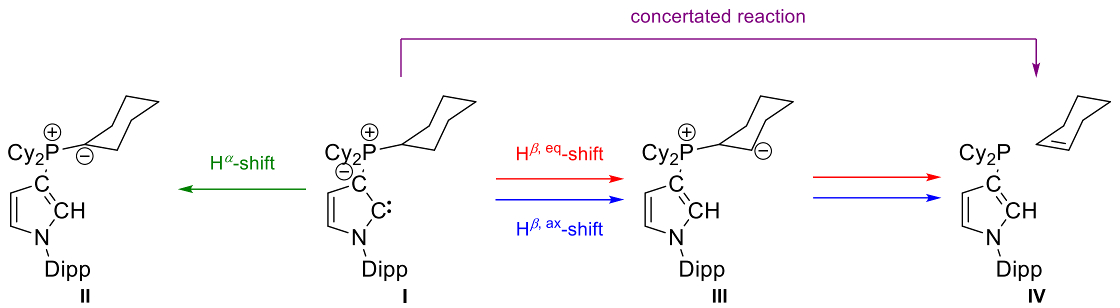

	Corr(H)	Corr(G)	E(SCF)	E(PCM)	ΔG in hartree	ΔG in kJ/mol
**I**	0.835657	0.728915	-1721.81892539	-1721.82900730	0	0
**TS**$_{I\rightarrow II}$	0.830404	0.723482	-1721.79616744	-1721.80168165	0.021893	57.479153
**II**	0.834902	0.726191	-1721.84442875	-1721.84910648	-0.022823	-59.922259
**TS**$_{I\rightarrow II\ I}{}^{ax}$	0.829046	0.721638	-1721.77887401	-1721.79099225	0.030738	80.702750
**III**^{ax}	0.833301	0.724805	-1721.78592159	-1721.79396590	0.030931	81.210391
**TS**$_{III\rightarrow IV}{}^{ax}$	0.832228	0.725315	-1721.78434078	-1721.79099225	0.034415	90.356714
**TS**$_{I\rightarrow III}{}^{eq}$	0.829523	0.723301	-1721.77206284	-1721.77937445	0.044019	115.571491
**III**^{eq}	0.833633	0.725718	-1721.77995851	-1721.78816014	0.037650	98.850495
**TS**$_{III\rightarrow IV}{}^{eq}$	0.832228	0.725315	-1721.78434078	-1721.75911474	0.067127	176.240783
Cyclohexene	0.153142	0.118108	-234.450673317	-234.451720805	-0.043406	-113.963096
Phonphine	0.679533	0.582753	-1487.38698112	-1487.39263874		
TS$_{Conc.}$	0.829148	0.721273	-1721.76364249	-1721.77015748	0.051208	134.446131

Coordinates of the optimized structure of **I**

E = -1721.81892539
C -1.073732 -0.110906 -0.020639
C 0.017230 -0.105214 0.880673
C -0.427333 -0.090379 2.250703
C -1.794768 -0.114160 2.180541
N -2.147059 -0.125932 0.837858
H 0.164132 -0.071415 3.166875
H -2.548989 -0.131488 2.966521
C -3.503718 -0.138689 0.404607
C -4.128528 -1.372799 0.154434
C -4.178144 1.084216 0.243720
C -5.463792 -1.360959 -0.259595
C -5.512826 1.049415 -0.170703
C -6.151599 -0.161826 -0.417001
H -5.974871 -2.304521 -0.462187
H -6.061650 1.983936 -0.305232
H -7.196468 -0.171238 -0.737694
C -3.340949 -2.662765 0.263732
H -2.539122 -2.485087 0.998067
C -3.440717 2.394063 0.437959
H -2.626719 2.200153 1.154513

P 1.633439 0.103377 0.263309
C 2.864147 -0.878831 1.222823
C 2.294698 -2.213428 1.719511
C 4.197916 -1.102242 0.500920
H 3.054599 -0.244342 2.111157
C 3.294932 -2.919237 2.629001
H 2.067191 -2.855203 0.852238
H 1.338006 -2.052914 2.235654
C 5.197740 -1.805606 1.414211
H 4.019726 -1.730951 -0.387618
H 4.619964 -0.156625 0.131657
C 4.641246 -3.122224 1.943317
H 2.880097 -3.885193 2.958227
H 3.438304 -2.316400 3.544284
H 6.142550 -1.974148 0.872883
H 5.440369 -1.141040 2.263342
H 5.359675 -3.592713 2.633566
H 4.515223 -3.824865 1.099794
C 2.070413 1.881671 0.502759
C 3.532483 2.316056 0.394920
C 1.145121 2.761836 -0.343670
H 1.760045 2.009896 1.558905
C 3.677120 3.796287 0.746232
H 3.905358 2.145219 -0.629046
H 4.166831 1.717661 1.066892
C 1.306469 4.231624 0.023537
H 1.387878 2.629979 -1.412401
H 0.102873 2.430308 -0.219156
C 2.759522 4.677564 -0.093698
H 4.728022 4.105664 0.627147
H 3.432033 3.934089 1.814850
H 0.657090 4.848709 -0.617790
H 0.956303 4.385959 1.060010
H 2.867155 5.732553 0.205305
H 3.071706 4.621878 -1.152582
C 1.552517 -0.336084 -1.507540
C 2.661583 0.188806 -2.418361
C 1.309532 -1.835289 -1.712319
H 0.588103 0.155753 -1.752981
C 2.326065 -0.110839 -3.877870
H 3.624822 -0.284085 -2.161947
H 2.799087 1.272193 -2.286998
C 0.975834 -2.115504 -3.172803
H 2.209000 -2.409406 -1.427169
H 0.481397 -2.158309 -1.064880
C 2.062184 -1.594840 -4.106683
H 3.142268 0.242373 -4.528770
H 1.427335 0.465452 -4.160585
H 0.823386 -3.196724 -3.320223
H 0.013745 -1.628952 -3.410605
H 1.786646 -1.774584 -5.158343
H 2.996048 -2.159951 -3.929999
C -2.790097 2.813938 -0.882876

H -2.133103 2.010888 -1.251270
H -3.557849 3.015420 -1.647805
H -2.192233 3.731124 -0.750946
C -4.309954 3.506060 1.014159
H -5.091136 3.828489 0.307228
H -4.806521 3.192296 1.945152
H -3.695029 4.391733 1.237642
C -2.665620 -2.966570 -1.076566
H -3.419636 -3.142542 -1.861300
H -2.034882 -2.115929 -1.378389
H -2.034471 -3.867475 -1.001206
C -4.169619 -3.843965 0.756205
H -4.685175 -3.613236 1.701126
H -4.931997 -4.148950 0.021496
H -3.521423 -4.717650 0.926088

Coordinates of the optimized structure of the **TS**$_{I\rightarrow II}$

E = -1721.79616744
C 0.999863 -0.076277 -0.159652
C -0.139538 -0.115122 -0.965279
C 0.242607 -0.029156 -2.341071
C 1.615381 0.050618 -2.342020
N 2.051280 0.015723 -1.028490
H -0.388805 -0.018689 -3.228509
H 2.322815 0.138350 -3.166406
C 3.413904 0.142889 -0.633972
C 4.244202 -0.991794 -0.658799
C 3.879361 1.404408 -0.224940
C 5.573612 -0.836373 -0.256624
C 5.214591 1.509978 0.176440
C 6.054408 0.401782 0.158942
H 6.244005 -1.697940 -0.263437
H 5.604516 2.476130 0.504203
H 7.096551 0.503208 0.472037
C 3.681047 -2.347609 -1.037433
H 2.862042 -2.169995 -1.752091
C 2.946747 2.598499 -0.177406
H 2.100571 2.371502 -0.844700
P -1.615667 -0.137408 0.025742
C -2.308952 1.568102 0.058575
C -2.231241 2.296036 -1.286368
C -3.700900 1.704211 0.677517
H -1.581002 2.062452 0.730268
C -2.610496 3.764460 -1.119429
H -2.918271 1.821616 -2.008225
H -1.218580 2.203087 -1.705905
C -4.071253 3.174835 0.847126
H -4.439830 1.225527 0.012037
H -3.756353 1.178698 1.642844
C -3.983231 3.927526 -0.475713
H -2.579606 4.273027 -2.096451
H -1.850227 4.259248 -0.488298

H -5.083311 3.260963 1.274435
H -3.384164 3.635963 1.579380
H -4.214304 4.994602 -0.326836
H -4.754453 3.536792 -1.164514
C -0.940130 -0.545665 1.611184
C -1.426227 0.171162 2.857446
C -0.708348 -2.035312 1.832798
H 0.366286 -0.181737 1.093716
C -0.446702 -0.055454 4.008595
H -2.426929 -0.186623 3.184963
H -1.529652 1.254814 2.685374
C 0.264489 -2.244434 2.992340
H -1.646156 -2.585708 2.057677
H -0.286722 -2.494629 0.921554
C -0.214592 -1.541281 4.256164
H -0.818556 0.432847 4.925000
H 0.514647 0.425681 3.756049
H 0.406367 -3.321881 3.179713
H 1.249747 -1.839341 2.703237
H 0.509076 -1.686313 5.075146
H -1.161540 -2.003318 4.593201
C -2.798251 -1.369061 -0.661543
C -3.946418 -1.726219 0.286551
C -3.317065 -1.053507 -2.065466
H -2.141835 -2.256957 -0.740046
C -4.747939 -2.901005 -0.266065
H -4.615869 -0.859220 0.404105
H -3.556365 -1.958107 1.289147
C -4.130486 -2.222325 -2.615082
H -3.957422 -0.155058 -2.024358
H -2.481905 -0.818152 -2.742111
C -5.263894 -2.611891 -1.672002
H -5.585023 -3.137622 0.410278
H -4.103337 -3.798080 -0.289183
H -4.527588 -1.969398 -3.611457
H -3.459561 -3.088257 -2.758195
H -5.809243 -3.483430 -2.068379
H -5.995319 -1.784422 -1.624637
C 2.378412 2.772627 1.233006
H 1.856176 1.859774 1.554654
H 3.182099 2.985707 1.956534
H 1.662937 3.610487 1.263691
C 3.598021 3.883956 -0.679320
H 4.400959 4.230080 -0.009224
H 4.029982 3.752500 -1.682996
H 2.852338 4.692348 -0.732161
C 3.066371 -3.015708 0.195105
H 3.836626 -3.206525 0.959776
H 2.292953 -2.374130 0.641021
H 2.603198 -3.979076 -0.071764
C 4.694773 -3.261623 -1.716423
H 5.171981 -2.771060 -2.578531
H 5.491087 -3.580553 -1.025143

H 4.198268 -4.175741 -2.076276

Coordinates of the optimized structure of the **II**

E = -1721.84442875
P 1.865220 -0.007979 0.057081
N -1.952497 -0.233839 -0.921561
C -0.914594 -0.224191 -0.039762
H -1.071657 -0.256649 1.035725
C 0.269868 -0.165917 -0.754468
C -1.462936 -0.175117 -2.205159
H -2.142706 -0.169988 -3.054350
C -0.087902 -0.132092 -2.138188
H 0.588597 -0.086402 -2.989151
C 2.318769 4.089929 -0.759401
H 3.322162 4.276165 -0.335074
H 2.284583 4.627884 -1.720849
C -5.294444 1.015818 0.046011
H -5.806341 1.963454 0.229775
C -3.948854 1.021634 -0.332943
C -3.329442 -0.219948 -0.554558
C -5.349309 -1.397025 -0.027041
H -5.905280 -2.328351 0.099936
C -5.987664 -0.180677 0.196046
H -7.038991 -0.165980 0.493586
C 5.537417 -0.669260 -1.845861
H 5.439706 -0.100249 -2.788429
H 6.552627 -0.467833 -1.466317
C 2.139550 1.806419 0.313632
H 3.135988 1.893697 0.783705
C 1.093725 2.366107 1.280190
H 0.092540 2.184569 0.852223
H 1.125690 1.811541 2.228575
C 3.070116 -0.471813 -1.299363
H 2.839425 0.109699 -2.211124
C 2.148397 2.592308 -0.999880
H 2.948645 2.232748 -1.664805
H 1.195525 2.412576 -1.529455
C 1.871168 -0.837531 1.525302
C 4.498500 -0.163008 -0.849420
H 4.631933 0.919709 -0.692490
H 4.662571 -0.642023 0.133769
C 2.760874 -0.443458 2.677132
H 3.145276 0.585234 2.573150
H 3.666056 -1.089818 2.742747
C 1.258147 -2.209382 1.661330
H 2.030791 -3.012279 1.640495
H 0.577277 -2.433412 0.823279
C 2.909735 -1.963940 -1.600686
H 3.023989 -2.521598 -0.653968
H 1.889578 -2.174220 -1.959327
C 1.264694 4.633316 0.196953
H 0.267417 4.538793 -0.271359

H 1.424451 5.709110 0.375511
C 1.273911 3.861943 1.510770
H 0.482566 4.232389 2.182904
H 2.232029 4.040628 2.031867
C 5.361823 -2.154419 -2.138648
H 5.568939 -2.731383 -1.219207
H 6.097664 -2.488233 -2.888299
C 3.943558 -2.457773 -2.606939
H 3.816843 -3.539462 -2.777272
H 3.768783 -1.966899 -3.581872
C 2.009627 -0.574056 4.005055
H 2.679585 -0.352842 4.854422
H 1.197887 0.175262 4.028834
C 0.508600 -2.337467 2.989093
H 0.117157 -3.361896 3.118703
H -0.363919 -1.658682 2.974750
C 1.407593 -1.966817 4.164552
H 0.853086 -2.040719 5.115235
H 2.229350 -2.704748 4.228851
C -4.005237 -1.443237 -0.407397
C -3.178537 2.321737 -0.455179
H -2.203753 2.086061 -0.909067
C -2.906102 2.918177 0.926825
H -2.373898 2.203791 1.572269
H -3.844457 3.193763 1.434354
H -2.287104 3.825463 0.843723
C -3.876197 3.324028 -1.371656
H -4.846055 3.648498 -0.962530
H -4.058520 2.894917 -2.368304
H -3.255425 4.224695 -1.496645
C -3.282392 -2.761873 -0.596363
H -2.373269 -2.553486 -1.181560
C -4.106290 -3.782281 -1.376520
H -3.503538 -4.680278 -1.580596
H -4.447277 -3.374064 -2.339882
H -4.995243 -4.110620 -0.815137
C -2.835995 -3.322134 0.756394
H -2.187118 -2.614877 1.292878
H -2.271221 -4.257825 0.622397
H -3.704853 -3.537264 1.398994

Coordinates of the optimized structure of $\mathbf{TS}_{I\rightarrow III}{}^{ax}$

E = -1721.77887401
C 0.955671 0.083477 -0.086979
C -0.217586 0.156313 -0.840156
C 0.122158 0.161444 -2.233343
C 1.495018 0.111915 -2.286152
N 1.975294 0.071181 -0.990023
H -0.544907 0.201419 -3.093404
H 2.174627 0.093470 -3.136420
C 3.347418 -0.088337 -0.636858
C 3.821700 -1.385351 -0.378411

C 4.164260 1.050417 -0.538686
C 5.164710 -1.524740 -0.015796
C 5.501379 0.860785 -0.179168
C 5.996557 -0.414000 0.078981
H 5.566471 -2.518009 0.195058
H 6.165317 1.723026 -0.093309
H 7.044561 -0.542466 0.360844
C 2.888993 -2.579277 -0.439707
H 2.044387 -2.295250 -1.087389
C 3.577116 2.433641 -0.737061
H 2.741162 2.331495 -1.447252
P -1.750278 0.046240 0.028956
C -1.545214 0.702584 1.724615
C -0.404280 -0.083357 2.367176
C -1.240479 2.209346 1.698861
H -2.552238 0.580814 2.203879
C -0.099317 0.450084 3.753254
H 0.743306 0.018020 1.158636
H -0.677468 -1.153260 2.441297
C -0.908988 2.720090 3.097038
H -0.381853 2.393380 1.031516
H -2.095428 2.777393 1.295827
C 0.234455 1.937522 3.727716
H 0.743942 -0.112632 4.189744
H -0.948454 0.319879 4.470356
H -0.676521 3.797216 3.047692
H -1.806446 2.622988 3.736179
H 0.435341 2.318651 4.743545
H 1.153458 2.095020 3.136385
C -2.133062 -1.746744 0.176451
C -3.211730 -2.139278 1.185733
C -2.360802 -2.410673 -1.181515
H -1.164306 -2.108796 0.567264
C -3.259087 -3.658054 1.333393
H -4.198441 -1.781989 0.844747
H -3.012557 -1.667324 2.159659
C -2.418611 -3.928025 -1.028690
H -3.314310 -2.055888 -1.613379
H -1.560571 -2.125108 -1.883168
C -3.471737 -4.348928 -0.009314
H -4.052712 -3.941156 2.043195
H -2.307797 -4.001529 1.777500
H -2.616390 -4.397525 -2.005696
H -1.427025 -4.290445 -0.703449
H -3.465973 -5.443427 0.117460
H -4.473860 -4.087639 -0.396094
C -3.070215 0.871062 -0.951321
C -4.373413 1.041316 -0.162131
C -2.626020 2.204717 -1.566035
H -3.260337 0.167742 -1.784387
C -5.470299 1.639708 -1.037973
H -4.187511 1.704521 0.700227
H -4.708464 0.080651 0.255295

C -3.728936 2.787042 -2.443711
H -2.386473 2.917263 -0.760744
H -1.698959 2.073960 -2.141202
C -5.032422 2.954480 -1.671701
H -6.385318 1.782748 -0.441265
H -5.728319 0.918433 -1.834630
H -3.400058 3.752119 -2.860997
H -3.898310 2.117636 -3.306916
H -5.824456 3.344963 -2.330562
H -4.888141 3.709118 -0.877606
C 2.990257 2.941068 0.583435
H 2.242812 2.239572 0.981176
H 3.781295 3.057984 1.341466
H 2.503139 3.919295 0.444424
C 4.563007 3.435710 -1.326786
H 5.376032 3.676433 -0.623578
H 5.019076 3.059621 -2.255276
H 4.049116 4.381234 -1.557812
C 2.317530 -2.883881 0.948196
H 3.121446 -3.164623 1.647819
H 1.787575 -2.016160 1.368155
H 1.607297 -3.725349 0.895893
C 3.541449 -3.814064 -1.054513
H 3.978949 -3.593142 -2.039968
H 4.339696 -4.221498 -0.414057
H 2.794840 -4.612786 -1.183906

Coordinates of the optimized structure of **III**^{ax}

E = -1721.78592159
C 1.000343 0.093246 0.019583
C -0.211748 0.243639 -0.636730
C 0.076736 0.306634 -2.039675
C 1.442362 0.207337 -2.172469
N 1.988981 0.085337 -0.914813
H -0.629664 0.414526 -2.859975
H 2.077370 0.205220 -3.055665
C 3.362465 -0.170181 -0.628486
C 3.766563 -1.506159 -0.464275
C 4.246866 0.913953 -0.502435
C 5.109054 -1.741537 -0.153763
C 5.580168 0.626710 -0.197523
C 6.005981 -0.686590 -0.022802
H 5.458172 -2.766805 -0.013307
H 6.296466 1.443376 -0.089195
H 7.051958 -0.890003 0.219269
C 2.773618 -2.646923 -0.579834
H 1.890361 -2.258205 -1.110613
C 3.747934 2.339995 -0.624134
H 2.843116 2.315181 -1.251615
P -1.792991 0.184487 0.181795
C -1.801496 1.106449 1.757892
C -0.836180 0.283924 2.574142

C -1.294734 2.542436 1.529548
H -2.878824 1.187284 2.085805
C -0.377214 0.980984 3.837364
H 1.124603 0.005148 1.103056
H -1.279875 -0.688661 2.842903
C -0.897286 3.202205 2.844625
H -0.411207 2.517737 0.868929
H -2.063575 3.151916 1.029328
C 0.155352 2.383894 3.576860
H 0.399264 0.378346 4.341504
H -1.193887 1.097098 4.597586
H -0.543680 4.228132 2.645068
H -1.791308 3.297865 3.488597
H 0.439355 2.884331 4.518574
H 1.065050 2.324003 2.953064
C -2.110778 -1.596416 0.519374
C -3.273557 -1.928758 1.457572
C -2.159358 -2.437651 -0.755993
H -1.177213 -1.838477 1.058121
C -3.240075 -3.414242 1.807558
H -4.238799 -1.697992 0.978545
H -3.211654 -1.317642 2.369844
C -2.143477 -3.923469 -0.406990
H -3.080307 -2.208792 -1.322849
H -1.309069 -2.189490 -1.411692
C -3.267926 -4.286976 0.557027
H -4.085191 -3.663080 2.469275
H -2.321160 -3.624051 2.383424
H -2.212036 -4.527868 -1.325994
H -1.170506 -4.167594 0.056212
H -3.207678 -5.352792 0.830471
H -4.238598 -4.153479 0.044977
C -2.993729 0.736820 -1.118178
C -4.463728 0.563731 -0.726917
C -2.735544 2.175944 -1.581049
H -2.790794 0.062068 -1.971909
C -5.388459 0.958950 -1.876098
H -4.679658 1.184393 0.160502
H -4.669573 -0.475911 -0.437980
C -3.641623 2.542420 -2.751103
H -2.942062 2.861935 -0.744210
H -1.677349 2.321126 -1.843238
C -5.110336 2.368724 -2.382735
H -6.439091 0.862528 -1.558103
H -5.248739 0.241670 -2.705334
H -3.442742 3.578688 -3.068225
H -3.399757 1.899859 -3.617582
H -5.757200 2.600535 -3.244129
H -5.371098 3.096431 -1.593221
C 3.332861 2.870551 0.750501
H 2.560366 2.238024 1.210933
H 4.195760 2.901017 1.434798
H 2.926496 3.890766 0.668194

C 4.749137 3.271712 -1.299250
H 5.643694 3.435412 -0.677886
H 5.080361 2.876303 -2.271495
H 4.292202 4.258323 -1.469587
C 2.309541 -3.105455 0.804936
H 3.154758 -3.496836 1.393598
H 1.853420 -2.282722 1.374673
H 1.560614 -3.908508 0.713837
C 3.316291 -3.815672 -1.398341
H 3.666082 -3.486513 -2.388400
H 4.156401 -4.318068 -0.893475
H 2.530290 -4.571809 -1.547859

Coordinates of the optimized structure of **TS**$_{III \rightarrow IV}$ ^{ax}

E = -1721.78434078
C -0.924055 0.038551 0.056105
C 0.277075 -0.198363 -0.601631
C -0.035779 -0.360117 -1.991049
C -1.399929 -0.238351 -2.115031
N -1.927152 -0.000014 -0.866930
H 0.655527 -0.548572 -2.809044
H -2.045805 -0.288922 -2.988613
C -3.293335 0.297618 -0.591800
C -3.702608 1.642666 -0.629988
C -4.171727 -0.753090 -0.281287
C -5.044123 1.919757 -0.354610
C -5.506085 -0.426072 -0.021222
C -5.938006 0.895357 -0.057510
H -5.395662 2.953668 -0.371529
H -6.217029 -1.219633 0.219252
H -6.984069 1.131189 0.152743
C -2.710664 2.754759 -0.911080
H -1.829322 2.294570 -1.384362
C -3.698521 -2.192170 -0.255441
H -2.598192 -2.171653 -0.223125
P 1.852917 -0.274060 0.220457
C 1.735841 -1.468574 1.690902
C 0.773908 -0.760696 2.558491
C 1.249381 -2.822434 1.143457
H 2.793533 -1.586813 2.035045
C -0.178970 -1.571205 3.406024
H -1.075857 0.191778 1.123239
H 1.251407 0.036783 3.147220
C 0.515021 -3.632730 2.202331
H 0.545297 -2.656468 0.309719
H 2.098027 -3.393604 0.736410
C -0.672113 -2.838552 2.722729
H -1.051534 -0.962509 3.714161
H 0.283462 -1.902387 4.369447
H 0.197314 -4.596374 1.769115
H 1.194578 -3.870912 3.041309
H -1.278370 -3.445772 3.415760

H -1.322453 -2.577380 1.867933
C 2.139122 1.409849 0.902551
C 3.298140 1.559887 1.890486
C 2.157723 2.489379 -0.179172
H 1.205893 1.516382 1.484826
C 3.267602 2.952276 2.516039
H 4.266382 1.414599 1.385324
H 3.228901 0.790098 2.673553
C 2.145289 3.876954 0.455846
H 3.062236 2.387068 -0.805930
H 1.292143 2.364462 -0.849830
C 3.285271 4.047276 1.454430
H 4.117082 3.072581 3.207536
H 2.352239 3.048887 3.126716
H 2.198140 4.651321 -0.326770
H 1.180592 4.021258 0.974603
H 3.234984 5.041346 1.927467
H 4.248416 4.010762 0.912671
C 3.071810 -0.544120 -1.169189
C 4.524043 -0.226737 -0.806602
C 2.967747 -1.976437 -1.707534
H 2.759160 0.157099 -1.966440
C 5.454605 -0.455524 -1.995724
H 4.838993 -0.861789 0.040974
H 4.620302 0.814510 -0.469748
C 3.876245 -2.182840 -2.914055
H 3.270613 -2.674983 -0.909574
H 1.926387 -2.231181 -1.953284
C 5.325351 -1.861109 -2.569181
H 6.496365 -0.253585 -1.697953
H 5.208910 0.280513 -2.782617
H 3.786381 -3.218745 -3.278850
H 3.540491 -1.530766 -3.741078
H 5.970640 -1.974562 -3.455170
H 5.688326 -2.592341 -1.824412
C -4.175929 -2.940580 0.986282
H -3.894347 -2.408880 1.906943
H -5.269073 -3.076361 0.990506
H -3.721056 -3.942023 1.024644
C -4.107990 -2.916275 -1.538930
H -5.204819 -2.955985 -1.637950
H -3.708948 -2.408336 -2.429548
H -3.729662 -3.950464 -1.539808
C -2.242074 3.398575 0.395993
H -3.083590 3.872790 0.926159
H -1.795313 2.654046 1.070762
H -1.485212 4.174008 0.197099
C -3.254880 3.799776 -1.881254
H -3.606947 3.337081 -2.815495
H -4.094001 4.367150 -1.448751
H -2.469441 4.527830 -2.135853

Coordinates of the optimized structure of $\mathbf{TS}_{I\rightarrow III}{}^{eq}$

E = -1721.77206284
C 1.070150 0.067945 -0.092482
C -0.087337 -0.009468 -0.871845
C 0.281268 -0.337357 -2.217758
C 1.652234 -0.431002 -2.223307
N 2.108370 -0.175743 -0.943844
H -0.361423 -0.468862 -3.086298
H 2.346476 -0.661269 -3.029741
C 3.471164 -0.286921 -0.540483
C 3.922093 -1.521429 -0.042162
C 4.308396 0.836007 -0.645915
C 5.258512 -1.612676 0.357153
C 5.637864 0.696114 -0.237187
C 6.108474 -0.515757 0.258387
H 5.640965 -2.556454 0.751555
H 6.315368 1.549638 -0.303752
H 7.151032 -0.605839 0.573456
C 2.969763 -2.691609 0.108138
H 2.136332 -2.521477 -0.591875
C 3.755264 2.166390 -1.114796
H 2.895254 1.946505 -1.767000
P -1.649159 0.319488 -0.110233
C -2.839254 0.427948 -1.518828
C -3.125789 -0.949974 -2.131339
C -4.140796 1.194626 -1.271909
H -2.248567 1.009310 -2.252079
C -3.931735 -0.822429 -3.419223
H -3.704550 -1.549373 -1.408986
H -2.191106 -1.499995 -2.312620
C -4.961790 1.299170 -2.555188
H -4.733784 0.688465 -0.491458
H -3.923744 2.203688 -0.896050
C -5.230969 -0.062812 -3.182484
H -4.136150 -1.823513 -3.831428
H -3.326691 -0.291391 -4.176243
H -5.907778 1.823950 -2.346697
H -4.411986 1.928009 -3.278520
H -5.789278 0.052755 -4.125175
H -5.877698 -0.653726 -2.508663
C -1.410073 1.844358 0.900726
C -2.613399 2.510481 1.579736
C -0.267589 1.540271 1.862985
H -1.028535 2.542016 0.124171
C -2.137148 3.811740 2.230979
H -3.005671 1.836290 2.360204
H -3.447918 2.732077 0.894057
C 0.187888 2.848420 2.487047
H -0.663099 0.893909 2.675205
H 0.857680 0.642885 0.987233
C -0.954883 3.606513 3.174450
H -2.978651 4.290970 2.758814
H -1.840517 4.512074 1.427806
H 1.000916 2.668732 3.212101

H 0.624402 3.505933 1.708337
H -0.620314 4.584399 3.566376
H -1.291698 3.018168 4.047589
C -2.068784 -1.043537 1.052326
C -3.526346 -1.069981 1.519479
C -1.594824 -2.429448 0.608106
H -1.439742 -0.752826 1.913879
C -3.702591 -2.084691 2.646608
H -4.181956 -1.349945 0.676679
H -3.850802 -0.072810 1.853242
C -1.773395 -3.432504 1.742997
H -2.171618 -2.767036 -0.268751
H -0.541353 -2.378055 0.295356
C -3.215536 -3.470633 2.237873
H -4.759536 -2.122006 2.956077
H -3.131516 -1.740762 3.527343
H -1.452470 -4.433494 1.412128
H -1.105502 -3.152043 2.577109
H -3.315819 -4.174001 3.080079
H -3.864372 -3.858586 1.431247
C 3.226545 2.959325 0.083378
H 2.460282 2.394974 0.633522
H 4.043504 3.194967 0.784397
H 2.776803 3.909519 -0.246026
C 4.753164 2.984524 -1.927286
H 5.595954 3.336666 -1.311477
H 5.166769 2.404983 -2.766757
H 4.262185 3.879322 -2.339426
C 2.379930 -2.715360 1.520809
H 3.171167 -2.866928 2.272737
H 1.868590 -1.768228 1.747219
H 1.650995 -3.535548 1.624486
C 3.604431 -4.031191 -0.252601
H 4.054899 -4.008210 -1.256478
H 4.389376 -4.322806 0.463080
H 2.844293 -4.827476 -0.237884

Coordinates of the optimized structure of **III**[eq]

E = -1721.77995851
C 1.086625 0.059638 -0.082483
C -0.078383 -0.028862 -0.830877
C 0.304498 -0.387519 -2.163209
C 1.676248 -0.494775 -2.170087
N 2.136243 -0.219665 -0.902193
H -0.341839 -0.532247 -3.025870
H 2.370848 -0.741490 -2.969991
C 3.496214 -0.293725 -0.480278
C 3.971809 -1.514485 0.027923
C 4.297625 0.856721 -0.569906
C 5.301803 -1.564007 0.454737
C 5.621183 0.756640 -0.131677
C 6.118090 -0.440463 0.374158

H 5.704476 -2.495395 0.858699
H 6.272960 1.630992 -0.183424
H 7.155524 -0.497522 0.712636
C 3.056688 -2.715954 0.161641
H 2.189101 -2.540230 -0.493513
C 3.721803 2.169837 -1.060372
H 2.847518 1.927948 -1.685064
P -1.681493 0.318612 -0.135287
C -2.854038 0.006691 -1.537114
C -2.915876 -1.464662 -1.965564
C -4.274958 0.549442 -1.348516
H -2.386760 0.587691 -2.356007
C -3.712673 -1.624465 -3.256432
H -3.414079 -2.041688 -1.168291
H -1.908733 -1.892623 -2.074864
C -5.086974 0.388649 -2.631338
H -4.773235 0.003727 -0.530504
H -4.256303 1.605142 -1.050287
C -5.119940 -1.057443 -3.111061
H -3.751749 -2.687607 -3.543806
H -3.186365 -1.100383 -4.075009
H -6.110140 0.765709 -2.473106
H -4.641402 1.024046 -3.418089
H -5.668626 -1.134940 -4.063447
H -5.679539 -1.668577 -2.379809
C -1.576318 2.038847 0.504275
C -2.797177 2.951782 0.657915
C -0.717431 1.867464 1.731578
H -0.949942 2.483678 -0.300507
C -2.287373 4.314563 1.141442
H -3.496808 2.524078 1.396887
H -3.357552 3.100472 -0.283520
C -0.224452 3.240299 2.153027
H -1.365002 1.478321 2.546940
H 1.159574 0.380320 0.960120
C -1.380164 4.230218 2.368276
H -3.142550 4.984288 1.333479
H -1.718763 4.776995 0.312420
H 0.382363 3.180440 3.074109
H 0.450361 3.660005 1.378577
H -1.015788 5.243623 2.621772
H -1.976225 3.886930 3.233790
C -1.987660 -0.841725 1.252382
C -3.416859 -0.829811 1.795528
C -1.477656 -2.269808 1.048459
H -1.322022 -0.358230 1.993926
C -3.479634 -1.599812 3.112207
H -4.101482 -1.301885 1.068297
H -3.767733 0.203565 1.943141
C -1.539136 -3.030732 2.369821
H -2.090293 -2.799170 0.300306
H -0.448009 -2.253137 0.661051
C -2.946618 -3.020257 2.957862

H -4.514317 -1.615400 3.491119
H -2.877866 -1.061065 3.865166
H -1.191098 -4.066422 2.223512
H -0.838375 -2.563708 3.084577
H -2.961929 -3.541765 3.928493
H -3.620233 -3.588517 2.290222
C 3.220232 3.001170 0.123543
H 2.451810 2.467384 0.701257
H 4.049398 3.249074 0.805568
H 2.775186 3.945346 -0.227356
C 4.697175 2.963827 -1.923955
H 5.555462 3.334914 -1.341808
H 5.088931 2.358968 -2.755891
H 4.193667 3.844955 -2.349801
C 2.531869 -2.827809 1.595200
H 3.357559 -3.000371 2.304056
H 2.011505 -1.909209 1.903420
H 1.823887 -3.666730 1.687347
C 3.715106 -4.015315 -0.293382
H 4.107133 -3.930580 -1.318074
H 4.549333 -4.307250 0.363799
H 2.984974 -4.838948 -0.274323

Coordinates of the optimized structure of **TS**$_{III\rightarrow IV}$ ^{eq}

E = -1721.75518619
C 0.143467 -0.341876 0.758060
C -0.231511 -0.666022 2.101660
C -1.600603 -0.545952 2.188321
H 0.426651 -0.943488 2.921749
H -2.273238 -0.659263 3.034407
P 1.772657 -0.326387 -0.032295
C 1.941123 1.481982 -0.416543
C 1.906375 2.408104 0.795795
C 3.097414 1.817464 -1.359447
H 1.011191 1.641037 -0.994929
C 1.846557 3.869718 0.360762
H 2.809589 2.251697 1.412749
H 1.043247 2.158910 1.432813
C 3.025942 3.280424 -1.789218
H 4.064905 1.632215 -0.863415
H 3.067343 1.156420 -2.239668
C 2.989872 4.218466 -0.586653
H 1.861224 4.530492 1.243213
H 0.882537 4.050325 -0.148099
H 3.877894 3.526550 -2.443909
H 2.113592 3.429419 -2.394540
H 2.907792 5.265985 -0.919670
H 3.947741 4.141670 -0.039804
C 2.893914 -0.707041 1.416473
C 2.801161 -2.207839 1.717807
C 4.347941 -0.313421 1.155665
H 2.527379 -0.135382 2.289918

C 3.698920 -2.610311 2.882549
H 3.107583 -2.761210 0.811315
H 1.758668 -2.502275 1.913405
C 5.257889 -0.724170 2.310679
H 4.691427 -0.796305 0.222018
H 4.430064 0.771264 0.993579
C 5.146922 -2.210966 2.625336
H 3.622252 -3.695452 3.059677
H 3.341781 -2.118666 3.805871
H 6.301358 -0.455054 2.078692
H 4.980159 -0.142344 3.208253
H 5.780756 -2.469696 3.489037
H 5.534359 -2.792763 1.769324
C 0.347598 -0.184158 -2.879825
C 2.218709 -1.725315 -2.988597
C -0.629231 -0.897078 -3.782331
H 0.954994 0.554239 -3.430291
C 1.416616 -2.482175 -4.097556
H 2.846738 -0.940963 -3.442079
H 2.870914 -2.422584 -2.431277
C 0.297962 -1.664678 -4.774099
H -1.319552 -0.239717 -4.339325
H -1.246531 -1.619374 -3.218084
H 2.112269 -2.856117 -4.867525
H 0.970683 -3.377013 -3.628482
H -0.288058 -2.332875 -5.431470
H 0.762577 -0.913813 -5.437271
C -1.031862 -0.026332 0.091500
H -1.176586 0.237130 -0.955153
C 1.092962 -1.142078 -2.204786
H 0.481269 -1.941865 -1.751164
N -2.076453 -0.155065 0.962445
C -3.440496 0.102742 0.638963
C -4.290851 -0.984232 0.350999
C -3.893571 1.437897 0.600878
C -5.625277 -0.699804 0.039023
C -5.237633 1.657213 0.278314
C -6.100297 0.604393 0.002953
H -6.298873 -1.530505 -0.188247
H -5.607738 2.685541 0.247308
H -7.145830 0.801572 -0.245240
C -3.889577 -2.453682 0.376149
H -4.673325 -2.950998 -0.219961
C -2.559760 -2.813518 -0.285363
H -2.446122 -2.322411 -1.262826
H -1.691619 -2.544924 0.332620
H -2.521767 -3.901632 -0.450358
C -3.986107 -3.043437 1.785665
H -3.193500 -2.648146 2.436776
H -4.958143 -2.818426 2.249723
H -3.869716 -4.138052 1.749244
C -3.034673 2.673923 0.838243
H -3.768835 3.469479 1.050763

C -2.092839 2.632833 2.039713
H -2.603457 2.270286 2.944025
H -1.214795 1.996192 1.865630
H -1.725583 3.650265 2.246580
C -2.295778 3.103944 -0.432755
H -1.493547 2.398203 -0.690718
H -2.979698 3.170014 -1.291866
H -1.834781 4.093576 -0.286032

Coordinates of the optimized structure of the phosphine

E = -1487.38698112
C -0.658249 -0.428077 0.486356
C 0.337100 -0.179148 1.415786
C -0.234210 0.709570 2.380361
C -1.529740 0.973257 1.994067
N -1.783754 0.267743 0.845926
H 0.259398 1.104688 3.266928
H -2.299684 1.601043 2.437374
C -2.960736 0.368400 0.051597
C -4.040443 -0.493157 0.310847
C -2.988204 1.323369 -0.980328
C -5.170811 -0.381374 -0.503868
C -4.139382 1.396036 -1.769831
C -5.220105 0.552549 -1.534196
H -6.026114 -1.038321 -0.331239
H -4.190132 2.125054 -2.582213
H -6.112379 0.623458 -2.161057
C -3.955636 -1.544369 1.398742
H -3.136303 -1.248895 2.071806
C -1.799023 2.223699 -1.251752
H -1.091003 2.093182 -0.419415
P 2.038916 -0.816975 1.490347
C 2.126090 -1.818869 -0.088715
C 2.090801 -1.103242 -1.439403
C 3.300224 -2.803942 -0.041740
H 1.204813 -2.429225 -0.005173
C 2.007879 -2.100814 -2.591889
H 3.013107 -0.508302 -1.554065
H 1.255722 -0.388664 -1.484555
C 3.238292 -3.799077 -1.196872
H 4.251006 -2.243937 -0.092567
H 3.306504 -3.331866 0.925075
C 3.162614 -3.095548 -2.547726
H 1.995519 -1.566799 -3.556455
H 1.049983 -2.649618 -2.528817
H 4.108226 -4.475313 -1.163491
H 2.344599 -4.437239 -1.070640
H 3.069708 -3.832957 -3.361708
H 4.109282 -2.553602 -2.726812
H -0.664150 -1.038802 -0.412873
C 2.988763 0.784002 1.225347
C 4.476162 0.517662 0.985989

C 2.447440 1.818637 0.238649
H 2.907800 1.235682 2.234653
C 5.286610 1.809932 1.041576
H 4.608500 0.054361 -0.008086
H 4.856308 -0.210609 1.721665
C 3.255663 3.111706 0.312408
H 2.499837 1.423476 -0.787609
H 1.383497 2.013538 0.444911
C 4.740752 2.856544 0.075806
H 6.347860 1.602922 0.826347
H 5.250591 2.211367 2.070568
H 2.870170 3.842579 -0.417801
H 3.119255 3.568682 1.309647
H 5.313565 3.794382 0.162380
H 4.883990 2.500490 -0.961025
C -1.078360 1.799929 -2.532159
H -0.768415 0.745422 -2.484086
H -1.727044 1.918728 -3.414994
H -0.175692 2.410850 -2.689791
C -2.190041 3.699227 -1.290043
H -2.865475 3.924069 -2.130707
H -2.696794 4.001713 -0.361433
H -1.295108 4.329148 -1.410326
C -3.583684 -2.900583 0.795642
H -4.357902 -3.244667 0.090927
H -2.630812 -2.844390 0.249036
H -3.477349 -3.662157 1.583899
C -5.229149 -1.636204 2.234417
H -5.508201 -0.656902 2.651497
H -6.083219 -2.008044 1.646488
H -5.084621 -2.334803 3.072640

Coordinates of the optimized structure of cyclohexene

E = -234.450673317
C 0.692381 1.183518 0.318448
C 1.489993 -0.044822 -0.109326
C -1.489992 -0.044904 0.109303
C -0.692443 1.183495 -0.318425
H 1.880551 0.091545 -1.136077
H 2.385182 -0.162303 0.525139
H 0.582060 1.178539 1.417340
H 1.240795 2.104016 0.063182
H -2.385150 -0.162432 -0.525197
H -1.880600 0.091416 1.136042
H -0.582129 1.178579 -1.417317
H -1.240905 2.103951 -0.063110
C 0.666567 -1.298212 -0.057064
C -0.666497 -1.298245 0.057030
H 1.198800 -2.254117 -0.114244
H -1.198662 -2.254174 0.114447

Coordinates of the optimized structure of $\mathbf{TS}_{\mathrm{I}\rightarrow\mathrm{IV}}$

E = -1721.76364249
C -0.944422 -0.278950 -0.008525
C 0.193335 -0.500533 -0.789674
C -0.202484 -0.750472 -2.145309
C -1.575385 -0.702915 -2.146261
N -2.004595 -0.428590 -0.859445
H 0.428449 -0.931748 -3.014242
H -2.290821 -0.830295 -2.956704
C -3.361394 -0.169907 -0.509339
C -3.825985 1.156555 -0.571101
C -4.183399 -1.235797 -0.109473
C -5.153036 1.400315 -0.207750
C -5.504604 -0.943876 0.243723
C -5.984934 0.360319 0.196036
H -5.543184 2.419843 -0.240493
H -6.167133 -1.750524 0.565011
H -7.020189 0.569571 0.476737
C -2.896330 2.286732 -0.968152
H -2.072351 1.838039 -1.545138
C -3.632546 -2.642688 0.000711
H -2.725015 -2.684374 -0.621294
C 1.504541 -0.991271 1.906380
C 2.450287 -0.248415 2.850621
C 0.118656 -1.095064 2.389095
H 1.931540 -1.980751 1.673739
C 1.913944 1.101444 3.354607
H 3.460228 -0.128677 2.421223
H 2.571414 -0.923641 3.713775
C -0.274008 -0.154625 3.507842
H -0.744464 -0.539905 1.215729
H -0.224803 -2.131237 2.498030
C 0.386973 1.201920 3.291914
H 2.366373 1.935422 2.793559
H 2.249019 1.237258 4.395869
H -1.372214 -0.022071 3.532662
H -0.001165 -0.521406 4.523135
H 0.031785 1.948943 4.019766
H 0.075182 1.573168 2.299330
C 2.075463 1.561543 -0.074605
C 3.511793 1.994830 0.225513
C 1.610570 2.155031 -1.408168
H 1.416376 1.969179 0.713450
C 3.626901 3.516899 0.245310
H 4.179464 1.599405 -0.560099
H 3.862811 1.578510 1.181346
C 1.736530 3.675509 -1.403225
H 2.220749 1.735181 -2.229390
H 0.571201 1.858206 -1.607560
C 3.150481 4.131072 -1.065024
H 4.668520 3.809385 0.454522
H 3.019344 3.911173 1.079866
H 1.421865 4.078841 -2.379312
H 1.032092 4.085466 -0.657001

H 3.201701 5.230678 -1.016013
H 3.834635 3.824203 -1.877112
C -3.209074 -2.925608 1.443594
H -2.457414 -2.200051 1.785923
H -4.074754 -2.870260 2.123357
H -2.771714 -3.932970 1.530977
C -4.597781 -3.703828 -0.519059
H -5.498484 -3.786223 0.109815
H -4.923836 -3.485252 -1.547370
H -4.112811 -4.692140 -0.517596
C -2.280680 2.929921 0.276527
H -3.058861 3.387187 0.908743
H -1.747167 2.178412 0.876707
H -1.565232 3.719177 -0.006860
C -3.565686 3.327544 -1.860406
H -4.032183 2.863768 -2.742740
H -4.344896 3.892243 -1.324346
H -2.822604 4.059843 -2.212332
P 1.722855 -0.259128 0.045699
C 3.042247 -1.203570 -0.842158
C 4.355731 -1.311656 -0.056220
C 2.542972 -2.606716 -1.212757
H 3.235089 -0.642953 -1.777173
C 5.411093 -2.077571 -0.848989
H 4.162309 -1.843063 0.891773
H 4.740358 -0.320146 0.216351
C 3.596813 -3.379411 -1.999313
H 2.301831 -3.157108 -0.285575
H 1.604617 -2.548667 -1.780027
C 4.918902 -3.463159 -1.246828
H 6.338613 -2.150444 -0.258658
H 5.665861 -1.503517 -1.758435
H 3.219494 -4.387900 -2.232753
H 3.760199 -2.877382 -2.970279
H 5.677544 -3.982707 -1.853982
H 4.779060 -4.072242 -0.335504

3.6 Energies and coordinates for the AYC with a SMe₂ onium moiety

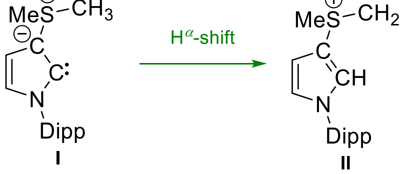

	Corr(H)	Corr(G)	E(SCF)	E(PCM)	ΔG in hartree	ΔG in kJ/mol
**I**	0.411066	0.337071	-1153.08981262	-1153.10096465	0	0
**TS**$_{I\rightarrow II}$	0.405890	0.332847	-1153.06941052	-1153.07586626	0.020874	54.805711
**II**	0.410507	0.334357	-1153.11164091	-1153.11813596	-0.019885	-52.208881

Coordinates of the optimized structure of **I**

E = -1153.08981262
C 1.149095 -0.000215 -0.242527
C 2.206456 -0.000984 0.682012
C 1.768988 -0.001558 2.047670
C 0.403518 -0.001190 1.952276
N 0.068702 -0.000418 0.600390
H 2.362139 -0.002269 2.960855
H -0.363614 -0.001383 2.725374
C -1.287500 0.000214 0.158345
C -1.937097 -1.229391 -0.046090
C -1.935916 1.230411 -0.046149
C -3.269363 -1.204962 -0.469039
C -3.268193 1.207226 -0.469165
C -3.930045 0.001448 -0.677195
H -3.799491 -2.144502 -0.638083
H -3.797403 2.147268 -0.638306
H -4.972634 0.001935 -1.005172
C -1.182090 -2.532559 0.121568
H -0.381090 -2.346628 0.854659
C -1.179798 2.532908 0.121694
H -0.378480 2.345941 0.854169
C -0.506272 2.910027 -1.200394
H 0.150218 2.092869 -1.536374
H -1.261218 3.095935 -1.981818
H 0.097145 3.825322 -1.084397
C -2.039846 3.672168 0.657698
H -2.804167 3.990454 -0.069180
H -2.555988 3.388878 1.587783
H -1.413798 4.552588 0.870091
C -0.508025 -2.909468 -1.200314
H -1.262656 -3.094477 -1.982257
H 0.149212 -2.092612 -1.535571
H 0.094700 -3.825229 -1.084413
C -2.043313 -3.671488 0.656362
H -2.559919 -3.388345 1.586235
H -2.807336 -3.988857 -0.071232
H -1.418026 -4.552453 0.868726
S 3.845201 -0.000883 0.151607
C 3.905369 1.370638 -1.019763
H 4.838745 1.323352 -1.597492
H 3.004585 1.281046 -1.645619
H 3.864009 2.292243 -0.425747
C 3.904923 -1.371019 -1.021347
H 3.004312 -1.280290 -1.647301
H 4.838449 -1.323673 -1.598819
H 3.862839 -2.293288 -0.428398

Coordinates of the optimized structure of the $\mathbf{TS}_{I \rightarrow II}$

E = -1153.06941052
C 1.137784 -0.267704 -0.237440
C 2.215054 -0.415419 0.610408
C 1.820101 -0.361868 1.975247

C 0.459801 -0.145476 1.928388
N 0.070627 -0.093107 0.601879
H 2.425563 -0.454548 2.875507
H -0.268623 -0.033347 2.731180
C -1.277657 0.095969 0.178822
C -2.091967 -1.031969 -0.019884
C -1.745488 1.405640 -0.025724
C -3.408206 -0.820423 -0.441014
C -3.068646 1.568577 -0.445792
C -3.892490 0.466453 -0.651288
H -4.065343 -1.676556 -0.607212
H -3.461606 2.573065 -0.615568
H -4.924838 0.612561 -0.978823
C -1.533562 -2.429507 0.156617
H -0.676098 -2.350186 0.843078
C -0.815919 2.590754 0.143402
H -0.033239 2.286233 0.856085
C -0.123556 2.904556 -1.185442
H 0.423693 2.025802 -1.556963
H -0.861533 3.197805 -1.949632
H 0.591386 3.734687 -1.066379
C -1.506165 3.824387 0.715827
H -2.235170 4.254709 0.010893
H -2.037355 3.593996 1.651825
H -0.764596 4.609558 0.929148
C -0.999026 -2.950587 -1.179702
H -1.811420 -3.031254 -1.920168
H -0.231687 -2.273854 -1.582923
H -0.549007 -3.948619 -1.056633
C -2.531488 -3.402989 0.775714
H -2.944595 -3.014568 1.719057
H -3.375086 -3.616111 0.100033
H -2.040294 -4.364750 0.989131
S 3.761283 -0.707113 -0.208005
C 4.655806 0.826248 0.126905
H 5.625909 0.780015 -0.386138
H 4.051466 1.670201 -0.232215
H 4.803379 0.900806 1.212736
C 3.175460 -0.538622 -1.866133
H 1.818920 -0.393234 -1.461361
H 3.555310 0.394265 -2.303508
H 3.490917 -1.419250 -2.439688

Coordinates of the optimized structure of the **II**

E = -1153.11164091
C 1.099456 -0.248160 -0.360848
C 2.253762 -0.390658 0.379344
C 1.895990 -0.354325 1.757368
C 0.528979 -0.179541 1.801407
N 0.057561 -0.117415 0.514586
H 2.553812 -0.446599 2.618646
H -0.153555 -0.092628 2.644192

C -1.304271 0.079153 0.141636
C -2.125542 -1.046770 -0.039556
C -1.770236 1.392491 -0.042792
C -3.451184 -0.827587 -0.425326
C -3.103068 1.560251 -0.428748
C -3.935380 0.461903 -0.618679
H -4.114632 -1.681624 -0.579549
H -3.495298 2.567672 -0.585312
H -4.974341 0.612932 -0.921658
C -1.585673 -2.452489 0.129562
H -0.611529 -2.371032 0.635204
C -0.850169 2.585216 0.123735
H 0.052899 2.232401 0.644912
C -0.411862 3.116349 -1.242370
H 0.092445 2.335704 -1.830698
H -1.276853 3.472249 -1.824889
H 0.288349 3.958291 -1.126081
C -1.471242 3.686428 0.979561
H -2.347961 4.141846 0.492588
H -1.792905 3.300765 1.958668
H -0.741213 4.491771 1.153476
C -1.339799 -3.097592 -1.235511
H -2.279496 -3.195450 -1.802671
H -0.642356 -2.498864 -1.839684
H -0.907828 -4.103605 -1.118101
C -2.485427 -3.320289 1.006129
H -2.665034 -2.851322 1.985270
H -3.464616 -3.504238 0.536437
H -2.017754 -4.301689 1.178897
S 3.841534 -0.671569 -0.381803
C 4.795111 0.492896 0.687048
H 5.828631 0.451617 0.318682
H 4.384218 1.505546 0.573010
H 4.753857 0.173592 1.738422
C 3.744178 -0.192483 -1.953845
H 0.971620 -0.237395 -1.440382
H 3.490826 0.840292 -2.204252
H 4.299502 -0.821992 -2.647048

3.7 Energies and coordinates for the AYC with a $SOMe_2$ onium moiety

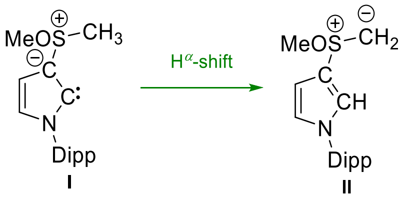

	Corr(H)	Corr(G)	E(SCF)	E(PCM)	ΔG in hartree	ΔG in kJ/mol
**I**	0.415098	0.342229	-1228.23803115	-1228.26512964	0	0
**TS**$_{I\rightarrow II}$	0.411353	0.336934	-1228.23673494	-1228.24591841	0.022958	60.276833
**II**.	0.416453	0.339457	-1228.28527205	-1228.29627208	-0.032366	-84.978088

Coordinates of the optimized structure of the **I**

E = -1228.23803115
C 0.817212 -0.000720 -0.482240
C 1.961326 -0.001014 0.341863
C 1.657318 -0.001327 1.752807
C 0.291405 -0.000676 1.781808
N -0.169727 -0.000385 0.470659
H 2.319398 -0.001730 2.618621
H -0.395010 -0.000529 2.627991
C -1.562963 0.000163 0.157447
C -2.226026 -1.230316 0.012355
C -2.225115 1.231154 0.012520
C -3.591768 -1.205431 -0.285136
C -3.590874 1.207322 -0.284981
C -4.268507 0.001205 -0.430358
H -4.135268 -2.144476 -0.406387
H -4.133679 2.146785 -0.406111
H -5.336806 0.001616 -0.661119
C -1.456973 -2.532943 0.105089
H -0.608583 -2.357472 0.786250
C -1.455101 2.533200 0.105425
H -0.606853 2.357016 0.786578
C -0.869770 2.884879 -1.265227
H -0.229535 2.066851 -1.630453
H -1.675961 3.051611 -1.998075
H -0.267198 3.806051 -1.206290
C -2.273273 3.685510 0.677917
H -3.080036 3.996895 -0.004581
H -2.731233 3.421351 1.643611
H -1.630280 4.565376 0.834351
C -0.871920 -2.884889 -1.265612
H -1.678243 -3.050908 -1.998475
H -0.231051 -2.067313 -1.630735
H -0.270050 -3.806526 -1.206796
C -2.275991 -3.684715 0.677455
H -2.733746 -3.420333 1.643185
H -3.082990 -3.995425 -0.005072
H -1.633645 -4.565073 0.833778
C 4.400655 1.396067 0.315801
H 5.442001 1.336876 -0.027752
H 3.896851 2.274215 -0.109523
H 4.325688 1.404805 1.410908
C 4.402350 -1.394723 0.318504
H 5.443642 -1.334722 -0.025059
H 4.327331 -1.401552 1.413619
H 3.899803 -2.274344 -0.105252
S 3.500609 -0.000531 -0.345616
O 3.601486 -0.001791 -1.801823

Coordinates of the optimized structure of the $\mathbf{TS}_{I \rightarrow II}$

E = -1228.23673494
C 0.931301 -0.053039 -0.264947
C 2.032812 -0.080687 0.587518

C 1.638843 -0.049528 1.955596
C 0.266824 -0.005113 1.911319
N -0.136474 -0.010513 0.583079
H 2.263368 -0.080421 2.845599
H -0.467452 0.022546 2.715681
C -1.503252 0.021483 0.173587
C -2.178903 -1.194934 -0.020696
C -2.124024 1.267921 -0.016802
C -3.515893 -1.138981 -0.425645
C -3.462108 1.273766 -0.420491
C -4.151429 0.082323 -0.623023
H -4.068970 -2.066242 -0.588460
H -3.973935 2.224973 -0.579365
H -5.197661 0.106378 -0.937724
C -1.457693 -2.517565 0.143815
H -0.606320 -2.341143 0.819700
C -1.340751 2.554898 0.149370
H -0.521124 2.345398 0.854867
C -0.702700 2.953108 -1.184086
H -0.061588 2.145185 -1.566651
H -1.477434 3.161839 -1.939463
H -0.088435 3.860472 -1.067111
C -2.168016 3.695876 0.732311
H -2.948590 4.038281 0.034625
H -2.660156 3.401261 1.671555
H -1.523272 4.562896 0.942739
C -0.883957 -2.970610 -1.201044
H -1.691421 -3.149033 -1.929531
H -0.211379 -2.206476 -1.617186
H -0.314585 -3.906543 -1.086132
C -2.326106 -3.602575 0.772635
H -2.769192 -3.266968 1.722519
H -3.147514 -3.912015 0.107100
H -1.722294 -4.499967 0.976576
C 4.208872 1.482449 -0.289395
H 5.174671 1.433017 -0.809790
H 3.492194 2.132299 -0.806585
H 4.350695 1.801007 0.751255
C 3.001433 -0.521394 -1.814811
H 1.668350 -0.228437 -1.485038
H 3.585364 0.037300 -2.556757
H 3.078103 -1.608590 -1.953757
O 4.540527 -1.029409 0.474707
S 3.554886 -0.180158 -0.211821

Coordinates of the optimized structure of the **II**

E = -1228.28527205
C 0.896016 -0.057006 -0.366408
C 2.053812 -0.098475 0.386749
C 1.698814 -0.074869 1.762160
C 0.324681 -0.018396 1.799654
N -0.148292 -0.008498 0.505587

H 2.392919 -0.118814 2.597666
H -0.370101 0.004524 2.636219
C -1.525512 0.019884 0.132269
C -2.198632 -1.200410 -0.048434
C -2.150375 1.265600 -0.047518
C -3.540801 -1.147119 -0.435378
C -3.493659 1.266031 -0.433709
C -4.181534 0.072495 -0.627101
H -4.092990 -2.076757 -0.589958
H -4.009252 2.216612 -0.587396
H -5.231059 0.093229 -0.930398
C -1.488337 -2.527901 0.125273
H -0.540708 -2.328764 0.648690
C -1.386270 2.563265 0.122962
H -0.450565 2.325884 0.652004
C -1.007593 3.142222 -1.241635
H -0.412637 2.426664 -1.828482
H -1.906073 3.391463 -1.828657
H -0.414043 4.062261 -1.123597
C -2.144093 3.581163 0.971541
H -3.065637 3.925597 0.476487
H -2.424165 3.160059 1.948700
H -1.519306 4.469675 1.149783
C -1.140856 -3.131124 -1.237101
H -2.051522 -3.342826 -1.820186
H -0.513270 -2.448886 -1.829351
H -0.589142 -4.075924 -1.113934
C -2.285291 -3.507114 0.983739
H -2.543080 -3.068385 1.959326
H -3.222545 -3.815803 0.494511
H -1.697114 -4.419483 1.165338
C 4.157688 1.513029 -0.573997
H 5.167153 1.468361 -1.005350
H 3.446102 1.964357 -1.273598
H 4.188946 2.038412 0.388174
C 3.580841 -0.907686 -1.688690
H 0.762525 -0.070597 -1.444880
H 4.499037 -0.801031 -2.275766
H 3.164150 -1.917405 -1.615948
O 4.539460 -0.573784 0.942317
S 3.696333 -0.183388 -0.214581

3.8 Energies and coordinates for the AYC with a PMe_2 onium moiety

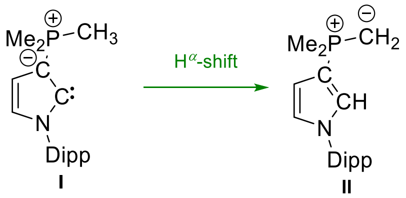

	Corr(H)	Corr(G)	E(SCF)	E(PCM)	ΔG in hartree	ΔG in kJ/mol

**I**	0.449097	0.373027	-1136.19022452	-1136.20506972	0	0
**TS**$_{I \rightarrow II}$	0.444988	0.366319	-1136.17258825	-1136.22135972	-0.021507	-56.466629
**II**	0.449858	0.369924	-1136.21578461	-1136.22399217	-0.020534	-53.913198

Coordinates of the optimized structure of the **I**

E = -1136.19146063
C 0.850412 0.100431 -0.339173
C 1.963769 0.193697 0.531098
C 1.571266 0.187232 1.915906
C 0.205518 0.114824 1.885544
N -0.191166 0.062531 0.553355
H 2.191445 0.246067 2.810254
H -0.524610 0.108408 2.694029
C -1.561571 -0.029485 0.171446
C -2.135356 -1.301638 0.001613
C -2.299280 1.152417 -0.015275
C -3.481812 -1.370421 -0.368162
C -3.642881 1.036369 -0.383749
C -4.229828 -0.212874 -0.557629
H -3.954101 -2.344732 -0.509550
H -4.240512 1.937171 -0.537615
H -5.282245 -0.285168 -0.843736
C -1.288819 -2.549738 0.152472
H -0.483817 -2.304905 0.863814
C -1.624518 2.503318 0.112325
H -0.797002 2.381549 0.829374
P 3.519471 0.084699 -0.206446
C -1.003638 2.896827 -1.230651
H -0.298842 2.119311 -1.563349
H -1.784919 3.015001 -1.999366
H -0.461501 3.852832 -1.144564
C -2.543319 3.596269 0.646987
H -3.339845 3.853580 -0.069491
H -3.023008 3.298860 1.592187
H -1.969163 4.517455 0.831301
C -0.623348 -2.887126 -1.184538
H -1.382934 -3.123006 -1.947878
H -0.027072 -2.030079 -1.534742
H 0.038447 -3.763031 -1.080410
C -2.055994 -3.741989 0.713655
H -2.568212 -3.489917 1.654916
H -2.813830 -4.115534 0.006484
H -1.366376 -4.576676 0.913823
C 4.825019 0.766664 0.847484
H 4.844191 0.216312 1.799355
H 5.808196 0.684137 0.362798
H 4.599800 1.821940 1.055921
C 3.505806 0.962296 -1.780191
H 2.577241 0.640085 -2.277968
H 3.434131 2.041598 -1.588366
H 4.394632 0.733755 -2.384919
C 4.017729 -1.621509 -0.579370

H 4.998870 -1.669780 -1.074961
H 4.040696 -2.196821 0.357204
H 3.241508 -2.050001 -1.230100

Coordinates of the optimized structure of the **TS**$_{I\rightarrow II}$

E = -1136.17258825
C -0.905665 0.000015 -0.231818
C -2.016871 0.000068 0.615092
C -1.593430 0.000106 1.979586
C -0.219901 0.000082 1.935961
N 0.173049 0.000028 0.606854
H -2.199580 0.000149 2.884967
H 0.518880 0.000096 2.737254
C 1.534549 -0.000014 0.184966
C 2.182785 1.230829 -0.015511
C 2.182718 -1.230896 -0.015488
C 3.516767 1.206411 -0.432787
C 3.516702 -1.206558 -0.432763
C 4.177948 -0.000093 -0.638795
H 4.047477 2.146004 -0.599544
H 4.047362 -2.146182 -0.599500
H 5.221472 -0.000125 -0.963557
C 1.429498 2.534744 0.155038
H 0.608349 2.341622 0.863249
C 1.429362 -2.534767 0.155094
H 0.608250 -2.341592 0.863335
C 0.793879 -2.947818 -1.174978
H 0.127930 -2.157133 -1.550458
H 1.569241 -3.135352 -1.935487
H 0.203908 -3.870736 -1.054942
C 2.285151 -3.656026 0.735209
H 3.070771 -3.981050 0.034640
H 2.774361 -3.349627 1.672362
H 1.661334 -4.537608 0.948718
C 0.794084 2.947817 -1.175059
H 1.569481 3.135298 -1.935546
H 0.128103 2.157165 -1.550553
H 0.204160 3.870770 -1.055054
C 2.285332 3.655956 0.735177
H 2.774489 3.349534 1.672349
H 3.070998 3.980927 0.034634
H 1.661560 4.537577 0.948657
C -4.529545 -1.448838 0.129752
H -5.489852 -1.433810 -0.405380
H -3.966050 -2.349750 -0.151415
H -4.707523 -1.470270 1.215222
C -2.942763 0.000198 -1.958631
H -1.588039 0.000021 -1.484740
H -3.217549 -0.908639 -2.510042
H -3.217156 0.909345 -2.509727
P -3.524958 0.000049 -0.303883
C -4.529779 1.448665 0.130127

H -5.490088 1.433608 -0.404998
H -4.707751 1.469799 1.215604
H -3.966442 2.349743 -0.150822

Coordinates of the optimized structure of the **II**

E = -1136.21578461
C 0.871189 0.038377 -0.337903
C 2.040734 0.066754 0.402616
C 1.660082 0.047692 1.779547
C 0.284518 0.010982 1.819045
N -0.182926 0.006800 0.525531
H 2.319540 0.066468 2.644833
H -0.410958 -0.007656 2.655341
C -1.556874 -0.011550 0.144845
C -2.191844 -1.252439 -0.034558
C -2.220250 1.212621 -0.046507
C -3.533005 -1.244003 -0.428239
C -3.560643 1.169082 -0.440738
C -4.210553 -0.046040 -0.630372
H -4.054904 -2.191220 -0.581715
H -4.104061 2.102532 -0.603537
H -5.258497 -0.059658 -0.939672
C -1.437659 -2.554958 0.142587
H -0.505738 -2.323017 0.680510
C -1.497940 2.534250 0.121887
H -0.559808 2.328964 0.659861
P 3.661241 0.100731 -0.366373
C -1.124632 3.116785 -1.242685
H -0.495567 2.420403 -1.816336
H -2.024879 3.330279 -1.841218
H -0.564885 4.057532 -1.123685
C -2.294814 3.532984 0.957474
H -3.221376 3.847231 0.451583
H -2.571863 3.108968 1.934380
H -1.698651 4.440828 1.136502
C -1.048157 -3.134220 -1.218829
H -1.942087 -3.375770 -1.816116
H -0.440295 -2.422236 -1.796319
H -0.462477 -4.058640 -1.095576
C -2.210000 -3.569396 0.982076
H -2.498652 -3.147720 1.956610
H -3.127950 -3.908983 0.476902
H -1.591806 -4.461409 1.166163
C 4.719423 0.809196 0.976900
H 4.671315 0.231427 1.913760
H 5.759843 0.832475 0.619644
H 4.394865 1.842205 1.169337
C 3.557692 0.759583 -1.907713
H 0.742527 0.047847 -1.417925
H 3.077847 1.738547 -1.997680
H 4.392006 0.555888 -2.585287
C 4.296191 -1.605022 -0.464233

H 5.308564 -1.588299 -0.895027
H 4.333779 -2.065795 0.532636
H 3.635295 -2.183822 -1.122293

3.9 Energies and coordinates for the AYC with a StBu2 onium moiety

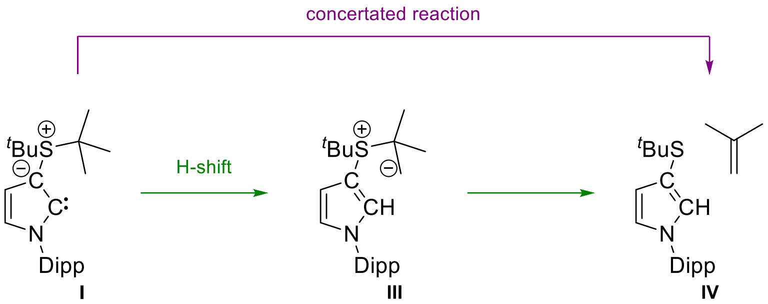

	Corr(H)	Corr(G)	E(SCF)	E(PCM)	ΔG in hartree	ΔG in kJ/mol
**I**	0.588029	0.497060	-1388.78198866	-1388.79044796	0	0
**TS**$_{I\rightarrow III}$	n.d.	n.d.	n.d.	-	-	-
**III**	n.d.	n.d.	n.d.	-	-	-
**TS**$_{III\rightarrow IV}$	n.d.	n.d.	n.d.	-	-	-
Isobutene	0.114127	0.080558	-157.086497623	-157.087805541	-0.093012	-244.202877
Sulfide	0.472246	0.391578	-1231.76514582	-1231.77073037		
TS$_{Conc.}$	0.582459	0.493496	-1388.76052117	-1388.76629945	0.020585	54.044631

Coordinates of the optimized structure of **I**

E = -1388.78198866
C 0.123142 0.011726 -0.049501
C 1.112555 0.023626 0.957117
C 0.555545 0.036360 2.281773
C -0.797532 0.041209 2.081409
N -1.020897 0.025121 0.708392
H 1.078542 0.042884 3.237015
H -1.624597 0.054846 2.789698
C -2.335023 0.011554 0.157259
C -2.961094 -1.224846 -0.078833
C -2.970120 1.234040 -0.120459
C -4.253248 -1.214908 -0.611994
C -4.262197 1.196983 -0.653059
C -4.899264 -0.015638 -0.895537
H -4.763679 -2.160038 -0.808709
H -4.778737 2.131540 -0.882317
H -5.910547 -0.026509 -1.309955
C -2.218345 -2.521066 0.176365
H -1.475737 -2.313508 0.963456
C -2.236266 2.543439 0.086413
H -1.466700 2.360822 0.853042
C -1.509255 2.932520 -1.202791
H -0.846134 2.115642 -1.523620
H -2.230658 3.131950 -2.011994
H -0.903499 3.841431 -1.053721

C -3.132204 3.670201 0.589214
H -3.873710 3.977230 -0.165565
H -3.678555 3.378043 1.499026
H -2.527994 4.559880 0.825458
C -1.445827 -2.928728 -1.080700
H -2.138494 -3.140956 -1.911416
H -0.771151 -2.115928 -1.389395
H -0.847359 -3.836072 -0.896167
C -3.114913 -3.649497 0.673876
H -3.698183 -3.345451 1.556465
H -3.823582 -3.986041 -0.099658
H -2.505950 -4.523503 0.952400
S 2.811708 -0.035366 0.679624
C 3.236318 1.557643 -0.247528
C 3.126179 -1.583108 -0.350328
C 2.398199 -2.668566 0.439529
H 1.311121 -2.518096 0.407069
H 2.638235 -3.640814 -0.018638
H 2.718222 -2.696606 1.492538
C 4.628018 -1.842917 -0.328522
H 4.818039 -2.822797 -0.794167
H 5.195371 -1.095777 -0.897157
H 5.019761 -1.874894 0.699321
C 2.558874 -1.476964 -1.754280
H 2.543789 -2.484078 -2.201590
H 1.527462 -1.085680 -1.715829
H 3.175191 -0.835975 -2.398604
C 4.735387 1.573513 -0.509733
H 5.318201 1.336812 0.393655
H 5.021423 0.880521 -1.312239
H 5.019186 2.585408 -0.839564
C 2.413264 1.748057 -1.509543
H 2.401398 2.820172 -1.762575
H 2.836417 1.206266 -2.364256
H 1.374922 1.403938 -1.349191
C 2.864846 2.612076 0.794489
H 3.131282 3.602556 0.394121
H 1.785363 2.597373 1.001833
H 3.408061 2.465177 1.740694

Coordinates of the optimized structure of the sulfide

E = -1231.76514582
C 0.523738 -0.622400 -0.444312
C 1.599511 -1.106704 0.282518
C 1.184223 -1.167742 1.647212
C -0.112827 -0.709336 1.698301
N -0.504562 -0.382866 0.421681
H 1.784559 -1.518005 2.483966
H -0.800259 -0.582899 2.531838
C -1.762620 0.182045 0.065585
C -2.844130 -0.674372 -0.200491
C -1.872359 1.582061 -0.009181

C -4.066208 -0.089729 -0.546405
C -3.111668 2.118935 -0.368086
C -4.199118 1.292013 -0.631948
H -4.927458 -0.728874 -0.755708
H -3.228086 3.202946 -0.439973
H -5.161402 1.730135 -0.907808
C -2.705169 -2.179959 -0.104410
H -1.641117 -2.400657 0.068476
C -0.683635 2.482575 0.262050
H 0.114022 1.853581 0.686240
C -0.151114 3.083377 -1.039546
H 0.120724 2.297655 -1.759947
H -0.904432 3.730165 -1.517413
H 0.746056 3.692749 -0.848814
C -1.004053 3.565703 1.289731
H -1.764065 4.271657 0.919214
H -1.379733 3.128454 2.226875
H -0.100909 4.149914 1.524271
C -3.110796 -2.871760 -1.404500
H -4.179445 -2.726763 -1.629196
H -2.532712 -2.486983 -2.257907
H -2.932610 -3.955846 -1.334186
C -3.487258 -2.723954 1.091334
H -3.155456 -2.255864 2.030196
H -4.567156 -2.532562 0.984662
H -3.345868 -3.811842 1.185654
S 3.148827 -1.582545 -0.388148
C 4.154128 -0.021021 -0.256977
H 0.419941 -0.438557 -1.510972
C 5.510279 -0.414558 -0.835142
H 5.966115 -1.232913 -0.257039
H 5.416602 -0.742312 -1.881824
H 6.194650 0.448471 -0.806014
C 3.514253 1.086027 -1.082616
H 4.128259 2.001148 -1.031939
H 3.420170 0.788553 -2.137642
H 2.510655 1.331174 -0.703485
C 4.287846 0.398175 1.200511
H 4.921788 1.297644 1.279551
H 3.305419 0.636913 1.634747
H 4.744297 -0.402565 1.801023

Coordinates of the optimized structure of isobutene

E = -157.086497623
C 0.000001 0.120976 -0.000016
C -1.269883 -0.676973 -0.000006
H -2.160832 -0.033790 -0.000473
H -1.319316 -1.336722 -0.883098
H -1.319690 -1.335905 0.883679
C 1.269839 -0.677040 -0.000002
H 1.319559 -1.336244 -0.883489
H 2.160829 -0.033908 0.000219

H 1.319313 -1.336528 0.883289
C 0.000045 1.458922 0.000010
H -0.933374 2.028927 -0.000046
H 0.933502 2.028860 0.000008

Coordinates of the optimized structure of **TS**$_{I\rightarrow IV}$.

E = -1388.76052117
C 0.259020 0.167774 0.073363
C 1.204242 0.159441 1.109875
C 0.576494 0.065170 2.392187
C -0.767841 0.015889 2.119240
N -0.934424 0.068675 0.744162
H 1.046973 0.045801 3.373694
H -1.627972 -0.015900 2.786396
C -2.210953 0.073037 0.113979
C -2.814901 -1.153594 -0.206090
C -2.829919 1.305399 -0.165293
C -4.074755 -1.128386 -0.813157
C -4.085038 1.282990 -0.778579
C -4.704786 0.077679 -1.097249
H -4.568676 -2.069208 -1.069085
H -4.588959 2.223762 -1.010165
H -5.688621 0.080507 -1.573097
C -2.131399 -2.469808 0.104155
H -1.109166 -2.229855 0.432674
C -2.122540 2.609725 0.142837
H -1.400142 2.399630 0.947353
C -1.320407 3.070790 -1.076329
H -0.607260 2.290224 -1.379597
H -1.988317 3.283309 -1.926946
H -0.756128 3.989444 -0.847707
C -3.062579 3.703095 0.640053
H -3.756023 4.041624 -0.146237
H -3.664046 3.361852 1.496337
H -2.484539 4.584033 0.959149
C -2.019656 -3.359220 -1.132211
H -3.007876 -3.687312 -1.492051
H -1.521872 -2.829593 -1.958189
H -1.436907 -4.265920 -0.904475
C -2.827426 -3.192203 1.257336
H -2.848967 -2.565120 2.161100
H -3.868480 -3.445951 0.999716
H -2.303902 -4.129137 1.505753
S 2.887806 0.018721 0.762942
C 3.310731 1.492963 -0.319250
C 2.761994 -1.872484 -0.476510
C 2.571313 -2.778184 0.714401
H 1.631578 -2.544845 1.238899
H 2.504506 -3.819517 0.355241
H 3.404378 -2.714435 1.430019
C 4.139430 -1.921905 -1.080569
H 4.291957 -2.929643 -1.505535

H 4.259381 -1.205435 -1.904194
H 4.930589 -1.752883 -0.333770
C 1.612123 -1.754086 -1.345180
H 0.910923 -2.594661 -1.254752
H 0.870731 -0.813401 -0.926230
H 1.841588 -1.520501 -2.392447
C 4.835939 1.536451 -0.344559
H 5.252776 1.615539 0.670744
H 5.266830 0.648860 -0.828293
H 5.161045 2.419543 -0.917220
C 2.719895 1.381696 -1.715293
H 2.922321 2.318006 -2.260783
H 3.165065 0.558250 -2.289474
H 1.631721 1.229830 -1.656564
C 2.752055 2.716138 0.398416
H 3.060284 3.621158 -0.149986
H 1.654189 2.682033 0.431158
H 3.130876 2.789444 1.428924

3.10 Energies and coordinates for the AYC with a SOtBu2 onium moiety

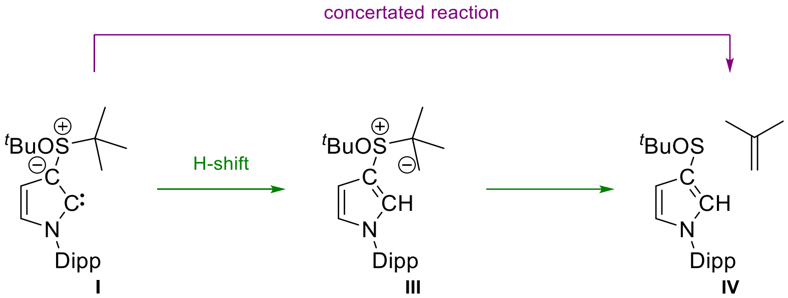

	Corr(H)	Corr(G)	E(SCF)	E(PCM)	ΔG in hartree	ΔG in kJ/mol
**I**	0.593734	0.501517	-1463.93064310	-1463.94630531	0	0
**TS**$_{I\rightarrow III}$	n.d.	n.d.	n.d.	-	-	-
**III**	n.d.	n.d.	n.d.	-	-	-
**TS**$_{III\rightarrow IV}$	n.d.	n.d.	n.d.	-	-	-
Isobutene	0.114127	0.080558	-157.086497623	-157.087805541	-0.097486	-255.950126
Sulfoxide	0.477032	0.393145	-1306.91848182	-1306.92817201		
**TS**$_{I\rightarrow IV}$	0.587307	0.494564	-1463.91754247	-1463.92567426	0.013678	35.911720

Coordinates of the optimized structure of **I**

E = -1463.93064310
C 0.219979 -0.000988 -0.474575
C -0.933243 -0.054584 0.336596
C -0.626531 -0.126598 1.745583
C 0.740495 -0.116346 1.785186
N 1.205501 -0.041052 0.480167
H -1.284331 -0.165807 2.611441
H 1.420949 -0.153884 2.635406
C 2.596543 -0.017715 0.164967
C 3.249058 1.222607 0.064304

C 3.267269 -1.236947 -0.031743
C 4.613507 1.220832 -0.240056
C 4.631487 -1.190704 -0.333395
C 5.298922 0.025906 -0.434505
H 5.149223 2.168201 -0.327215
H 5.181402 -2.120209 -0.493318
H 6.366201 0.042960 -0.669428
C 2.466889 2.512459 0.211950
H 1.624153 2.298806 0.889470
C 2.503986 -2.545792 0.013672
H 1.668169 -2.404286 0.718144
C 1.892068 -2.836351 -1.359867
H 1.247756 -2.001296 -1.677180
H 2.683835 -2.974393 -2.114180
H 1.287143 -3.757539 -1.328428
C 3.336169 -3.720072 0.516466
H 4.131558 -3.995427 -0.194400
H 3.810632 -3.499998 1.485292
H 2.699106 -4.609122 0.642470
C 1.869908 2.912035 -1.140489
H 2.669374 3.128953 -1.867562
H 1.248231 2.094806 -1.539186
H 1.247781 3.816844 -1.039810
C 3.275529 3.649587 0.826324
H 3.738005 3.352630 1.780232
H 4.078057 3.993912 0.154695
H 2.625197 4.517164 1.017754
C -3.248801 1.623398 -0.103927
C -3.450019 -1.512599 0.216402
C -2.444437 -2.660398 0.135408
H -1.644054 -2.557642 0.879793
H -2.985498 -3.602057 0.316615
H -1.981908 -2.708703 -0.861214
C -4.585102 -1.743690 -0.773370
H -5.045526 -2.716226 -0.540968
H -5.368440 -0.978944 -0.706847
H -4.205322 -1.774435 -1.803101
C -3.960113 -1.311251 1.632603
H -4.427874 -2.253031 1.960665
H -3.152268 -1.086278 2.340568
H -4.727061 -0.527175 1.693958
C -4.679102 1.605711 -0.619266
H -4.726521 1.195590 -1.637904
H -5.355603 1.039274 0.036046
H -5.047519 2.642708 -0.655835
C -3.151792 2.007752 1.363204
H -3.541700 3.032218 1.469543
H -3.746788 1.359675 2.019434
H -2.108388 2.006106 1.706901
C -2.377010 2.544538 -0.956721
H -2.722515 3.578223 -0.802136
H -1.318074 2.477269 -0.666487
H -2.455198 2.293614 -2.022177

S -2.453399 -0.046733 -0.430327
O -2.406283 -0.202769 -1.894438

Coordinates of the optimized structure of the sulfoxide

E = -1306.91848182
C 0.390751 -0.494762 -0.435754
C 1.506898 -0.866147 0.291175
C 1.134451 -0.945152 1.659488
C -0.194385 -0.590046 1.723466
N -0.634720 -0.317965 0.450496
H 1.791868 -1.249182 2.470364
H -0.876424 -0.510387 2.567121
C -1.940210 0.139414 0.107167
C -2.943280 -0.805290 -0.166928
C -2.169521 1.525346 0.047690
C -4.208886 -0.324324 -0.515796
C -3.450391 1.957031 -0.307201
C -4.460473 1.041786 -0.586236
H -5.010291 -1.032748 -0.738638
H -3.660574 3.027616 -0.366293
H -5.456007 1.397920 -0.862012
C -2.663132 -2.293616 -0.124121
H -1.658268 -2.427152 0.304000
C -1.062185 2.522434 0.325644
H -0.205624 1.959448 0.727086
C -0.603300 3.194903 -0.968949
H -0.282434 2.450929 -1.713468
H -1.414710 3.788570 -1.419698
H 0.244028 3.871644 -0.777444
C -1.464885 3.550687 1.380353
H -2.293343 4.189322 1.035376
H -1.783209 3.061811 2.313149
H -0.616397 4.211772 1.614736
C -2.640242 -2.877777 -1.537187
H -3.620640 -2.772674 -2.029000
H -1.893729 -2.369551 -2.165747
H -2.388503 -3.949288 -1.510761
C -3.646946 -3.037215 0.776669
H -3.651936 -2.618913 1.794352
H -4.676484 -2.988244 0.388043
H -3.372660 -4.100902 0.847400
C 3.896568 0.418480 -0.446642
C 3.154624 1.215982 -1.502735
H 2.133197 1.464764 -1.179048
H 3.685787 2.162836 -1.691955
H 3.099472 0.669389 -2.458526
C 5.325734 0.100718 -0.873143
H 5.916888 1.028667 -0.923579
H 5.797248 -0.578599 -0.148094
H 5.352675 -0.376620 -1.865578
C 3.863468 1.060147 0.926258
H 4.498034 1.961002 0.938836

H 2.841799 1.351589 1.211697
H 4.247810 0.349983 1.674758
H 0.241505 -0.342631 -1.501943
S 3.118512 -1.294856 -0.316601
O 3.826227 -1.955315 0.842213

Coordinates of the optimized structure of isobutene

E = -157.086497623
C 0.000001 0.120976 -0.000016
C -1.269883 -0.676973 -0.000006
H -2.160832 -0.033790 -0.000473
H -1.319316 -1.336722 -0.883098
H -1.319690 -1.335905 0.883679
C 1.269839 -0.677040 -0.000002
H 1.319559 -1.336244 -0.883489
H 2.160829 -0.033908 0.000219
H 1.319313 -1.336528 0.883289
C 0.000045 1.458922 0.000010
H -0.933374 2.028927 -0.000046
H 0.933502 2.028860 0.000008

Coordinates of the optimized structure of **TS**$_{I\rightarrow IV}$

E = -1463.91754247
C 0.100062 0.156707 -0.049659
C 1.093397 0.180574 0.946963
C 0.538243 0.166615 2.262036
C -0.817841 0.148851 2.054453
N -1.056561 0.133861 0.688525
H 1.068539 0.168300 3.211127
H -1.643531 0.171650 2.763893
C -2.365478 0.092560 0.127647
C -2.966704 -1.156806 -0.095309
C -3.015753 1.301587 -0.178358
C -4.259135 -1.177256 -0.629446
C -4.303601 1.232836 -0.715759
C -4.922276 0.005383 -0.936263
H -4.753118 -2.135455 -0.808735
H -4.833535 2.154307 -0.965756
H -5.931751 -0.028003 -1.353540
C -2.248531 -2.447017 0.245175
H -1.206158 -2.182728 0.478356
C -2.307678 2.627096 0.015731
H -1.539887 2.470116 0.789755
C -1.579851 3.021128 -1.271709
H -0.882716 2.226522 -1.575736
H -2.297592 3.184620 -2.091932
H -1.007529 3.951840 -1.128198
C -3.230516 3.740648 0.499788
H -3.969870 4.026731 -0.265110
H -3.779921 3.446033 1.406803
H -2.645459 4.643260 0.733995
C -2.222713 -3.416577 -0.934384

H -3.230686 -3.781279 -1.188382
H -1.804372 -2.938908 -1.832998
H -1.607393 -4.298252 -0.694975
C -2.848507 -3.095188 1.492823
H -2.806678 -2.411180 2.353391
H -3.903797 -3.368641 1.331401
H -2.299319 -4.011778 1.760516
C 3.275212 1.559802 -0.306683
C 2.540860 -1.905638 -0.860842
C 2.408849 -2.837179 0.312540
H 1.430122 -2.720517 0.801734
H 2.482605 -3.873749 -0.058842
H 3.200744 -2.677057 1.059076
C 3.902853 -1.876273 -1.493924
H 4.088175 -2.860822 -1.958396
H 3.971581 -1.123245 -2.291080
H 4.697240 -1.705188 -0.751077
C 1.358256 -1.713899 -1.665055
H 0.652686 -2.553696 -1.603510
H 0.647272 -0.803680 -1.137294
H 1.545196 -1.401897 -2.700841
C 4.785368 1.446245 -0.456671
H 5.255004 1.201366 0.506274
H 5.071415 0.682158 -1.192224
H 5.179832 2.413407 -0.805282
C 2.548985 1.720618 -1.628999
H 2.763834 2.721770 -2.034950
H 2.878025 0.977140 -2.369702
H 1.459914 1.621642 -1.493405
C 2.904863 2.645840 0.693174
H 3.322299 3.605629 0.349010
H 1.814522 2.748186 0.784608
H 3.324488 2.420839 1.685032
O 3.615601 -0.313848 1.652886
S 2.721922 -0.056851 0.480038

3.11 Energies and coordinates for the AYC with a PtBu3 onium moiety

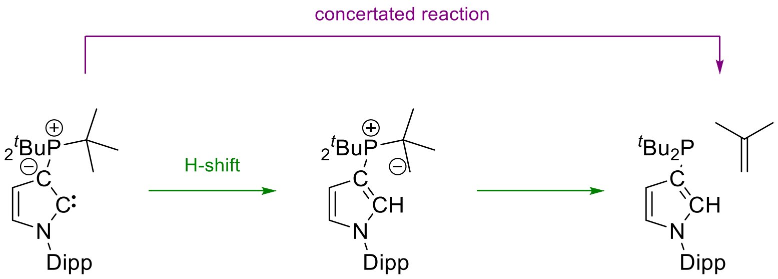

I III IV

	Corr(H)	Corr(G)	E(SCF)	E(PCM)	ΔG in hartree	ΔG in kJ/mol
**I**	0.718515	0.620124	-1489.69360978	-1489.70256786	0	0
**TS**$_{II\rightarrow III}$	0.711756	0.612105	-1489.66083671	-1489.66746849	0.027080	71.099511
III	0.716943	0.615613	-1489.67873242	-1489.68246530	0.015592	40.935641
**TS**$_{III\rightarrow IV}$	0.715518	0.616090	-1489.67071744	-1489.67627127	0.022263	58.450430

Isobutene	0.114127	0.080558	-157.086497623	-157.087805541	-0.073673	-193.427808
Phosphine	0.600704	0.508253	-1332.65188701	-1332.65712207		
**TS**$_{I \rightarrow IV}$	n.d.	n.d.	n.d.	-	-	-

Coordinates of the optimized structure of **I**

E = -1489.69360978
C 0.469862 -0.184649 0.243385
C -0.650181 -0.265777 -0.625937
C -0.227096 -0.176625 -2.007952
C 1.136483 -0.083535 -1.967957
N 1.518925 -0.092425 -0.635953
H -0.818001 -0.191831 -2.920768
H 1.868809 -0.028722 -2.772551
C 2.884227 -0.010406 -0.236670
C 3.618005 -1.197509 -0.068839
C 3.462270 1.255884 -0.038564
C 4.958129 -1.093605 0.315682
C 4.805376 1.313063 0.344742
C 5.547833 0.149590 0.519863
H 5.550841 -1.999895 0.457233
H 5.279316 2.283059 0.509166
H 6.597518 0.212703 0.817832
C 2.945151 -2.544827 -0.235209
H 2.090369 -2.394547 -0.913303
C 2.623270 2.510966 -0.170306
H 1.808228 2.277022 -0.873530
P -2.276008 -0.069096 0.007376
C -2.813535 1.716273 -0.418308
C 1.978239 2.841451 1.177344
H 1.388060 1.982264 1.529955
H 2.748411 3.068995 1.932163
H 1.314619 3.717424 1.091149
C 3.390919 3.702352 -0.732361
H 4.162757 4.063390 -0.034023
H 3.886442 3.454012 -1.683405
H 2.704977 4.544207 -0.914582
C 2.378837 -3.006009 1.109486
H 3.188705 -3.163015 1.840588
H 1.691755 -2.245480 1.508785
H 1.828535 -3.954561 0.999045
C 3.850008 -3.603268 -0.856786
H 4.285446 -3.257714 -1.806938
H 4.679377 -3.883259 -0.187728
H 3.277047 -4.521651 -1.058340
C -2.268857 -0.326037 1.894906
C -1.437728 -1.565468 2.259904
H -0.410071 -1.430224 1.888680
H -1.420594 -1.634577 3.359956
H -1.850453 -2.503776 1.876962
C -1.577918 0.860604 2.580886

H -1.511040 0.626141 3.655434
H -0.557676 0.973156 2.185773
H -2.133725 1.802287 2.489367
C -3.684732 -0.477961 2.461501
H -4.176202 -1.404192 2.136381
H -3.597407 -0.532053 3.558304
H -4.344738 0.367735 2.230040
C -3.462517 -1.315742 -0.812371
C -4.940175 -0.947876 -0.649647
H -5.207824 -0.034816 -1.197292
H -5.548969 -1.765045 -1.069817
H -5.241364 -0.821441 0.397196
C -3.142385 -1.450123 -2.307395
H -3.234578 -0.514030 -2.868134
H -2.133803 -1.853681 -2.460698
H -3.860608 -2.165616 -2.739664
C -1.599346 2.629083 -0.192320
H -0.760508 2.335875 -0.838016
H -1.896018 3.659230 -0.449226
H -1.236493 2.628930 0.839908
C -4.006311 2.221752 0.396384
H -4.266504 3.232241 0.041038
H -4.900532 1.595418 0.279347
H -3.780320 2.306732 1.466842
C -3.155004 1.822548 -1.908898
H -4.075893 1.293096 -2.184615
H -3.309990 2.887918 -2.144171
H -2.329296 1.468766 -2.541088
C -3.211110 -2.704851 -0.214461
H -2.143756 -2.967336 -0.247602
H -3.569189 -2.802426 0.817337
H -3.758303 -3.443095 -0.822564

Coordinates of the optimized structure of **TS**$_{\mathrm{I \rightarrow III}}$

E = -1489.66083671
C 0.399153 -0.001049 -0.106753
C -0.716488 -0.118843 0.733604
C -0.259428 -0.199966 2.094180
C 1.111660 -0.179726 2.038336
N 1.488739 -0.076438 0.713765
H -0.841221 -0.266021 3.010696
H 1.855290 -0.220348 2.832402
C 2.841379 0.006120 0.274458
C 3.404873 1.271941 0.045017
C 3.564631 -1.184770 0.082917
C 4.732635 1.327046 -0.390274
C 4.889887 -1.080242 -0.347531
C 5.469244 0.163516 -0.581750
H 5.196824 2.297238 -0.582348
H 5.478378 -1.985829 -0.507371
H 6.506693 0.225064 -0.919713
C 2.584122 2.534590 0.208368

H 1.681464 2.262709 0.776691
C 2.893208 -2.531329 0.268332
H 2.084448 -2.391587 1.002713
P -2.320044 -0.056134 -0.021502
C -3.576874 -0.705655 1.255284
C -2.177216 -1.098891 -1.739437
C 2.241461 -2.975138 -1.044099
H 1.520121 -2.228831 -1.406140
H 3.003316 -3.119705 -1.827143
H 1.706931 -3.929193 -0.909283
C 3.828837 -3.604736 0.813935
H 4.607576 -3.882667 0.085974
H 4.330794 -3.275770 1.736647
H 3.262221 -4.520536 1.041818
C 2.125856 3.041438 -1.160032
H 2.987151 3.347130 -1.775997
H 1.582631 2.255220 -1.704060
H 1.458755 3.911687 -1.052687
C 3.315397 3.618617 0.995239
H 3.647700 3.247491 1.976537
H 4.202391 3.990801 0.458424
H 2.652568 4.481648 1.163627
C -3.793373 0.298047 2.393271
H -4.428598 -0.177451 3.159083
H -2.854495 0.595617 2.878305
H -4.312728 1.205988 2.060640
C -3.046253 -2.008418 1.870712
H -2.931830 -2.807775 1.131004
H -2.079127 -1.876973 2.369273
H -3.775689 -2.352218 2.622373
C -4.928472 -1.002522 0.597724
H -5.634526 -1.327083 1.379559
H -5.365684 -0.126786 0.100972
H -4.859740 -1.815684 -0.137309
C -3.424940 -0.802843 -2.569499
H -3.416435 -1.487999 -3.432598
H -4.372136 -0.952129 -2.026902
H -3.414915 0.216297 -2.977790
C -0.891309 -0.763066 -2.406835
H -0.373895 -1.667897 -2.757398
H -0.991411 -0.035144 -3.223702
H 0.093644 -0.241478 -1.339831
C -2.176958 -2.564563 -1.307802
H -3.121743 -2.887093 -0.846960
H -2.025887 -3.172067 -2.214124
H -1.344550 -2.781582 -0.621691
C -2.627412 1.785250 -0.394202
C -4.095232 2.101735 -0.686566
H -4.739126 1.972575 0.194214
H -4.172881 3.158449 -0.990413
H -4.502267 1.493949 -1.505704
C -1.771048 2.163204 -1.606841
H -2.123536 1.707187 -2.538811

H -1.815148 3.258023 -1.727183
H -0.720244 1.876711 -1.460442
C -2.128713 2.639272 0.777642
H -2.225651 3.700538 0.495476
H -2.698568 2.490844 1.701698
H -1.069140 2.437653 0.989254

Coordinates of the optimized structure of **III**

E = -1489.67873242
C 1.136090 0.056638 1.924106
C -0.238551 0.083347 1.909063
C -0.663288 0.032479 0.539332
C 0.502862 -0.025246 -0.211968
N 1.575758 -0.014526 0.624579
H 1.845114 0.090350 2.748210
H -0.860883 0.143711 2.794823
P -2.312277 0.067452 -0.225059
C -2.721614 -1.637259 -1.083550
C -3.455709 -0.055326 1.441456
C -2.668937 1.762811 -0.892808
C -1.793151 0.955244 -1.873643
H -2.305640 0.710430 -2.814260
H -0.810778 1.401465 -2.083615
H 0.639046 -0.060851 -1.287807
C -4.037144 2.150882 -1.412379
H -4.674386 2.584412 -0.624380
H -3.916822 2.914345 -2.203194
H -4.579232 1.304678 -1.858193
C -1.935018 2.951495 -0.300697
H -0.977611 2.660971 0.156002
H -1.708323 3.656596 -1.123001
H -2.527353 3.489563 0.454115
C 2.936759 0.000614 0.203612
C 3.607126 -1.221360 0.029870
C 3.549594 1.243008 -0.037060
C 4.934858 -1.176490 -0.405622
C 4.878035 1.236167 -0.471024
C 5.563786 0.039137 -0.653519
H 5.484271 -2.108778 -0.555348
H 5.383893 2.183214 -0.671526
H 6.601932 0.054383 -0.994208
C -3.936793 -1.477206 -2.001442
H -4.143649 -2.442841 -2.493901
H -4.845030 -1.184485 -1.458051
H -3.757403 -0.740448 -2.795997
C -1.513489 -2.045941 -1.925850
H -1.230177 -1.269159 -2.646708
H -0.641760 -2.269553 -1.293603
H -1.763517 -2.963135 -2.484996
C -2.974407 -2.779315 -0.102364
H -3.883375 -2.650723 0.493054
H -3.096414 -3.711542 -0.679622

H -2.126767 -2.925500 0.581202
C -3.095456 -1.148500 2.469123
H -3.137577 -0.744113 3.495471
H -3.805320 -1.986801 2.438341
H -2.094142 -1.577482 2.337305
C -4.930804 -0.199449 1.054049
H -5.567580 -0.036359 1.942566
H -5.236757 0.542777 0.302756
H -5.180709 -1.196204 0.665392
C -3.330412 1.324101 2.097914
H -3.782123 2.106716 1.474613
H -3.873684 1.326022 3.058950
H -2.293634 1.626434 2.300857
C 2.902026 -2.543122 0.257496
H 1.963965 -2.324957 0.790632
C 2.780317 2.539979 0.119665
H 1.882218 2.317995 0.716584
C 2.306737 3.050613 -1.242736
H 3.162128 3.286714 -1.895991
H 1.685728 2.302670 -1.757105
H 1.702939 3.964102 -1.126718
C 3.573491 3.606801 0.869595
H 3.924668 3.235926 1.844243
H 4.453828 3.943436 0.299592
H 2.944706 4.492377 1.048165
C 3.714431 -3.495662 1.130980
H 4.647755 -3.810849 0.638247
H 3.981763 -3.029500 2.091150
H 3.134732 -4.406846 1.344212
C 2.530448 -3.186332 -1.079852
H 1.908856 -2.512733 -1.688133
H 3.431071 -3.431205 -1.665603
H 1.965173 -4.117968 -0.921164

Coordinates of the optimized structure of $\mathbf{TS}_{III\rightarrow IV}$

E = -1489.67071744
C 1.118889 -0.009885 1.969442
C -0.255530 -0.018738 1.972112
C -0.691996 -0.051754 0.603960
C 0.464873 -0.068158 -0.165613
N 1.543791 -0.039752 0.662748
H 1.836047 0.021284 2.786499
H -0.866265 0.015220 2.867955
P -2.341109 0.045681 -0.109226
C -2.694577 -1.525810 -1.147257
C -3.524709 -0.057953 1.457532
C -2.441364 1.676514 -1.032527
C -1.556432 1.125438 -2.110063
H -2.093773 0.881498 -3.037979
H -0.687524 1.764784 -2.325571
H 0.555132 -0.056648 -1.246741
C -3.835481 2.140369 -1.440065

H -4.442741 2.491890 -0.588590
H -3.704758 2.984333 -2.137845
H -4.400295 1.365661 -1.974256
C -1.735375 2.795785 -0.271950
H -0.749493 2.479154 0.095555
H -1.568463 3.605007 -1.002828
H -2.314787 3.202492 0.567918
C 2.902125 -0.014480 0.232390
C 3.571210 -1.233040 0.031539
C 3.513794 1.232015 0.012209
C 4.897419 -1.180312 -0.407427
C 4.840880 1.232904 -0.426066
C 5.526030 0.039672 -0.633291
H 5.445523 -2.109759 -0.578072
H 5.345768 2.183420 -0.611386
H 6.563004 0.061393 -0.977204
C -3.844332 -1.278565 -2.126470
H -4.063586 -2.221028 -2.654718
H -4.769182 -0.958165 -1.627372
H -3.571259 -0.530511 -2.880716
C -1.453559 -1.968337 -1.927896
H -1.085668 -1.146432 -2.553826
H -0.651079 -2.288371 -1.247884
H -1.729379 -2.836232 -2.550450
C -3.049571 -2.697093 -0.226940
H -4.020743 -2.589004 0.267392
H -3.101124 -3.606818 -0.846286
H -2.277615 -2.866234 0.537054
C -3.263130 -1.193799 2.462570
H -3.591788 -0.876128 3.467149
H -3.831871 -2.098451 2.217690
H -2.209014 -1.486767 2.540273
C -4.986404 -0.130969 1.002151
H -5.644095 0.004573 1.877971
H -5.238893 0.659584 0.281851
H -5.248291 -1.097590 0.551437
C -3.359248 1.292489 2.169824
H -3.731600 2.119832 1.551743
H -3.956437 1.285154 3.096904
H -2.320224 1.523008 2.440732
C 2.866131 -2.559201 0.232030
H 1.930250 -2.353372 0.773815
C 2.743126 2.525274 0.188485
H 1.859853 2.297848 0.805444
C 2.236625 3.031564 -1.163809
H 3.077403 3.262574 -1.837480
H 1.599117 2.285078 -1.659237
H 1.639162 3.947641 -1.036329
C 3.546613 3.596432 0.920618
H 3.927096 3.226980 1.884886
H 4.408130 3.943002 0.328259
H 2.914892 4.476051 1.117291
C 3.681073 -3.532484 1.079896

H 4.612343 -3.836569 0.576454
H 3.952439 -3.089297 2.049825
H 3.101682 -4.448279 1.273590
C 2.488818 -3.169832 -1.118980
H 1.867160 -2.480327 -1.708881
H 3.387355 -3.402678 -1.712768
H 1.922308 -4.104028 -0.980536

Coordinates of the optimized structure of the phosphine

E = -1332.65188701
C -0.104936 0.010821 0.062856
C -1.151707 0.021373 0.975250
C -0.543576 0.063174 2.268607
C 0.822746 0.077318 2.096465
N 1.076706 0.041331 0.747297
H -1.076540 0.075077 3.217700
H 1.642965 0.106719 2.810238
C 2.366152 0.015264 0.143892
C 2.980290 1.232357 -0.195906
C 2.967576 -1.230861 -0.106159
C 4.235826 1.177937 -0.808461
C 4.222719 -1.234159 -0.720773
C 4.851073 -0.042164 -1.068332
H 4.738604 2.106605 -1.088816
H 4.716098 -2.185435 -0.932704
H 5.832344 -0.065013 -1.548606
C 2.289777 2.558353 0.051299
H 1.412233 2.357098 0.684476
C 2.256925 -2.525733 0.234186
H 1.430596 -2.274664 0.916911
P -2.956718 -0.065819 0.751828
C 1.640454 -3.144438 -1.022017
H 0.947797 -2.445032 -1.513082
H 2.418913 -3.415887 -1.753180
H 1.076608 -4.056266 -0.770502
C 3.162061 -3.518786 0.958371
H 3.981668 -3.875438 0.314665
H 3.610625 -3.070490 1.857527
H 2.585130 -4.402850 1.270196
C 1.780413 3.151101 -1.263397
H 2.614074 3.381093 -1.946325
H 1.107148 2.451947 -1.781418
H 1.224695 4.084092 -1.080958
C 3.180967 3.543747 0.803622
H 3.538365 3.115545 1.752073
H 4.063513 3.834067 0.211812
H 2.623913 4.464823 1.034284
C -3.188056 -1.575759 -0.387212
C -2.736651 -1.443284 -1.838219
H -2.875124 -2.406824 -2.359644
H -3.320568 -0.690734 -2.387317
H -1.672072 -1.181728 -1.918958

C -2.406836 -2.709595 0.286130
H -2.652514 -3.668110 -0.202012
H -1.320591 -2.554977 0.215991
H -2.664939 -2.797460 1.353773
C -4.677935 -1.935987 -0.345397
H -5.307218 -1.173603 -0.825204
H -4.847420 -2.887966 -0.877152
H -5.027384 -2.056772 0.691245
C -3.326362 1.550313 -0.185989
C -3.220088 2.620066 0.908366
H -2.206591 2.651008 1.339054
H -3.438224 3.615190 0.483857
H -3.930681 2.431086 1.727316
C -2.380041 1.930768 -1.321824
H -1.350842 2.056295 -0.957333
H -2.371540 1.193222 -2.135239
H -2.695760 2.895888 -1.755513
H -0.097205 -0.016544 -1.023303
C -4.766551 1.505342 -0.697530
H -4.877538 0.831914 -1.560499
H -5.466691 1.177344 0.087101
H -5.077458 2.511727 -1.025916

Coordinates of the optimized structure of isobutene

E = -157.086497623
C 0.000001 0.120976 -0.000016
C -1.269883 -0.676973 -0.000006
H -2.160832 -0.033790 -0.000473
H -1.319316 -1.336722 -0.883098
H -1.319690 -1.335905 0.883679
C 1.269839 -0.677040 -0.000002
H 1.319559 -1.336244 -0.883489
H 2.160829 -0.033908 0.000219
H 1.319313 -1.336528 0.883289
C 0.000045 1.458922 0.000010
H -0.933374 2.028927 -0.000046
H 0.933502 2.028860 0.000008

3.12 Energies and coordinates for the AYC with a SAd_2 onium moiety

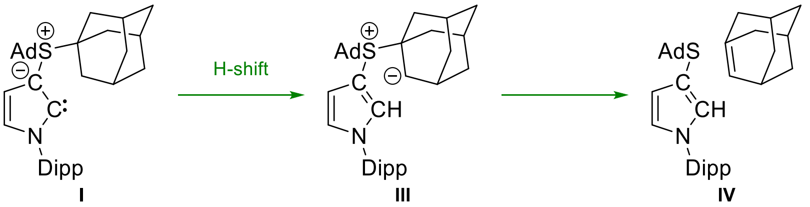

	Corr(H)	Corr(G)	SCF	E(PCM)	ΔG in hartree	ΔG in kJ/mol
**I**	0.817034	0.718733	-1852.99438204	-1853.00280207	0	0
**TS**$_{I\rightarrow IV}$	0.811168	0.712757	-1852.94395874	-1852.94972971	0.047096	123.651493
Adamantene	0.586543	0.501268	-1463.87070410	-389.103682182	-0.006197	-16.268916

Sulfide	0.226862	0.188585	-389.101497486	-1463.87643639		

Coordinates of the optimized structure of **I**

E = -1852.99438204
C 0.990972 -0.032723 0.210695
C 0.118526 -0.035319 1.320904
C 0.800682 -0.268234 2.563948
C 2.112265 -0.426239 2.208122
N 2.193668 -0.277065 0.827199
H 0.382124 -0.316639 3.568274
H 2.996220 -0.633227 2.809529
C 3.431003 -0.385380 0.130029
C 4.260216 0.746391 0.032451
C 3.791931 -1.622740 -0.428940
C 5.479136 0.612227 -0.637762
C 5.018626 -1.710902 -1.094530
C 5.857437 -0.606186 -1.194195
H 6.143210 1.474131 -0.729698
H 5.322743 -2.660064 -1.541716
H 6.815185 -0.693821 -1.713424
C 3.789200 2.083399 0.568789
H 3.115847 1.871148 1.414412
C 2.841848 -2.801522 -0.372680
H 2.140421 -2.607783 0.453962
C 2.020511 -2.856744 -1.662717
H 1.499465 -1.899849 -1.815900
H 2.670725 -3.043126 -2.533093
H 1.272292 -3.665554 -1.618839
C 3.538895 -4.128093 -0.088770
H 4.201002 -4.433180 -0.914803
H 4.145914 -4.076930 0.827926
H 2.794773 -4.929815 0.039646
C 2.958956 2.796064 -0.502295
H 3.581288 3.039853 -1.378769
H 2.133275 2.147463 -0.833729
H 2.537758 3.736797 -0.110799
C 4.915884 2.970330 1.085412
H 5.536823 2.446750 1.828361
H 5.577230 3.313231 0.273647
H 4.500908 3.871427 1.562886
S -1.582202 0.235067 1.246289
C -1.849899 1.786752 0.245613
C -1.594047 1.629450 -1.250564
C -3.278158 2.279147 0.506944
C -0.857353 2.798425 0.836952
H -2.310795 0.914776 -1.682641
H -0.576651 1.228303 -1.396134
C -1.786389 2.993813 -1.926711
H -3.449088 2.376556 1.591864
H -4.017576 1.562414 0.122178
C -3.469537 3.634955 -0.187849
H 0.166793 2.432811 0.678304

H -1.011716 2.884557 1.926238
C -1.055905 4.156505 0.156681
H -1.604721 2.876268 -3.007371
C -3.219859 3.479469 -1.691339
C -0.798685 4.006120 -1.344363
H -4.503847 3.974458 -0.013885
C -2.484157 4.651038 0.392490
H -0.334719 4.869120 0.589167
H -3.939816 2.763774 -2.124281
H -3.383777 4.444172 -2.200035
H 0.235084 3.668218 -1.517812
H -0.912620 4.980661 -1.848309
H -2.631177 5.634852 -0.083687
H -2.668080 4.785257 1.471857
C -2.293147 -1.286499 0.417408
C -2.116000 -2.349492 1.515018
C -3.780839 -1.106340 0.118938
C -1.545359 -1.724348 -0.837492
H -2.630211 -2.024812 2.435342
H -1.045035 -2.450329 1.753412
C -2.688424 -3.684527 1.031981
H -3.924587 -0.350148 -0.667837
H -4.318549 -0.761640 1.018399
C -4.350805 -2.449590 -0.364180
H -0.469138 -1.791199 -0.621330
H -1.650826 -0.977084 -1.634181
C -2.120332 -3.063842 -1.314715
H -2.555302 -4.433514 1.829539
C -4.177682 -3.510603 0.724902
C -1.942942 -4.125532 -0.228828
H -5.422042 -2.314022 -0.586198
C -3.609587 -2.892682 -1.629193
H -1.577880 -3.367964 -2.224545
H -4.721535 -3.213143 1.637523
H -4.612313 -4.467086 0.389997
H -0.871575 -4.262706 -0.006897
H -2.328792 -5.098031 -0.578080
H -4.034936 -3.840203 -2.000199
H -3.744127 -2.146039 -2.430203

Coordinates of the optimized structure of **TS**$_{I \rightarrow IV}$

E = -1852.94395874
C -0.985110 -0.184775 0.426906
C 0.000226 -0.399100 1.389245
C -0.487991 -1.277442 2.404149
C -1.763918 -1.609763 2.011802
N -2.049271 -0.934265 0.839327
H 0.027133 -1.630012 3.295338
H -2.493279 -2.281861 2.459229
C -3.262705 -1.067183 0.103734
C -3.311000 -2.010344 -0.938172
C -4.355549 -0.247678 0.431149

C -4.498162 -2.115225 -1.667767
C -5.522482 -0.390195 -0.326212
C -5.593698 -1.312799 -1.364543
H -4.567761 -2.835462 -2.485780
H -6.388985 0.235170 -0.101624
H -6.514647 -1.407878 -1.945173
C -2.094259 -2.845068 -1.287555
H -1.446235 -2.858896 -0.396883
C -4.247764 0.799815 1.520988
H -3.430668 0.489792 2.190947
C -3.851677 2.149577 0.917042
H -2.874013 2.093407 0.413207
H -4.605956 2.487072 0.187704
H -3.774292 2.915485 1.705207
C -5.514866 0.913038 2.363531
H -6.359520 1.321403 1.786140
H -5.823029 -0.063382 2.767848
H -5.345718 1.595311 3.210548
C -1.301757 -2.181582 -2.416382
H -1.905474 -2.120189 -3.336187
H -1.001919 -1.161995 -2.132598
H -0.390616 -2.758150 -2.644813
C -2.439306 -4.292428 -1.625383
H -3.031698 -4.763329 -0.826394
H -3.012695 -4.372802 -2.562415
H -1.518763 -4.881275 -1.760028
S 1.484111 0.498348 1.342687
C 2.707607 -0.711224 0.613192
C 2.186611 -1.438778 -0.622737
C 4.026702 0.008558 0.324082
C 2.940128 -1.735500 1.736260
H 1.985092 -0.732580 -1.436865
H 1.232163 -1.932075 -0.380710
C 3.236350 -2.462614 -1.073983
H 4.379322 0.525635 1.232360
H 3.894290 0.772287 -0.455074
C 5.062674 -1.024860 -0.139889
H 1.991920 -2.242750 1.972527
H 3.278477 -1.217620 2.649124
C 3.983491 -2.764595 1.286561
H 2.862643 -2.972136 -1.977213
C 4.546717 -1.736299 -1.394670
C 3.473857 -3.486238 0.037359
H 6.003783 -0.499221 -0.369697
C 5.299183 -2.050464 0.970581
H 4.136949 -3.489596 2.102544
H 4.386524 -1.006632 -2.206408
H 5.301125 -2.455234 -1.755363
H 2.537448 -4.022779 0.264020
H 4.207733 -4.241715 -0.289830
H 6.062227 -2.781457 0.655208
H 5.687603 -1.550223 1.873668
C 1.126539 2.044855 0.138536

C 2.134091 3.083068 0.633555
C 1.366918 1.717821 -1.335285
C -0.298708 2.447024 0.338700
H 3.184492 2.762189 0.504443
H 1.970143 3.278477 1.704999
C 1.879162 4.364879 -0.184438
H 0.666123 0.934518 -1.658684
H 2.391506 1.362622 -1.519779
C 1.139489 3.000246 -2.148137
H -0.494177 2.636214 1.413238
H -0.973435 0.926541 -0.050155
C -0.530292 3.731001 -0.460077
H 2.581615 5.141810 0.160883
C 2.119286 4.075344 -1.669303
C 0.434859 4.837433 0.010056
H 1.324268 2.776558 -3.212844
C -0.298706 3.477506 -1.949557
H -1.570372 4.065979 -0.305316
H 3.159739 3.742248 -1.830941
H 1.983825 4.997513 -2.258798
H 0.250484 5.062283 1.073722
H 0.276208 5.776557 -0.551812
H -0.470715 4.396255 -2.537295
H -1.005375 2.714148 -2.312540

Coordinates of the optimized structure of the sulfide

E = -1536.25744791
C 0.258883 -0.032530 0.242059
C -0.608591 -0.084362 1.355226
C 0.093663 -0.088832 2.608492
C 1.412469 -0.041727 2.252391
N 1.478678 -0.012319 0.862601
H -0.313833 -0.121426 3.617529
H 2.314622 -0.025370 2.861729
C 2.721456 0.046725 0.166180
C 3.228694 1.299893 -0.215621
C 3.406477 -1.150974 -0.105873
C 4.455821 1.334828 -0.885341
C 4.631451 -1.068037 -0.773510
C 5.153553 0.163360 -1.158240
H 4.872115 2.295284 -1.197819
H 5.185854 -1.981180 -0.999556
H 6.113615 0.209177 -1.678520
C 2.433003 2.565559 0.029105
H 1.694167 2.336096 0.811954
C 2.783169 -2.487028 0.245849
H 2.121597 -2.317680 1.109868
C 1.901047 -2.960081 -0.912634
H 1.152601 -2.192407 -1.158015
H 2.509667 -3.150471 -1.811728
H 1.374139 -3.891558 -0.649968
C 3.800171 -3.550545 0.644294

H 4.435428 -3.853400 -0.203382
H 4.460188 -3.198816 1.451957
H 3.283040 -4.456153 0.996958
C 1.654575 2.935067 -1.235488
H 2.341576 3.194839 -2.057472
H 1.037433 2.084601 -1.560633
H 0.996465 3.800231 -1.053050
C 3.287594 3.727832 0.524844
H 3.858228 3.452456 1.424748
H 4.005615 4.068905 -0.237960
H 2.650016 4.589622 0.776406
S -2.325635 -0.113701 1.281124
C -2.823200 -1.445472 0.182396
C -4.021842 -2.094320 0.473937
C -2.032072 -1.802232 -0.906789
C -4.444788 -3.128320 -0.359895
H -4.614309 -1.801639 1.344274
C -2.466825 -2.841428 -1.725464
H -1.082417 -1.267789 -1.063415
C -3.668083 -3.499956 -1.456480
H -5.379824 -3.650333 -0.144725
H -1.857355 -3.139903 -2.581673
H -3.999874 -4.314247 -2.105060
C -2.873852 1.312294 0.330680
C -2.056582 1.866697 -0.649650
C -4.133495 1.830299 0.626875
C -2.526147 2.971810 -1.355266
H -1.065736 1.419974 -0.821887
C -4.590396 2.933525 -0.092151
H -4.748062 1.383033 1.412102
C -3.787579 3.502373 -1.080429
H -1.896259 3.421764 -2.126350
H -5.573617 3.354977 0.128713
H -4.147124 4.370343 -1.638205

Coordinates of the optimized structure of adamantene

E = -389.101497486
C -0.625692 -1.295523 1.026968
H -0.337875 -2.357386 0.958297
H -1.318825 -1.197202 1.875282
C -1.276003 -0.818249 -0.272483
H -2.166552 -1.419619 -0.522136
C 0.592120 -0.430138 1.234553
C 1.710746 -0.711787 0.276841
H 1.977818 -1.774204 0.137238
H 2.622500 -0.132925 0.491840
C -0.226611 -0.953606 -1.383662
H -0.639957 -0.606823 -2.345398
H 0.036272 -2.017014 -1.516956
C 1.025628 -0.135460 -1.036834
H 1.754107 -0.202138 -1.863002
C 0.583888 1.334676 -0.872456

H 1.453654 1.985993 -0.691393
H 0.123954 1.675475 -1.816373
C -1.681486 0.645253 -0.053668
H -2.437208 0.708036 0.745780
H -2.148368 1.053567 -0.965549
C 0.432530 0.915432 1.381252
H 1.366808 1.489122 1.475801
C -0.459862 1.490295 0.300921
H -0.737874 2.549770 0.427974

3.13 Energies and coordinates for the AYC with a $SOAd_2$ onium moiety

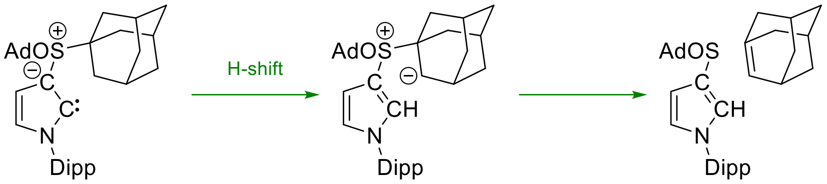

I III IV

	Corr(H)	Corr(G)	E(SCF)	E(PCM)	ΔG in hartree	ΔG in kJ/mol
**I**	0.822907	0.722681	-1928.15704903	-1928.16629837	0	0
**$TS_{I\rightarrow IV}$**	0.816758	0.716140	-1928.11341623	-1928.12073793	0.039019	102.445540
Adamantene	0.226862	0.188585	-389.101497486	-389.103682182	-0.000215	-0.563438
Sulfoxide	0.591509	0.505036	-1539.02371879	-1539.03377079		

Coordinates of the optimized structure of the AYC

E = -1928.15704903
C 1.046504 -0.009396 0.164084
C 0.159475 -0.030738 1.260050
C 0.801246 -0.307625 2.513163
C 2.116922 -0.465225 2.174233
N 2.231924 -0.283977 0.798375
H 0.350393 -0.368262 3.501042
H 2.987410 -0.686807 2.789776
C 3.484368 -0.385292 0.125808
C 4.314293 0.747802 0.057997
C 3.855863 -1.617045 -0.438164
C 5.546184 0.620577 -0.589514
C 5.095726 -1.698070 -1.079634
C 5.935588 -0.591904 -1.151045
H 6.211914 1.483291 -0.658703
H 5.409349 -2.642341 -1.530441
H 6.903466 -0.673945 -1.651984
C 3.834710 2.078757 0.601820
H 3.135124 1.857698 1.423492
C 2.904603 -2.796092 -0.414324
H 2.186332 -2.612415 0.400102
C 2.110425 -2.837437 -1.721850
H 1.594101 -1.878747 -1.879393
H 2.778625 -3.016439 -2.579947
H 1.360581 -3.645592 -1.701817

C 3.595391 -4.125990 -0.130822
H 4.274191 -4.421975 -0.946458
H 4.183374 -4.085146 0.798627
H 2.848675 -4.928697 -0.026514
C 3.041802 2.815326 -0.481331
H 3.691515 3.069137 -1.334711
H 2.221691 2.179486 -0.848766
H 2.615553 3.752297 -0.086625
C 4.951050 2.949077 1.167201
H 5.545635 2.408575 1.919434
H 5.638793 3.301054 0.381762
H 4.527527 3.844600 1.647470
C -1.784028 1.779420 0.109334
C -1.490236 1.623700 -1.380184
C -3.225595 2.240931 0.344071
C -0.818348 2.796287 0.736974
H -2.190591 0.905197 -1.832910
H -0.464862 1.238767 -1.510121
C -1.684316 2.992584 -2.048799
H -3.419931 2.315957 1.423983
H -3.942544 1.518745 -0.070271
C -3.415644 3.599439 -0.346150
H 0.215222 2.450959 0.597555
H -1.007424 2.863650 1.820394
C -1.020813 4.154839 0.059341
H -1.474761 2.884364 -3.125248
C -3.128932 3.458241 -1.844311
C -0.724538 4.014259 -1.435658
H -4.458840 3.920914 -0.193609
C -2.460981 4.626130 0.264772
H -0.320014 4.874614 0.512735
H -3.829397 2.737006 -2.299376
H -3.291487 4.424084 -2.351013
H 0.318066 3.691730 -1.585086
H -0.839451 4.988823 -1.938969
H -2.611859 5.611192 -0.207575
H -2.671916 4.747546 1.340277
C -2.228555 -1.313385 0.315483
C -2.034392 -2.362770 1.423456
C -3.718698 -1.135527 0.028507
C -1.481848 -1.751800 -0.942048
H -2.539896 -2.026740 2.340716
H -0.960757 -2.461345 1.650327
C -2.606277 -3.701415 0.949572
H -3.868921 -0.390423 -0.767591
H -4.238850 -0.780984 0.932185
C -4.286933 -2.484554 -0.438179
H -0.405524 -1.825925 -0.730948
H -1.591311 -1.010498 -1.744427
C -2.061797 -3.094594 -1.406147
H -2.462119 -4.443816 1.751141
C -4.099041 -3.536144 0.656866
C -1.871198 -4.148715 -0.315068

H -5.360460 -2.354325 -0.651412
C -3.554581 -2.930470 -1.707469
H -1.526567 -3.401534 -2.319192
H -4.633375 -3.232353 1.572472
H -4.533218 -4.496949 0.333954
H -0.797427 -4.282250 -0.102238
H -2.257422 -5.123847 -0.656002
H -3.976844 -3.882301 -2.070314
H -3.700302 -2.188924 -2.511375
S -1.513804 0.221159 1.095351
O -2.201055 0.400387 2.396142

Coordinates of the optimized structure of $\mathbf{TS}_{I\rightarrow IV}$

E = -1928.11341623
C -0.891654 -0.291801 0.221795
C 0.021755 -0.224142 1.278507
C -0.583399 -0.647477 2.498583
C -1.873573 -0.968196 2.156717
N -2.043959 -0.739242 0.801392
H -0.126160 -0.688044 3.483906
H -2.690987 -1.356027 2.761349
C -3.246335 -1.022802 0.088831
C -3.393770 -2.291189 -0.500173
C -4.234786 -0.029612 -0.001298
C -4.584313 -2.557438 -1.181102
C -5.412184 -0.345288 -0.686656
C -5.586807 -1.596058 -1.268372
H -4.730400 -3.531862 -1.651933
H -6.202991 0.403366 -0.770730
H -6.513050 -1.823345 -1.801791
C -2.269989 -3.307215 -0.447825
H -1.627471 -3.036848 0.404848
C -4.042626 1.330646 0.638442
H -2.966843 1.440834 0.845419
C -4.445909 2.472699 -0.290404
H -3.928111 2.401668 -1.258239
H -5.530230 2.483685 -0.484041
H -4.186855 3.441673 0.162944
C -4.782038 1.410994 1.974788
H -5.867979 1.288611 1.832479
H -4.443383 0.626806 2.667707
H -4.610541 2.386098 2.457103
C -1.414175 -3.206857 -1.712920
H -2.002229 -3.471777 -2.606353
H -1.033907 -2.183627 -1.847193
H -0.553008 -3.892443 -1.657370
C -2.760222 -4.733375 -0.217626
H -3.395850 -4.802318 0.678022
H -3.339928 -5.114174 -1.073250
H -1.904296 -5.412148 -0.081272
C 2.661475 -0.790400 0.334738
C 2.262380 -1.134928 -1.095889

C 4.109585 -0.297526 0.395702
C 2.503792 -2.020129 1.241185
H 2.370518 -0.254086 -1.744080
H 1.205099 -1.442117 -1.133258
C 3.181648 -2.258855 -1.593452
H 4.362476 -0.024880 1.431191
H 4.239267 0.600237 -0.224992
C 5.022913 -1.418466 -0.120130
H 1.456267 -2.358778 1.228345
H 2.755044 -1.745872 2.277865
C 3.424371 -3.133718 0.732975
H 2.895839 -2.508284 -2.627985
C 4.634388 -1.774255 -1.558517
C 3.030068 -3.492828 -0.701629
H 6.064250 -1.058850 -0.092377
C 4.876661 -2.653781 0.770062
H 3.302939 -4.012783 1.386567
H 4.756350 -0.895658 -2.214207
H 5.304747 -2.559040 -1.946697
H 1.988297 -3.854329 -0.729385
H 3.665724 -4.311978 -1.077115
H 5.550455 -3.453970 0.421138
H 5.170291 -2.413169 1.805317
C 1.227615 2.035801 0.017999
C 0.413296 2.880694 1.016696
C 2.545598 2.745280 -0.290618
C 0.422213 1.696908 -1.202712
H 0.995496 3.053589 1.935417
H -0.505861 2.342108 1.292961
C 0.055722 4.205936 0.343711
H 3.146640 2.140525 -0.986518
H 3.147734 2.942457 0.614484
C 2.162708 4.069645 -0.985999
H -0.636739 0.460150 -0.707658
H 1.065171 1.161344 -1.925068
C 0.042705 3.031672 -1.854617
H -0.527692 4.814345 1.055294
C 1.345936 4.939152 -0.027670
C -0.773404 3.911447 -0.906811
H 3.093656 4.593343 -1.260488
C 1.326439 3.795008 -2.242771
H -0.550021 2.834600 -2.764343
H 1.929995 5.162831 0.881494
H 1.110972 5.904465 -0.506519
H -1.703754 3.393690 -0.625721
H -1.057533 4.856991 -1.400817
H 1.092104 4.755066 -2.738907
H 1.908492 3.195457 -2.962952
S 1.567577 0.497164 1.094826
O 2.178498 0.845656 2.401153

Coordinates of the optimized structure of the sulfoxide

E = -1539.02371879
C 0.189866 -1.572204 0.186040
C -0.269816 -1.714724 1.522351
C -1.467701 -1.039834 1.596496
N -1.751000 -0.516018 0.357825
H 0.245233 -2.266015 2.305288
H -2.151902 -0.881806 2.427232
C -2.847923 0.342244 0.057018
C -4.092907 -0.223800 -0.263976
C -2.636200 1.732366 0.094042
C -5.149157 0.648395 -0.544261
C -3.720206 2.562472 -0.203647
C -4.965615 2.026747 -0.516332
H -6.131499 0.239721 -0.792941
H -3.587783 3.647031 -0.186555
H -5.802770 2.691747 -0.741940
C -4.297887 -1.724546 -0.293888
H -3.309560 -2.192707 -0.170904
C -1.279723 2.321789 0.428410
H -0.620499 1.490192 0.721131
C -0.657990 2.990823 -0.797895
H -0.578743 2.287533 -1.640405
H -1.261342 3.848496 -1.135736
H 0.353537 3.361033 -0.568782
C -1.351051 3.280643 1.615188
H -1.966753 4.165850 1.390023
H -1.780970 2.787843 2.499846
H -0.343717 3.637695 1.880243
C -4.864376 -2.194980 -1.632231
H -5.877056 -1.799706 -1.810162
H -4.227048 -1.875303 -2.470219
H -4.931032 -3.293522 -1.655246
C -5.170217 -2.178428 0.876891
H -4.731560 -1.879785 1.840714
H -6.179042 -1.739242 0.817672
H -5.278955 -3.274035 0.878580
C 2.805514 -0.762028 -0.272575
C 2.834552 -0.262234 1.166601
C 4.196414 -1.263463 -0.674269
C 2.360256 0.348499 -1.216329
H 3.110915 -1.097511 1.829547
H 1.829896 0.078500 1.465812
C 3.833817 0.895031 1.270118
H 4.168595 -1.656986 -1.705481
H 4.483093 -2.095639 -0.012430
C 5.194097 -0.105499 -0.574298
H 1.353523 0.699082 -0.939357
H 2.310983 -0.025173 -2.254277
C 3.359307 1.509456 -1.112840
H 3.853947 1.263642 2.309073
C 5.228711 0.409058 0.867258
C 3.392335 2.022487 0.330871
H 6.194406 -0.465329 -0.866235

C 4.755548 1.022971 -1.512761
H 3.038291 2.319975 -1.788356
H 5.559301 -0.393759 1.546953
H 5.958856 1.231035 0.958851
H 2.393644 2.388753 0.625714
H 4.084042 2.878151 0.410049
H 5.474192 1.858377 -1.464193
H 4.748135 0.666766 -2.556940
C -0.752418 -0.844546 -0.516445
H -0.793565 -0.531178 -1.556676
S 1.709047 -2.263089 -0.417282
O 2.192760 -3.201543 0.663078

Coordinates of the optimized structure of adamantene

E = -389.101497486
C -0.625692 -1.295523 1.026968
H -0.337875 -2.357386 0.958297
H -1.318825 -1.197202 1.875282
C -1.276003 -0.818249 -0.272483
H -2.166552 -1.419619 -0.522136
C 0.592120 -0.430138 1.234553
C 1.710746 -0.711787 0.276841
H 1.977818 -1.774204 0.137238
H 2.622500 -0.132925 0.491840
C -0.226611 -0.953606 -1.383662
H -0.639957 -0.606823 -2.345398
H 0.036272 -2.017014 -1.516956
C 1.025628 -0.135460 -1.036834
H 1.754107 -0.202138 -1.863002
C 0.583888 1.334676 -0.872456
H 1.453654 1.985993 -0.691393
H 0.123954 1.675475 -1.816373
C -1.681486 0.645253 -0.053668
H -2.437208 0.708036 0.745780
H -2.148368 1.053567 -0.965549
C 0.432530 0.915432 1.381252
H 1.366808 1.489122 1.475801
C -0.459862 1.490295 0.300921
H -0.737874 2.549770 0.427974

3.14 Energies and coordinates for the AYC with a PAd3 onium moiety

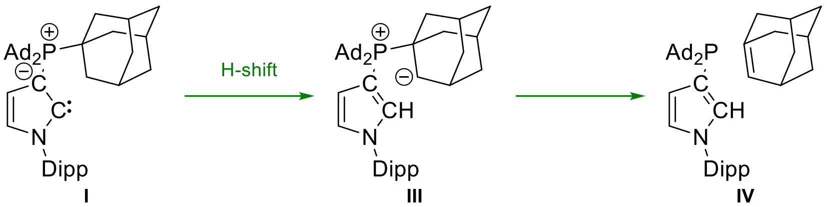

	Corr(H)	Corr(G)	E(SCF)	E(PCM)	ΔG in hartree	ΔG in kJ/mol
**I**	1.061852	0.949153	-2186.01017995	-2186.01839999	0	0

**TS**$_{I\rightarrow III}$	1.055792	0.943177	-2185.97060831	-2185.97751669	0.034907	91.649116
**III**	1.058160	0.944156	-2185.97114682	-2185.97865475	0.034748	91.231504
**TS**$_{III\rightarrow IV}$	1.057335	0.942887	-2185.96511335	-2185.97082832	0.041306	108.448037
Adamantene	0.226862	0.188585	-389.101497486	-389.103682182	0.014903	39.127979
Phosphine	0.829393	0.728528	-1796.86259708	-1796.86777475		

Coordinates of the optimized structure of **I**

E = -2186.01017995
C -1.625069 0.399896 -0.177704
C -0.422294 0.245328 -0.917829
C -0.719059 -0.088472 -2.295263
C -2.083816 -0.089191 -2.389084
N -2.589588 0.201926 -1.132271
H -0.044592 -0.290632 -3.123853
H -2.737435 -0.252445 -3.245097
C -3.987384 0.267244 -0.867299
C -4.639906 1.509913 -0.942165
C -4.675786 -0.916199 -0.546739
C -6.014422 1.547256 -0.689222
C -6.049844 -0.833199 -0.303594
C -6.714880 0.386719 -0.376699
H -6.546245 2.499958 -0.737867
H -6.609196 -1.736292 -0.050009
H -7.790033 0.433600 -0.185747
C -3.851473 2.774144 -1.216300
H -2.943420 2.473061 -1.761890
C -3.922822 -2.221873 -0.388420
H -3.019749 -2.148898 -1.014561
C -3.459010 -2.368740 1.062578
H -2.845482 -1.501920 1.350391
H -4.323614 -2.425277 1.743658
H -2.860858 -3.285317 1.193899
C -4.708344 -3.442975 -0.853168
H -5.578678 -3.645444 -0.208733
H -5.073523 -3.321764 -1.884469
H -4.070030 -4.339800 -0.820714
C -3.397732 3.391474 0.108763
H -4.266152 3.705776 0.710580
H -2.820364 2.654015 0.685926
H -2.763762 4.275855 -0.066489
C -4.601002 3.783199 -2.079629
H -4.956057 3.329675 -3.017721
H -5.474282 4.206327 -1.557832
H -3.941699 4.626721 -2.336840
C 1.435516 -1.850691 -0.062149
C 1.925411 -2.311635 -1.451885
C 2.452347 -2.319008 0.995137
C 0.081665 -2.561168 0.174993
H 2.891396 -1.852614 -1.701412
H 1.199001 -2.000895 -2.218072
C 2.094296 -3.835021 -1.496320
H 2.119653 -2.034669 2.002683

H 3.430902 -1.841711 0.835990
C 2.611541 -3.843739 0.941985
H -0.626905 -2.251878 -0.606922
H -0.365774 -2.257616 1.126891
C 0.256509 -4.081934 0.152243
H 2.458679 -4.111452 -2.499729
C 3.114224 -4.262859 -0.440105
C 0.753902 -4.515217 -1.225263
H 3.342517 -4.144195 1.710956
C 1.262359 -4.507967 1.223011
H -0.724074 -4.542611 0.357165
H 4.094043 -3.797940 -0.645268
H 3.264398 -5.354943 -0.476123
H 0.019998 -4.231127 -1.997548
H 0.866381 -5.611774 -1.270668
H 1.374000 -5.605402 1.228533
H 0.897961 -4.217639 2.223133
C 2.512431 0.945692 -0.954675
C 2.419523 2.459527 -0.664459
C 3.914369 0.449381 -0.548958
C 2.346602 0.802359 -2.485195
H 2.539354 2.658524 0.407350
H 1.421969 2.825494 -0.955912
C 3.511266 3.233317 -1.412474
H 4.028607 -0.618253 -0.782094
H 4.067869 0.554382 0.534094
C 4.997783 1.233930 -1.298324
H 1.372679 1.219398 -2.775299
H 2.354527 -0.249731 -2.793350
C 3.456090 1.551057 -3.231787
H 3.410776 4.301183 -1.157538
C 4.883193 2.722837 -0.968129
C 3.355642 3.042113 -2.919200
H 5.983457 0.856698 -0.979187
C 4.826073 1.023687 -2.803454
H 3.314114 1.385302 -4.312420
H 5.016811 2.882523 0.115577
H 5.683017 3.287668 -1.475744
H 2.381569 3.435010 -3.254647
H 4.135678 3.604602 -3.459650
H 5.624996 1.546682 -3.355462
H 4.916460 -0.048419 -3.049072
C 0.913008 0.682384 1.716855
C 0.190717 2.052164 1.697005
C 0.016967 -0.270209 2.542947
C 2.267720 0.832234 2.439382
H -0.785482 1.896890 1.211444
H 0.743993 2.791789 1.108600
C 0.010695 2.584550 3.121293
H 0.467437 -1.269421 2.611277
H -0.950196 -0.349918 2.022014
C -0.178469 0.260818 3.965866
H 2.921169 1.536478 1.906105

H 2.802012 -0.128282 2.483599
C 2.052446 1.361465 3.863958
H -0.481429 3.569030 3.055159
C -0.868136 1.622460 3.917818
C 1.371256 2.730047 3.804709
H -0.810091 -0.458927 4.512594
C 1.179324 0.388399 4.656801
H 3.039342 1.456109 4.347318
H -1.854185 1.528660 3.435588
H -1.033416 2.005143 4.939695
H 2.003045 3.444496 3.248778
H 1.249983 3.135488 4.823700
H 1.053195 0.750232 5.691348
H 1.670169 -0.598407 4.719702
P 1.100142 0.030293 -0.061268

Coordinates of the optimized structure of the **TS**$_{I \rightarrow III}$

E = -2185.97060831
C -1.619842 0.078010 -0.251765
C -0.400513 0.066227 -0.923137
C -0.673081 0.085692 -2.335811
C -2.040070 0.110189 -2.466519
N -2.592189 0.107649 -1.200344
H 0.031769 0.077048 -3.164056
H -2.670430 0.115921 -3.353791
C -3.986375 0.032620 -0.912752
C -4.732037 1.221106 -0.843681
C -4.551739 -1.232430 -0.682172
C -6.094771 1.115583 -0.552728
C -5.917568 -1.287512 -0.388629
C -6.681695 -0.126677 -0.330251
H -6.706045 2.018026 -0.491716
H -6.390581 -2.253770 -0.201381
H -7.748633 -0.189683 -0.102381
C -4.046722 2.564625 -0.993564
H -3.172351 2.411133 -1.645918
C -3.691140 -2.480629 -0.690410
H -2.792127 -2.249797 -1.283959
C -3.227106 -2.816594 0.730217
H -2.663536 -1.988127 1.186346
H -4.090140 -3.039773 1.378232
H -2.574994 -3.705153 0.720987
C -4.378285 -3.673811 -1.347995
H -5.238117 -4.030831 -0.759261
H -4.739274 -3.428105 -2.358330
H -3.675049 -4.516731 -1.432243
C -3.521515 3.031406 0.367652
H -4.354729 3.202009 1.068075
H -2.853720 2.281553 0.816171
H -2.961084 3.974562 0.267624
C -4.925350 3.627996 -1.642694
H -5.337390 3.283724 -2.603499

H -5.769416 3.915706 -0.996066
H -4.339742 4.540961 -1.830074
C 2.274605 1.282616 -0.824849
C 1.448494 2.550555 -1.150088
C 3.456806 1.664838 0.088674
C 2.854761 0.775576 -2.163332
H 0.981013 2.957353 -0.246816
H 0.626538 2.288742 -1.832102
C 2.335658 3.622200 -1.791607
H 4.068528 0.779174 0.317588
H 3.091472 2.051969 1.048540
C 4.327982 2.735309 -0.577853
H 2.041722 0.500805 -2.850263
H 3.465902 -0.122774 -2.005883
C 3.730961 1.846259 -2.824933
H 1.715878 4.514051 -1.980511
C 3.484009 3.982435 -0.846909
C 2.899453 3.095654 -3.110162
H 5.154507 2.985594 0.107404
C 4.891066 2.199537 -1.894042
H 4.122958 1.433266 -3.769247
H 3.085303 4.381767 0.100977
H 4.108773 4.775336 -1.291496
H 2.077095 2.856191 -3.805110
H 3.521953 3.863922 -3.599092
H 5.542875 2.953256 -2.366776
H 5.514569 1.308629 -1.706369
C 0.739799 0.392167 1.831400
C 1.946111 0.064225 2.742623
C 0.455088 1.910969 1.923616
C -0.536297 -0.336569 2.289026
H 2.880089 0.573084 2.444871
H 2.145573 -1.017448 2.721514
C 1.596565 0.467154 4.185818
H -0.389746 2.164799 1.265328
H 1.323062 2.507666 1.607562
C 0.128027 2.309231 3.364913
H -0.365218 -1.423526 2.289187
H -1.586182 -0.028622 0.945308
C -0.828528 0.058060 3.737193
H 2.459190 0.218889 4.827914
C 1.327456 1.971899 4.252413
C 0.351916 -0.284439 4.664937
H -0.054062 3.397913 3.386584
C -1.109371 1.555744 3.843934
H -1.720731 -0.498838 4.072784
H 2.217867 2.536574 3.921796
H 1.126215 2.272517 5.294554
H 0.535127 -1.372345 4.642063
H 0.142685 -0.013915 5.717835
H -1.346733 1.849248 4.882421
H -1.977191 1.816616 3.216915
C 1.815222 -1.751468 -0.233524

C 1.003152 -2.711172 0.659711
C 1.613174 -2.228165 -1.689306
C 3.303422 -1.870394 0.145321
H -0.067527 -2.627221 0.414924
H 1.103559 -2.426459 1.714110
C 1.482564 -4.155072 0.488999
H 2.140851 -1.582862 -2.401542
H 0.543491 -2.177280 -1.941118
C 2.115467 -3.666265 -1.859387
H 3.463190 -1.548153 1.184528
H 3.922655 -1.219694 -0.489064
C 3.775292 -3.319894 -0.026017
H 0.881992 -4.796833 1.154308
C 1.305245 -4.598895 -0.961223
C 2.958898 -4.243068 0.880131
H 1.982968 -3.954966 -2.915264
C 3.597281 -3.744995 -1.484997
H 4.841362 -3.373810 0.250277
H 0.239086 -4.562973 -1.241674
H 1.639189 -5.642649 -1.088672
H 3.089391 -3.951327 1.935897
H 3.319209 -5.281433 0.786510
H 3.971731 -4.772331 -1.629521
H 4.192445 -3.091899 -2.146540
P 1.125298 -0.000748 -0.008962

Coordinates of the optimized structure of the **III**

E = -2185.97114682
C 1.634000 -0.130280 -0.241214
C 0.411570 -0.097247 -0.901543
C 0.688205 -0.075361 -2.313236
C 2.054766 -0.108435 -2.447596
N 2.609662 -0.146600 -1.185002
H -0.016121 -0.035179 -3.140342
H 2.683903 -0.097034 -3.335345
C 4.003581 -0.082514 -0.892984
C 4.746293 -1.274324 -0.857795
C 4.569882 1.174462 -0.624327
C 6.109392 -1.178722 -0.565480
C 5.936134 1.219061 -0.331021
C 6.698463 0.055915 -0.308355
H 6.719306 -2.083380 -0.530437
H 6.410390 2.178708 -0.114743
H 7.765742 0.110588 -0.080078
C 4.058044 -2.611783 -1.044034
H 3.184768 -2.439466 -1.693146
C 3.711829 2.423973 -0.588608
H 2.796538 2.206581 -1.161886
C 3.288064 2.734277 0.850173
H 2.742975 1.894227 1.307009
H 4.168238 2.948472 1.477674
H 2.632698 3.619924 0.876283

C 4.383859 3.627475 -1.243105
H 5.263629 3.968654 -0.674911
H 4.711671 3.399982 -2.268796
H 3.682296 4.474830 -1.288918
C 3.530834 -3.115420 0.303555
H 4.363432 -3.304242 0.999999
H 2.859634 -2.382703 0.775009
H 2.973130 -4.056767 0.176150
C 4.935631 -3.658546 -1.721061
H 5.349954 -3.288957 -2.671439
H 5.777844 -3.965871 -1.081113
H 4.348279 -4.564611 -1.934245
C -2.360555 -1.085785 -0.969603
C -1.645822 -2.386131 -1.409933
C -3.588802 -1.451558 -0.111151
C -2.871643 -0.397880 -2.254850
H -1.236425 -2.919850 -0.545246
H -0.789205 -2.139179 -2.052980
C -2.612332 -3.304035 -2.165597
H -4.122996 -0.542545 0.203968
H -3.275545 -1.966807 0.805843
C -4.540521 -2.366083 -0.890127
H -2.028543 -0.125084 -2.904717
H -3.405474 0.530296 -2.012288
C -3.825530 -1.315650 -3.029621
H -2.070504 -4.224827 -2.437251
C -3.806522 -3.651681 -1.274978
C -3.101365 -2.599926 -3.430154
H -5.398127 -2.606832 -0.240608
C -5.029657 -1.653939 -2.150954
H -4.161360 -0.777845 -3.931824
H -3.463836 -4.178202 -0.368251
H -4.490340 -4.336233 -1.804566
H -2.247689 -2.367137 -4.088806
H -3.779080 -3.256965 -4.000925
H -5.736694 -2.295315 -2.703381
H -5.575111 -0.733569 -1.880729
C -0.823139 -0.603697 1.805179
C -2.034548 -0.286281 2.714125
C -0.652345 -2.139941 1.730672
C 0.478117 -0.020050 2.368034
H -2.995777 -0.684840 2.343271
H -2.150345 0.803925 2.808429
C -1.759284 -0.871469 4.111203
H 0.193053 -2.380181 1.067249
H -1.551892 -2.631148 1.331561
C -0.399903 -2.717776 3.124996
H 0.367510 1.066914 2.502028
H 1.663078 -0.143145 0.907695
C 0.701998 -0.598069 3.766376
H -2.621847 -0.632368 4.756965
C -1.600918 -2.390556 4.014509
C -0.479083 -0.275211 4.702294

H -0.297403 -3.813018 3.029981
C 0.872345 -2.114718 3.712859
H 1.620483 -0.150197 4.184531
H -2.518015 -2.847482 3.600466
H -1.456461 -2.818106 5.021150
H -0.581836 0.819065 4.799896
H -0.324460 -0.677781 5.722310
H 1.055858 -2.536740 4.717557
H 1.737885 -2.368584 3.079901
C -1.650880 1.818556 -0.064952
C -0.788903 2.610712 0.937723
C -1.362998 2.411185 -1.462358
C -3.133329 2.034258 0.293792
H 0.277899 2.452188 0.716435
H -0.952368 2.236571 1.955733
C -1.131373 4.102190 0.896010
H -1.921799 1.885669 -2.245793
H -0.294591 2.287933 -1.694503
C -1.732015 3.898071 -1.502611
H -3.348012 1.632265 1.294806
H -3.790329 1.503748 -0.410360
C -3.471131 3.530235 0.252070
H -0.496909 4.620377 1.633711
C -0.870091 4.662982 -0.500035
C -2.605493 4.287706 1.260322
H -1.542904 4.273260 -2.522071
C -3.212055 4.072991 -1.154773
H -4.536408 3.653559 0.508485
H 0.196955 4.555472 -0.758927
H -1.104773 5.740508 -0.532928
H -2.793698 3.912095 2.280185
H -2.870046 5.358629 1.257751
H -3.491637 5.138634 -1.207595
H -3.840955 3.540878 -1.889457
P -1.124793 -0.001431 0.000704

Coordinates of the optimized structure of $\mathbf{TS}_{III \rightarrow IV}$

E = -2185.96511335
C 1.545474 -0.116869 -0.353176
C 0.314606 -0.023171 -0.989620
C 0.585860 0.118677 -2.393042
C 1.951801 0.089810 -2.545474
N 2.520752 -0.057818 -1.300445
H -0.131704 0.235350 -3.201409
H 2.574674 0.172899 -3.433313
C 3.918336 -0.035020 -1.023364
C 4.652482 -1.228812 -1.134072
C 4.503117 1.180392 -0.631527
C 6.021525 -1.174679 -0.860908
C 5.875271 1.183143 -0.362095
C 6.627501 0.020025 -0.482025
H 6.623531 -2.082031 -0.937887

H 6.361358 2.110947 -0.051798
H 7.699459 0.041648 -0.270921
C 3.952578 -2.534833 -1.453305
H 3.062597 -2.288674 -2.053660
C 3.667916 2.431570 -0.445309
H 2.693706 2.250675 -0.925896
C 3.407581 2.673278 1.043564
H 2.921586 1.804971 1.513134
H 4.350366 2.862325 1.581723
H 2.755229 3.549144 1.187792
C 4.283701 3.658089 -1.113116
H 5.235870 3.949674 -0.642397
H 4.476596 3.479473 -2.181650
H 3.602601 4.519231 -1.028747
C 3.460418 -3.188626 -0.158223
H 4.310269 -3.455813 0.490196
H 2.803737 -2.514384 0.410568
H 2.895625 -4.108764 -0.376461
C 4.806600 -3.498340 -2.269857
H 5.196473 -3.022631 -3.182596
H 5.664502 -3.878934 -1.693230
H 4.208993 -4.372601 -2.569531
C -2.552121 -0.728980 -1.159418
C -1.984874 -2.019140 -1.790386
C -3.759793 -1.112336 -0.278104
C -3.055238 0.177752 -2.297798
H -1.606249 -2.691380 -1.007909
H -1.125284 -1.779365 -2.432397
C -3.062012 -2.734385 -2.612079
H -4.189114 -0.216926 0.196843
H -3.432352 -1.772838 0.538685
C -4.832692 -1.823185 -1.108873
H -2.220953 0.475403 -2.950421
H -3.486665 1.102454 -1.888414
C -4.126230 -0.541084 -3.128663
H -2.624082 -3.650893 -3.041001
C -4.243650 -3.099842 -1.711626
C -3.538917 -1.814977 -3.737249
H -5.678658 -2.078250 -0.449271
C -5.311269 -0.898904 -2.229746
H -4.460218 0.136827 -3.932006
H -3.912355 -3.779915 -0.908616
H -5.013011 -3.637747 -2.291154
H -2.696688 -1.564373 -4.404413
H -4.296888 -2.325952 -4.354913
H -6.101324 -1.392817 -2.820309
H -5.754952 0.017171 -1.803547
C -0.712511 -1.022638 1.933583
C -1.861201 -0.759038 2.883553
C -0.724427 -2.476020 1.461217
C 0.554669 -0.606876 2.476180
H -2.862091 -1.016167 2.485450
H -1.879681 0.295208 3.200699

C -1.533460 -1.657202 4.115732
H 0.039498 -2.621034 0.681561
H -1.702443 -2.742628 1.032779
C -0.448531 -3.382169 2.660081
H 0.481220 0.403650 2.913985
H 1.707988 -0.261497 0.730747
C 0.938470 -1.555098 3.616824
H -2.309380 -1.483015 4.880523
C -1.543859 -3.132582 3.701124
C -0.149103 -1.337598 4.701201
H -0.472269 -4.438002 2.338695
C 0.928489 -3.027602 3.218494
H 1.928460 -1.298153 4.033097
H -2.528489 -3.408218 3.283647
H -1.382333 -3.770037 4.586821
H -0.114100 -0.291755 5.048406
H 0.024356 -1.976608 5.588452
H 1.160597 -3.662666 4.091265
H 1.702208 -3.221480 2.458216
C -1.510283 1.875677 0.203674
C -0.509348 2.334966 1.280970
C -1.236565 2.703622 -1.066084
C -2.931200 2.168128 0.714572
H 0.517763 2.109193 0.955469
H -0.672698 1.771725 2.209390
C -0.661958 3.830506 1.566601
H -1.912086 2.412527 -1.881181
H -0.211051 2.507179 -1.415592
C -1.412724 4.198557 -0.776278
H -3.134330 1.583550 1.626726
H -3.682825 1.869006 -0.032000
C -3.086972 3.666835 1.003209
H 0.070126 4.115362 2.340521
C -0.408342 4.632977 0.290855
C -2.081027 4.099109 2.072050
H -1.233968 4.761087 -1.707932
C -2.837684 4.460627 -0.281902
H -4.113309 3.852307 1.361464
H 0.620913 4.460359 -0.067777
H -0.503713 5.713798 0.491513
H -2.260025 3.544208 3.008545
H -2.210605 5.170177 2.302739
H -2.984038 5.538042 -0.095508
H -3.567432 4.168088 -1.056608
P -1.210042 0.025613 -0.045138

Coordinates of the optimized structure of the phosphine

E = -1796.86259708
C -0.167745 0.483472 0.017620
C -0.422474 0.712724 1.410020
C -1.785656 0.796625 1.578670
N -2.381362 0.633872 0.351231

H 0.313804 0.800089 2.205160
H -2.392495 0.949092 2.468808
C -3.778991 0.479025 0.127407
C -4.314603 -0.820641 0.152862
C -4.566957 1.616830 -0.116898
C -5.684454 -0.963953 -0.086552
C -5.931528 1.422938 -0.348854
C -6.484848 0.145963 -0.335142
H -6.131113 -1.961294 -0.077059
H -6.572398 2.284973 -0.545818
H -7.553913 0.015347 -0.519904
C -3.440851 -2.032834 0.411467
H -2.433602 -1.667277 0.663263
C -3.942421 2.995961 -0.181860
H -2.978836 2.939730 0.347724
C -3.642251 3.373115 -1.634211
H -2.976506 2.636751 -2.107607
H -4.569526 3.421868 -2.227574
H -3.150722 4.357098 -1.687950
C -4.785972 4.064159 0.507647
H -5.735377 4.245494 -0.020597
H -5.025359 3.782270 1.544076
H -4.242206 5.020840 0.531493
C -3.312264 -2.894007 -0.845158
H -4.287102 -3.306595 -1.151007
H -2.915072 -2.310458 -1.688946
H -2.629056 -3.739394 -0.667726
C -3.936337 -2.848772 1.603724
H -4.007492 -2.228016 2.509357
H -4.929800 -3.286463 1.416470
H -3.243739 -3.678878 1.813425
C 2.628961 1.319099 -0.261556
C 3.190507 1.063769 1.144119
C 3.804006 1.334663 -1.262626
C 1.976849 2.716733 -0.287342
H 3.682900 0.079537 1.182960
H 2.377426 1.044621 1.885776
C 4.206743 2.149741 1.521300
H 3.413450 1.517863 -2.277636
H 4.297298 0.350421 -1.289119
C 4.827096 2.410294 -0.884909
H 1.127268 2.747030 0.412631
H 1.562399 2.911090 -1.292303
C 2.995409 3.797453 0.085825
H 4.589740 1.945137 2.535423
C 5.364902 2.131821 0.520945
C 3.528379 3.521397 1.494151
H 5.656782 2.387588 -1.611305
C 4.154626 3.784024 -0.912329
H 2.497166 4.781294 0.064334
H 5.871475 1.151637 0.546042
H 6.118374 2.889042 0.797652
H 2.700939 3.549460 2.223673

H 4.245555 4.305659 1.791357
H 4.884885 4.571973 -0.659800
H 3.782758 4.002947 -1.927598
C 1.689088 -1.647759 -0.387135
C 0.644368 -2.490731 -1.149466
C 3.085932 -2.085863 -0.855533
C 1.540595 -1.955592 1.110040
H 0.720782 -2.281425 -2.229980
H -0.368645 -2.186900 -0.837702
C 0.846173 -3.984394 -0.886075
H 3.862753 -1.516103 -0.320490
H 3.207754 -1.865063 -1.930324
C 3.293127 -3.582617 -0.593824
H 0.535329 -1.661560 1.449349
H 2.261541 -1.371386 1.700520
C 1.757201 -3.450937 1.374354
H 0.083184 -4.554152 -1.443496
C 2.243559 -4.393348 -1.357618
C 0.707016 -4.261390 0.612143
H 4.302553 -3.869320 -0.933711
C 3.156824 -3.857722 0.906117
H 1.658524 -3.641362 2.456455
H 2.343110 -4.216296 -2.441998
H 2.403268 -5.472553 -1.191573
H -0.305086 -3.984681 0.955723
H 0.832962 -5.338869 0.815047
H 3.333087 -4.927174 1.113397
H 3.921868 -3.292635 1.465971
P 1.333399 0.129360 -0.948076
C -1.410912 0.450790 -0.593666
H -1.670931 0.291286 -1.638288

Coordinates of the optimized structure of adamantene

E = -389.101497486
C -0.625692 -1.295523 1.026968
H -0.337875 -2.357386 0.958297
H -1.318825 -1.197202 1.875282
C -1.276003 -0.818249 -0.272483
H -2.166552 -1.419619 -0.522136
C 0.592120 -0.430138 1.234553
C 1.710746 -0.711787 0.276841
H 1.977818 -1.774204 0.137238
H 2.622500 -0.132925 0.491840
C -0.226611 -0.953606 -1.383662
H -0.639957 -0.606823 -2.345398
H 0.036272 -2.017014 -1.516956
C 1.025628 -0.135460 -1.036834
H 1.754107 -0.202138 -1.863002
C 0.583888 1.334676 -0.872456
H 1.453654 1.985993 -0.691393
H 0.123954 1.675475 -1.816373
C -1.681486 0.645253 -0.053668

H -2.437208 0.708036 0.745780
H -2.148368 1.053567 -0.965549
C 0.432530 0.915432 1.381252
H 1.366808 1.489122 1.475801
C -0.459862 1.490295 0.300921
H -0.737874 2.549770 0.427974

3.15 Energies and coordinates for the AYC with a S(CH2)2 onium moiety

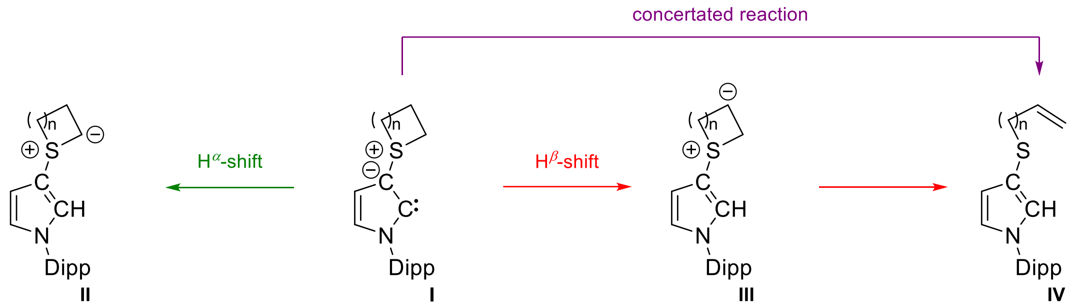

n		Corr(H)	Corr(G)	E(SCF)	E(PCM)	ΔG in hartree	ΔG in kJ/mol
0	**I**	0.388206	0.317162	-1151.85936849	-1151.86955030	0	0
	**TS**I→III	0.383556	0.313313	-1151.84583565	-1151.85122640	0.014475	38.003850
	**III**	0.388206	0.317166	-1151.85936829	-1151.88430746	-0.018523	-48.632557
	**TS**III→IV	0.387146	0.312220	-1151.87246797	-1151.88000486	-0.015397	-40.423668
	Sulfide	0.389568	0.312896	-1151.96440571	-1151.97002062	-0.104736	-274.985208
	TSconc	n.d.	n.d.	n.d.	n.d.	-	-
1	**I**	0.418951	0.344347	-1191.14985066	-1191.15975495	0	0
	**TS**I→II	0.413822	0.340489	-1191.12601054	-1191.13094039	0.024957	65.523448
	**II**	0.418435	0.342466	-1191.15558893	-1191.16241539	-0.004541	-11.923551
	**TS**I→III	0.412483	0.338735	-1191.10898170	-1191.11416939	0.039974	104.950582
	**III**	0.415742	0.340728	-1191.11992466	-1191.12740644	0.028730	75.429329
	**TS**III→IV	0.414687	0.340998	-1191.11875762	-1191.12478010	0.031626	83.033669
	Sulfide	0.419318	0.341866	-1191.24247295	-1191.24821195	-0.090938	-238.757719
	**TS**I→IV	n.d.	n.d.	n.d.	n.d.	-	-
2	**I**	0.449371	0.373620	-1230.45749380	-1230.46755866	0	0
	**TS**I→II	0.444316	0.369073	-1230.43563018	-1230.44141073	0.021601	56.713242
	**II**	0.448933	0.368408	-1230.46940683	-1230.47515630	-0.012810	-33.631710
	**TS**I→III	n.d.	n.d.	n.d.	n.d.	-	-
	**III**	n.d.	n.d.	n.d.	n.d.	-	-
	**TS**III→IV	n.d.	n.d.	n.d.	n.d.	-	-
	Sulfide	0.449378	0.367264	-1230.52367161	-1230.52976916	-0.068566	-180.021346
	**TS**I→IV	0.442867	0.367717	-1230.42223987	-1230.42718359	0.034472	90.506420

Coordinates of the optimized structure of **I** for n = 0

E = -1151.85936849
C -1.189466 0.228249 -0.220418
C -2.228135 0.398267 0.703414
C -1.782424 0.387229 2.064199
C -0.425829 0.219908 1.962699
N -0.105886 0.126559 0.614039

H -2.358588 0.497722 2.981661
H 0.342014 0.163897 2.733192
C 1.232043 -0.070594 0.160380
C 2.040861 1.052218 -0.085680
C 1.702180 -1.383590 -0.015731
C 3.349721 0.832889 -0.525344
C 3.017349 -1.555283 -0.457487
C 3.834449 -0.457974 -0.709250
H 4.000991 1.685715 -0.727443
H 3.409703 -2.563436 -0.606236
H 4.861071 -0.610579 -1.051715
C 1.477754 2.451753 0.057461
H 0.664226 2.396858 0.798027
C 0.781274 -2.566706 0.205950
H 0.014234 -2.244544 0.927927
C 0.059259 -2.911632 -1.099098
H -0.487662 -2.032505 -1.472305
H 0.779506 -3.227979 -1.871239
H -0.656314 -3.735961 -0.943515
C 1.487074 -3.783740 0.794375
H 2.201983 -4.230440 0.085074
H 2.037849 -3.528694 1.712606
H 0.752465 -4.565058 1.043634
C 0.853350 2.894571 -1.268133
H 1.618663 2.947758 -2.059703
H 0.077352 2.179705 -1.580396
H 0.393777 3.891354 -1.169231
C 2.496084 3.467572 0.563958
H 2.975515 3.132284 1.496402
H 3.290991 3.659485 -0.174368
H 2.003153 4.431871 0.761818
C -4.171977 -0.814521 -0.985704
C -3.749787 0.465411 -1.588732
H -3.397799 -1.581661 -0.870597
H -5.210902 -1.149605 -1.070873
H -4.471478 1.087442 -2.124779
H -2.667870 0.544272 -1.824773
S -3.883397 0.552627 0.210463

Coordinates of the optimized structure of **TS**$_{I\rightarrow III}$ for n = 0

E = -1151.84583565
C -1.235629 0.285432 -0.127911
C -2.266131 0.475323 0.774963
C -1.800702 0.454772 2.118206
C -0.441939 0.259844 2.003789
N -0.125407 0.158233 0.659750
H -2.360953 0.577443 3.043311
H 0.330437 0.183275 2.768186
C 1.193688 -0.086140 0.176409
C 2.032218 1.008815 -0.094205
C 1.609423 -1.415364 -0.011643
C 3.319166 0.742599 -0.570660

C 2.904358 -1.633035 -0.491175
C 3.751660 -0.564992 -0.767228
H 3.994306 1.571581 -0.792455
H 3.255982 -2.654541 -0.650894
H 4.761631 -0.753743 -1.139603
C 1.524454 2.427755 0.064647
H 0.715282 2.399338 0.811180
C 0.657231 -2.567331 0.239482
H -0.113439 -2.202717 0.936451
C -0.050811 -2.952848 -1.061581
H -0.575780 -2.086353 -1.489587
H 0.673139 -3.317767 -1.808179
H -0.787254 -3.753140 -0.883158
C 1.330188 -3.773499 0.887100
H 2.053127 -4.256540 0.210705
H 1.865008 -3.492139 1.806863
H 0.577885 -4.533303 1.149309
C 0.910832 2.911599 -1.251675
H 1.671330 2.940060 -2.048869
H 0.100326 2.241665 -1.573737
H 0.496083 3.925937 -1.138718
C 2.587151 3.396562 0.572791
H 3.058183 3.032829 1.498657
H 3.384228 3.562334 -0.169356
H 2.135491 4.378136 0.783039
C -4.018369 -0.774703 -1.052919
C -3.352791 0.391022 -1.646644
H -3.371084 -1.579210 -0.674604
H -5.047086 -1.070196 -1.301853
H -3.921123 1.009044 -2.351746
H -1.997501 0.351259 -1.340192
S -3.871522 0.681516 0.072908

Coordinates of the optimized structure of **III** for n = 0

E = -1151.85936829
C -1.189422 0.228485 -0.220305
C -2.228140 0.398063 0.703589
C -1.782407 0.386748 2.064353
C -0.425797 0.219489 1.962808
N -0.105835 0.126710 0.614106
H -2.358574 0.496983 2.981846
H 0.342096 0.163596 2.733257
C 1.232067 -0.070627 0.160382
C 2.041010 1.052063 -0.085694
C 1.702003 -1.383706 -0.015741
C 3.349857 0.832541 -0.525351
C 3.017138 -1.555592 -0.457481
C 3.834399 -0.458389 -0.709227
H 4.001272 1.685257 -0.727428
H 3.409363 -2.563803 -0.606192
H 4.861006 -0.611145 -1.051674
C 1.478085 2.451681 0.057573

H 0.664619 2.396804 0.798212
C 0.780885 -2.566642 0.205936
H 0.014215 -2.244531 0.928332
C 0.058278 -2.910953 -1.098947
H -0.488554 -2.031557 -1.471661
H 0.778144 -3.227248 -1.871468
H -0.657443 -3.735145 -0.943337
C 1.486621 -3.784042 0.793675
H 2.201160 -4.230670 0.083951
H 2.037797 -3.529448 1.711792
H 0.751916 -4.565267 1.042932
C 0.853579 2.894573 -1.267948
H 1.618755 2.947555 -2.059666
H 0.077421 2.179804 -1.580049
H 0.394189 3.891443 -1.169054
C 2.496567 3.467349 0.564019
H 2.975879 3.132019 1.496511
H 3.291554 3.659050 -0.174266
H 2.003807 4.431754 0.761783
C -4.171856 -0.813885 -0.985949
C -3.749316 0.466023 -1.588831
H -3.397961 -1.581329 -0.870975
H -5.210827 -1.148778 -1.071410
H -4.470925 1.088266 -2.124730
H -2.667326 0.544871 -1.824538
S -3.883252 0.552842 0.210495

Coordinates of the optimized structure of $\mathbf{TS}_{III\rightarrow IV}$ for n = 0

E = -1151.87246797
C 1.122528 -0.418648 -0.435401
C 2.247728 -0.748033 0.296259
C 1.867787 -0.789238 1.668014
C 0.528393 -0.470437 1.719834
N 0.087250 -0.242600 0.439980
H 2.511235 -1.022351 2.513963
H -0.155753 -0.376912 2.560675
C -1.237969 0.138735 0.079212
C -2.191085 -0.865171 -0.161084
C -1.539591 1.508128 -0.027171
C -3.482136 -0.463293 -0.516830
C -2.841627 1.859514 -0.393368
C -3.804354 0.884307 -0.634405
H -4.247003 -1.220089 -0.707340
H -3.107102 2.914749 -0.490147
H -4.817877 1.178908 -0.917152
C -1.847927 -2.335239 -0.031460
H -0.769372 -2.405047 0.174918
C -0.484392 2.569583 0.211787
H 0.376013 2.074719 0.687741
C -0.000063 3.155847 -1.115411
H 0.402066 2.372601 -1.774909
H -0.821900 3.657130 -1.651481

H 0.795934 3.897691 -0.946164
C -0.966011 3.662319 1.163370
H -1.791724 4.249560 0.731603
H -1.317213 3.238023 2.115884
H -0.147483 4.364483 1.384114
C -2.114781 -3.093461 -1.330680
H -3.185309 -3.094022 -1.590434
H -1.565423 -2.646929 -2.172943
H -1.796879 -4.143146 -1.235616
C -2.582963 -2.966002 1.151274
H -2.350791 -2.441131 2.090084
H -3.675153 -2.932448 1.009975
H -2.292211 -4.021215 1.270727
C 4.668795 0.578711 0.099783
C 4.575766 0.223034 -1.268921
H 4.060838 1.358610 0.597946
H 5.517768 0.232041 0.701283
H 3.894759 0.791719 -1.914987
H 0.982963 -0.295367 -1.507024
S 3.836267 -1.163581 -0.428712

Coordinates of the optimized structure of the sulfide for n = 0

E = -1151.96440571
C 1.018878 -0.411881 -0.481678
C 2.175369 -0.699969 0.221746
C 1.850177 -0.656350 1.609042
C 0.514131 -0.336003 1.696328
N 0.018333 -0.195980 0.422518
H 2.531475 -0.843985 2.435964
H -0.137567 -0.198404 2.556384
C -1.328805 0.133512 0.093824
C -2.255026 -0.907669 -0.088286
C -1.678908 1.488111 -0.043046
C -3.565686 -0.559214 -0.427450
C -3.001342 1.786068 -0.383568
C -3.935720 0.773368 -0.574901
H -4.308468 -1.345869 -0.580167
H -3.304162 2.829021 -0.502516
H -4.964781 1.026034 -0.841740
C -1.849619 -2.361562 0.044856
H -0.825475 -2.382464 0.446844
C -0.654701 2.591054 0.133297
H 0.247177 2.134945 0.569071
C -0.261178 3.180568 -1.221965
H 0.133599 2.404421 -1.894304
H -1.126515 3.647523 -1.719251
H 0.515717 3.951462 -1.100593
C -1.131564 3.673322 1.099259
H -2.005541 4.217612 0.707939
H -1.410990 3.245215 2.073598
H -0.334398 4.413442 1.268339
C -1.818175 -3.040803 -1.324791

H -2.817479 -3.050899 -1.788976
H -1.134546 -2.519826 -2.011593
H -1.477583 -4.083885 -1.233645
C -2.741921 -3.117095 1.027503
H -2.751946 -2.629915 2.014059
H -3.782871 -3.178583 0.672513
H -2.378713 -4.147753 1.160415
C 5.975217 0.429220 -0.318045
C 4.646418 0.350942 -0.204744
H 6.484996 1.383188 -0.165930
H 6.589127 -0.439722 -0.571320
H 4.038316 1.223224 0.058522
H 0.840302 -0.347711 -1.552346
S 3.719170 -1.114307 -0.506448

Coordinates of the optimized structure of **I** for n = 1

E = -1191.14985066
C -0.968308 0.000131 -0.042637
C -1.950602 0.000363 0.970174
C -1.390406 0.000557 2.293770
C -0.040904 0.000445 2.081035
N 0.176662 0.000202 0.702121
H -1.902657 0.000768 3.254526
H 0.791036 0.000512 2.783946
C 1.491095 -0.000081 0.146420
C 2.120066 1.229938 -0.112009
C 2.119542 -1.230368 -0.111958
C 3.411318 1.205634 -0.647193
C 3.410798 -1.206633 -0.647166
C 4.051996 -0.000644 -0.911053
H 3.924769 2.145289 -0.860953
H 3.923833 -2.146520 -0.860914
H 5.062701 -0.000865 -1.326974
C 1.382215 2.533246 0.119075
H 0.643533 2.347621 0.914980
C 1.381191 -2.533379 0.119208
H 0.642318 -2.347311 0.914830
C 0.602327 -2.913460 -1.142820
H -0.080584 -2.098009 -1.426132
H 1.291116 -3.099327 -1.983103
H 0.011599 -3.829090 -0.976487
C 2.283854 -3.670771 0.584525
H 2.986365 -3.989348 -0.202092
H 2.874103 -3.385326 1.468721
H 1.678327 -4.551401 0.848902
C 0.603074 2.913290 -1.142798
H 1.291681 3.098828 -1.983304
H -0.080134 2.097974 -1.425790
H 0.012638 3.829103 -0.976450
C 2.285382 3.670483 0.583791
H 2.875925 3.385070 1.467802
H 2.987645 3.988696 -0.203195

H 1.680202 4.551331 0.848232
C -3.894858 1.131121 -0.837727
C -3.894866 -1.130816 -0.837436
C -4.555269 0.000052 -1.630990
H -2.879327 1.366374 -1.194232
H -4.471378 2.035111 -0.600951
H -2.879328 -1.366161 -1.193871
H -4.471391 -2.034740 -0.600419
H -4.330092 -0.000089 -2.707054
H -5.648074 0.000068 -1.504787
S -3.616084 0.000337 0.600290

Coordinates of the optimized structure of $\mathbf{TS}_{I\rightarrow II}$ for n = 1

E = -1191.12601054
C -1.007934 -0.310549 0.028985
C -1.983999 -0.526002 0.983424
C -1.437030 -0.499223 2.296816
C -0.096113 -0.253019 2.105552
N 0.142057 -0.147826 0.743968
H -1.939118 -0.638051 3.252594
H 0.716517 -0.148385 2.823905
C 1.426730 0.107546 0.179473
C 1.819115 1.440655 -0.031100
C 2.252455 -0.980988 -0.148540
C 3.078855 1.668523 -0.592266
C 3.503518 -0.704305 -0.707726
C 3.913277 0.606806 -0.926939
H 3.413131 2.692544 -0.770885
H 4.168109 -1.527920 -0.976494
H 4.895456 0.803852 -1.363738
C 0.873953 2.583213 0.283220
H 0.178622 2.222586 1.057566
C 1.767191 -2.404104 0.040138
H 0.996226 -2.381268 0.826228
C 1.094652 -2.901215 -1.242080
H 0.261311 -2.243533 -1.528841
H 1.815005 -2.925902 -2.075808
H 0.697704 -3.919479 -1.103831
C 2.864637 -3.358870 0.499176
H 3.626992 -3.517776 -0.279991
H 3.375272 -2.986230 1.400135
H 2.435299 -4.344876 0.733969
C 0.039541 2.927328 -0.953367
H 0.685019 3.280193 -1.773943
H -0.514081 2.045753 -1.308471
H -0.686375 3.724287 -0.724640
C 1.580685 3.815930 0.837626
H 2.210403 3.565885 1.704884
H 2.221362 4.299353 0.083112
H 0.840982 4.565494 1.158286
C -4.260645 0.802460 -0.239184
C -3.144179 -0.786763 -1.434461

C -4.022876 0.438605 -1.709859
H -3.612614 1.590381 0.167404
H -5.299702 0.951885 0.084657
H -1.734004 -0.481523 -1.109057
H -3.362690 -1.732158 -1.950280
H -3.512053 1.230007 -2.285774
H -4.977016 0.226289 -2.220514
S -3.591573 -0.832050 0.334902

Coordinates of the optimized structure of the **II** for n = 1

E = -1191.15558893
C -0.943745 -0.432380 -0.137306
C -1.996879 -0.733508 0.699102
C -1.501518 -0.734373 2.033117
C -0.162040 -0.413166 1.957394
N 0.166927 -0.235412 0.636495
H -2.061291 -0.943404 2.943163
H 0.594057 -0.297540 2.731692
C 1.452582 0.135354 0.145159
C 1.738587 1.501329 -0.025883
C 2.379008 -0.874906 -0.164211
C 2.995705 1.843210 -0.531892
C 3.623555 -0.482987 -0.666102
C 3.929097 0.861534 -0.849119
H 3.247896 2.895543 -0.681167
H 4.364699 -1.244072 -0.920863
H 4.906731 1.148176 -1.244207
C 0.705660 2.566280 0.284693
H -0.090406 2.087619 0.875322
C 2.029357 -2.340824 -0.006665
H 1.102088 -2.395124 0.583606
C 1.740975 -2.969940 -1.370803
H 0.927505 -2.440780 -1.888976
H 2.630987 -2.936580 -2.019640
H 1.442436 -4.023860 -1.258472
C 3.102418 -3.116591 0.753395
H 4.051261 -3.160915 0.195730
H 3.309292 -2.659768 1.732847
H 2.775443 -4.153840 0.923474
C 0.068379 3.087284 -1.004755
H 0.818593 3.571601 -1.650340
H -0.393613 2.271358 -1.579988
H -0.715201 3.827749 -0.780388
C 1.276770 3.705250 1.125795
H 1.740527 3.328231 2.049663
H 2.038814 4.280935 0.577104
H 0.478390 4.408750 1.407635
C -4.401799 0.545879 0.206745
C -3.944374 -0.682914 -1.552242
C -4.057113 0.797953 -1.272455
H -3.910073 1.156195 0.978004
H -5.476724 0.437323 0.402722

H -0.912931 -0.346754 -1.221991
H -3.451404 -1.188972 -2.387095
H -3.126572 1.404445 -1.340809
H -4.838042 1.339615 -1.836321
S -3.676795 -1.143310 0.075627

Coordinates of the optimized structure of **TS**$_{I\rightarrow III}$ for n = 1

E = -1191.10898170
C 1.077064 0.000130 0.024605
C 2.094114 0.000068 0.974823
C 1.554664 -0.000024 2.293118
C 0.190766 -0.000110 2.113858
N -0.073129 -0.000042 0.754735
H 2.085056 -0.000075 3.243130
H -0.620680 -0.000217 2.839519
C -1.386795 0.000013 0.197696
C -2.007009 -1.232003 -0.069391
C -2.006831 1.232092 -0.069478
C -3.292190 -1.206730 -0.618555
C -3.291995 1.206973 -0.618693
C -3.929164 0.000160 -0.888636
H -3.802386 -2.146243 -0.840466
H -3.802031 2.146551 -0.840714
H -4.934474 0.000216 -1.317075
C -1.272616 -2.536395 0.166882
H -0.502166 -2.341118 0.929237
C -1.272282 2.536401 0.166748
H -0.501477 2.340935 0.928687
C -0.549873 2.967267 -1.112577
H 0.139926 2.184490 -1.460384
H -1.272596 3.167156 -1.920123
H 0.031138 3.887393 -0.940429
C -2.172771 3.646768 0.698605
H -2.913758 3.971637 -0.048898
H -2.719115 3.328417 1.599374
H -1.571048 4.530814 0.959490
C -0.549653 -2.967048 -1.112208
H -1.272051 -3.166743 -1.920092
H 0.140350 -2.184277 -1.459605
H 0.031217 -3.887248 -0.939974
C -2.173365 -3.646875 0.698054
H -2.720293 -3.328680 1.598525
H -2.913857 -3.971708 -0.049954
H -1.571744 -4.530913 0.959198
C 3.677930 1.133160 -1.010316
C 3.677942 -1.133475 -1.010036
C 3.068797 -0.000285 -1.819595
H 4.714924 1.476116 -1.217684
H 3.050718 2.009651 -0.759974
H 4.714945 -1.476451 -1.217324
H 3.050768 -2.009924 -0.759456
H 3.262793 -0.000436 -2.902695

H 1.613227 -0.000108 -1.156348
S 3.760883 0.000033 0.461341

Coordinates of the optimized structure of the **III** for n = 1

E = -1191.11992466
C 1.012124 -0.240724 0.001767
C 2.050934 -0.446870 0.894202
C 1.508404 -0.523196 2.209566
C 0.150801 -0.343985 2.070081
N -0.131650 -0.175915 0.731252
H 2.050457 -0.683098 3.138924
H -0.645999 -0.317325 2.809798
C -1.420308 0.099026 0.182133
C -2.270424 -0.976493 -0.125457
C -1.771915 1.439620 -0.049691
C -3.514950 -0.675445 -0.685326
C -3.025144 1.687877 -0.617362
C -3.887443 0.642561 -0.930319
H -4.201488 -1.485303 -0.939921
H -3.331078 2.716969 -0.816750
H -4.863342 0.857499 -1.372371
C -1.820874 -2.409943 0.075608
H -1.022300 -2.399790 0.833692
C -0.813761 2.572170 0.264571
H -0.066137 2.180766 0.972249
C -0.064894 3.017261 -0.994884
H 0.529120 2.201810 -1.434200
H -0.769005 3.378195 -1.761771
H 0.626700 3.840912 -0.757524
C -1.506758 3.751524 0.942877
H -2.207576 4.263389 0.264728
H -2.070215 3.432490 1.832719
H -0.761894 4.497393 1.259498
C -1.212989 -2.955104 -1.219397
H -1.963718 -2.975577 -2.025565
H -0.371424 -2.333938 -1.559346
H -0.841318 -3.981414 -1.073256
C -2.931327 -3.318693 0.593732
H -3.393173 -2.913148 1.506559
H -3.728252 -3.463270 -0.152590
H -2.526971 -4.314774 0.829567
C 4.005820 0.944968 -0.647440
C 3.687662 -1.202590 -1.270377
C 3.415590 0.186053 -1.802802
H 5.115410 1.093344 -0.558463
H 3.519716 1.856720 -0.243374
H 4.668953 -1.719343 -1.443749
H 2.898640 -1.982241 -1.297708
H 3.822890 0.426571 -2.796063
H 1.113439 -0.079582 -1.077140
S 3.737302 -0.522121 0.462152

Coordinates of the optimized structure of **TS**$_{III\rightarrow IV}$ for n = 1

E = -1191.11875762
C 0.989995 -0.369237 -0.022435
C 2.026017 -0.636326 0.859764
C 1.489663 -0.676215 2.180748
C 0.144911 -0.415912 2.055267
N -0.141800 -0.232526 0.719112
H 2.030437 -0.867555 3.104946
H -0.641983 -0.346226 2.802614
C -1.411458 0.132255 0.180458
C -2.329756 -0.880666 -0.142621
C -1.676511 1.496635 -0.029839
C -3.550833 -0.492164 -0.700557
C -2.909495 1.833205 -0.596354
C -3.836261 0.850385 -0.927748
H -4.287861 -1.252470 -0.967234
H -3.148448 2.882785 -0.779842
H -4.794721 1.134509 -1.368964
C -1.979314 -2.343176 0.044950
H -1.158623 -2.391825 0.777319
C -0.652098 2.561831 0.311766
H 0.062869 2.112180 1.018541
C 0.137310 2.985255 -0.930440
H 0.714385 2.152220 -1.360446
H -0.537008 3.378938 -1.708023
H 0.854072 3.780667 -0.672026
C -1.279478 3.768059 1.007030
H -1.937033 4.337414 0.331307
H -1.874400 3.467007 1.882635
H -0.493571 4.458541 1.349010
C -1.454860 -2.931152 -1.267223
H -2.228825 -2.894396 -2.050588
H -0.579757 -2.373770 -1.632377
H -1.156511 -3.982548 -1.131694
C -3.137718 -3.166644 0.600158
H -3.534418 -2.730261 1.529215
H -3.969366 -3.245324 -0.117609
H -2.803667 -4.192018 0.820323
C 4.110127 0.867656 -0.545627
C 3.595943 -1.151786 -1.389632
C 3.417869 0.310520 -1.724316
H 5.223523 0.773018 -0.427965
H 3.804439 1.828455 -0.085209
H 4.525903 -1.711397 -1.672442
H 2.743632 -1.849841 -1.505211
H 3.685895 0.694106 -2.718366
H 1.057223 -0.189053 -1.095580
S 3.704506 -0.737538 0.428987

Coordinates of the optimized structure of the sulfide for n = 1

E = -1191.24247295
C 1.057557 -0.286515 0.083467

C 2.064996 -0.534760 0.998416
C 1.456424 -0.609278 2.285481
C 0.105088 -0.401738 2.106240
N -0.129154 -0.214086 0.771216
H 1.960053 -0.802523 3.230317
H -0.714780 -0.378010 2.820586
C -1.389838 0.071942 0.175847
C -2.206553 -0.996253 -0.233636
C -1.762940 1.416314 0.000151
C -3.428414 -0.687047 -0.838285
C -2.992806 1.675578 -0.611442
C -3.817778 0.635096 -1.026389
H -4.084379 -1.494952 -1.170765
H -3.309815 2.709572 -0.765848
H -4.775928 0.857451 -1.502484
C -1.757043 -2.434297 -0.072664
H -0.893911 -2.431745 0.610069
C -0.846624 2.547580 0.421849
H -0.067778 2.113782 1.067318
C -0.145684 3.154401 -0.795147
H 0.437940 2.396888 -1.338796
H -0.875071 3.590054 -1.497063
H 0.546201 3.953409 -0.485606
C -1.575679 3.613641 1.236159
H -2.331975 4.144908 0.636862
H -2.084480 3.174297 2.107288
H -0.862299 4.367577 1.602841
C -1.277693 -2.995071 -1.412740
H -2.094976 -3.011227 -2.151802
H -0.460905 -2.385689 -1.827294
H -0.906864 -4.025215 -1.294017
C -2.834647 -3.317413 0.551205
H -3.180543 -2.908749 1.512524
H -3.712957 -3.422285 -0.105288
H -2.441292 -4.329336 0.733191
C 3.534825 1.511855 -1.514218
C 3.851463 -0.958584 -1.067974
C 3.701057 0.258364 -1.932952
H 3.490187 1.741698 -0.444935
H 3.440382 2.337194 -2.223951
H 4.833165 -1.426371 -1.255859
H 3.099112 -1.720086 -1.339001
H 3.741541 0.050865 -3.010626
H 1.090154 -0.127540 -0.990263
S 3.790565 -0.716141 0.722743

Coordinates of the optimized structure of **I** for n = 2

E = -1230.45749380
C 0.767806 0.322988 -0.014166
C 1.703949 0.614342 0.991001
C 1.127080 0.646290 2.302926
C -0.195358 0.359618 2.091806

N -0.378558 0.179339 0.725547
H 1.606113 0.850140 3.259606
H -1.027072 0.281505 2.790878
C -1.654430 -0.120678 0.163853
C -2.011437 -1.466331 -0.030255
C -2.520047 0.936241 -0.167372
C -3.270197 -1.739957 -0.573407
C -3.769634 0.616109 -0.706284
C -4.142830 -0.709080 -0.906365
H -3.573964 -2.775944 -0.737674
H -4.462416 1.416154 -0.975146
H -5.124702 -0.940893 -1.326786
C -1.024697 -2.574216 0.278447
H -0.344304 -2.188521 1.054191
C -2.069100 2.374452 -0.010817
H -1.323987 2.391815 0.800169
C -1.358332 2.831001 -1.287531
H -0.516122 2.159811 -1.513857
H -2.052734 2.820786 -2.143614
H -0.970918 3.856456 -1.173482
C -3.194720 3.327807 0.375123
H -3.930757 3.448623 -0.435785
H -3.732927 2.980106 1.270231
H -2.787478 4.327904 0.589585
C -0.177595 -2.867834 -0.962412
H -0.807152 -3.250379 -1.782375
H 0.318129 -1.946015 -1.303501
H 0.591959 -3.625898 -0.741427
C -1.679770 -3.838785 0.823325
H -2.317895 -3.622331 1.693689
H -2.301703 -4.341720 0.065682
H -0.909856 -4.560976 1.136426
S 3.385165 0.862663 0.665430
C 3.364102 1.275927 -1.123462
H 2.277331 1.255882 -1.338941
H 3.792994 2.277782 -1.251694
C 4.092425 0.155645 -1.851856
H 3.740781 0.103623 -2.893671
H 5.179809 0.339288 -1.878334
C 3.787995 -1.125386 -1.082865
H 2.715941 -1.363230 -1.175905
H 4.371806 -1.986857 -1.441304
C 4.100844 -0.811261 0.366455
H 3.638445 -1.477791 1.106699
H 5.181615 -0.728656 0.563752

Coordinates of the optimized structure of **TS**$_{I\rightarrow II}$ for n = 2

E = -1230.43563018
C 0.742458 -0.498869 -0.041418
C 1.700889 -0.848932 0.886922
C 1.180376 -0.832095 2.208848
C -0.129933 -0.431277 2.055501

N -0.373678 -0.238309 0.707948
H 1.676703 -1.066206 3.149153
H -0.915995 -0.277847 2.794395
C -1.637634 0.161160 0.183710
C -2.573736 -0.829303 -0.161303
C -1.905644 1.532046 0.031468
C -3.805565 -0.412514 -0.673833
C -3.150785 1.901949 -0.485540
C -4.092548 0.939513 -0.833688
H -4.553112 -1.157735 -0.953392
H -3.386876 2.959942 -0.619059
H -5.061690 1.246247 -1.234979
C -2.223646 -2.298621 -0.040018
H -1.438300 -2.381119 0.727603
C -0.848536 2.568572 0.354109
H -0.128294 2.092842 1.037575
C -0.088021 2.946958 -0.918650
H 0.345502 2.053849 -1.391807
H -0.759512 3.427233 -1.648754
H 0.729043 3.651225 -0.693146
C -1.409227 3.799924 1.058770
H -2.077639 4.382279 0.404892
H -1.976426 3.524889 1.960882
H -0.590794 4.470986 1.362139
C -1.626505 -2.802515 -1.356401
H -2.362086 -2.724403 -2.173471
H -0.740116 -2.213468 -1.633343
H -1.324901 -3.858396 -1.268107
C -3.397875 -3.163695 0.406334
H -3.853228 -2.782429 1.333010
H -4.186959 -3.217594 -0.360534
H -3.059826 -4.194729 0.591840
S 3.271693 -1.283583 0.189322
C 4.350846 0.153458 0.632995
H 3.731878 0.810930 1.259962
H 5.207599 -0.215077 1.213853
C 4.715590 0.779556 -0.705349
H 5.038766 1.823841 -0.559181
H 5.561023 0.232674 -1.154368
C 3.480827 0.655436 -1.599625
H 2.722843 1.391208 -1.269067
H 3.714769 0.885140 -2.650277
C 2.907630 -0.746632 -1.475308
H 1.512319 -0.611421 -1.202492
H 3.310180 -1.492097 -2.173252

Coordinates of the optimized structure of the **II** for n = 2

E = -1230.46940683
C -1.746901 -0.715451 0.010740
C -1.507769 -0.713610 1.407861
C -0.174259 -0.409255 1.586172
N 0.397565 -0.238653 0.350577

H -2.254623 -0.922276 2.173170
H 0.424682 -0.295970 2.487468
C 1.754153 0.122790 0.108812
C 2.080551 1.487439 0.014624
C 2.713304 -0.893246 -0.039507
C 3.412324 1.821536 -0.245565
C 4.032946 -0.509480 -0.296768
C 4.379660 0.833378 -0.400337
H 3.697198 2.873024 -0.328937
H 4.801497 -1.276540 -0.419646
H 5.416085 1.113796 -0.603425
C 1.020659 2.561918 0.153951
H 0.110373 2.076025 0.537063
C 2.336057 -2.357917 0.049391
H 1.280386 -2.406886 0.355787
C 2.448376 -3.031812 -1.318363
H 1.817884 -2.524788 -2.064047
H 3.485793 -3.016311 -1.689442
H 2.127019 -4.083386 -1.260830
C 3.155456 -3.092619 1.108628
H 4.226859 -3.116270 0.853453
H 3.055774 -2.612657 2.093716
H 2.815606 -4.135565 1.202280
C 0.679745 3.162175 -1.210806
H 1.555432 3.660365 -1.657142
H 0.342108 2.385618 -1.913322
H -0.123716 3.909632 -1.117928
C 1.419393 3.641470 1.157437
H 1.661290 3.205539 2.138267
H 2.296720 4.214773 0.818419
H 0.594965 4.357531 1.297079
S -3.383326 -1.152176 -0.727640
C -5.058511 0.184454 0.836328
H -6.151180 0.299605 0.699950
H -4.865886 0.402394 1.905192
C -4.285690 1.213656 -0.018076
H -4.898849 2.095682 -0.260338
H -3.406025 1.573045 0.538702
C -3.797111 0.536964 -1.294165
H -4.593819 0.396338 -2.039324
H -2.924750 1.021427 -1.755919
C -0.551749 -0.431232 -0.620482
H -0.290719 -0.347740 -1.673535
C -4.617943 -1.178332 0.371290
H -5.005975 -2.137979 0.704499

Coordinates of the optimized structure of the sulfide for n = 2

E = -1230.52367161
C -0.438457 -0.660966 -0.701790
C -1.636820 -1.056202 -0.131326
C -1.478620 -0.965570 1.283470
C -0.199099 -0.514627 1.518321

N 0.423041 -0.331899 0.305854
H -2.237992 -1.203733 2.025077
H 0.332421 -0.303175 2.443620
C 1.738758 0.182762 0.123933
C 1.903561 1.574200 0.003351
C 2.819033 -0.713302 0.062940
C 3.198379 2.060342 -0.196496
C 4.095463 -0.179098 -0.137831
C 4.283889 1.192698 -0.267733
H 3.360040 3.135879 -0.299133
H 4.956025 -0.849881 -0.194882
H 5.289052 1.591233 -0.425426
C 0.714614 2.513648 0.047238
H -0.142702 1.938275 0.429408
C 2.611193 -2.209877 0.174223
H 1.568486 -2.373429 0.485701
C 2.793391 -2.881148 -1.187682
H 2.103901 -2.459570 -1.934167
H 3.820210 -2.746825 -1.564396
H 2.597333 -3.962368 -1.117781
C 3.510901 -2.838626 1.235940
H 4.577054 -2.759012 0.970591
H 3.371499 -2.356231 2.215031
H 3.280139 -3.909149 1.347931
C 0.354268 2.990992 -1.360597
H 1.178734 3.570022 -1.806921
H 0.141243 2.141693 -2.026485
H -0.538338 3.635730 -1.336510
C 0.935468 3.688300 0.997034
H 1.197110 3.342077 2.008119
H 1.741998 4.353056 0.649486
H 0.021071 4.296813 1.071179
S -3.072842 -1.572108 -0.999795
C -4.842661 0.626572 0.992574
H -0.140720 -0.592109 -1.745463
H -4.896513 1.391047 1.778448
C -3.881390 0.036611 -1.336594
H -4.883041 -0.236429 -1.707413
H -3.346523 0.521461 -2.167722
C -3.975129 1.001235 -0.168966
H -2.956079 1.212682 0.205202
H -4.333712 1.969917 -0.565008
C -5.539721 -0.498827 1.156489
H -6.153523 -0.651288 2.048254
H -5.505131 -1.314371 0.429998

Coordinates of the optimized structure of **TS**$_{I \rightarrow IV}$ for n = 2

E = -1230.42223987
C -0.922235 -0.230170 0.150035
C -1.884223 -0.250563 1.165247
C -1.277567 -0.099455 2.449062
C 0.064978 0.025218 2.188413

N 0.257054 -0.034313 0.818373
H -1.755399 -0.084754 3.426673
H 0.912015 0.119829 2.866127
C 1.538202 0.011371 0.195210
C 2.112319 1.264857 -0.080837
C 2.181825 -1.196230 -0.125228
C 3.362743 1.286754 -0.705122
C 3.428889 -1.124267 -0.753035
C 4.014392 0.104134 -1.040226
H 3.833536 2.244404 -0.936454
H 3.951846 -2.045451 -1.019180
H 4.990928 0.140795 -1.529427
C 1.362564 2.546114 0.227510
H 0.655562 2.320524 1.040900
C 1.522249 -2.530666 0.158108
H 0.751461 -2.354165 0.924226
C 0.808070 -3.042908 -1.094444
H 0.060367 -2.311872 -1.435866
H 1.525392 -3.214760 -1.913435
H 0.293694 -3.995110 -0.887320
C 2.494187 -3.568363 0.712632
H 3.248066 -3.869018 -0.032184
H 3.027499 -3.189654 1.597718
H 1.950361 -4.479408 1.006359
C 0.535942 2.975355 -0.987156
H 1.190297 3.195951 -1.845990
H -0.166824 2.185941 -1.289746
H -0.045969 3.883372 -0.761337
C 2.269628 3.674127 0.708421
H 2.892972 3.357987 1.558598
H 2.941165 4.032752 -0.087742
H 1.665144 4.535835 1.030635
S -3.557744 -0.155812 0.756666
C -2.745683 0.836486 -1.560904
H -1.503632 0.295402 -0.910165
H -2.376323 1.541356 -2.314061
C -3.692828 -1.264094 -0.706136
H -4.716728 -1.663064 -0.690561
H -2.986862 -2.078352 -0.495563
C -3.353851 -0.473340 -1.995819
H -2.656112 -1.059259 -2.617471
H -4.291709 -0.364486 -2.573967
C -3.559984 1.369019 -0.474808
H -3.167898 2.240369 0.072427
H -4.656007 1.467753 -0.603998

4. Literature

1. Sergeev, A.G.; Schulz, T.; Torborg, C.; Spannenberg, A.; Neumann, H.; Beller, M. Palladium-catalyzed hydroxylation of aryl halides under ambient conditions. *Angew. Chem. Int. Ed. Engl.* **2009**, *48*, 7595–7599, doi:10.1002/anie.200902148.
2. Fürstner, A.; Alcarazo, M.; Radkowski, K.; Lehmann, C.W. Carbenes stabilized by ylides: Pushing the limits. *Angew. Chem. Int. Ed. Engl.* **2008**, *47*, 8302–8306, doi:10.1002/anie.200803200.
3. Hartke, K.; Teuber, D.; Gerber, H.-D. Indole- and pyrrole-sulfonium ylides. *Tetrahedron* **1988**, *44*, 3261–3270, doi:10.1016/S0040-4020(01)85959-7.
4. Kobayashi, J.; Nakafuji, S.-Y.; Yatabe, A.; Kawashima, T. A novel ylide-stabilized carbene; formation and electron donating ability of an amino(sulfur-ylide)carbene. *Chem. Commun.* **2008**, 6233–6235, doi:10.1039/b813454e.
5. Alvarez, A.; Guzman, A.; Ruiz, A.; Velarde, E.; Muchowski, J.M. Synthesis of 3-arylpyrroles and 3-pyrrolylacetylenes by palladium-catalyzed coupling reactions. *J. Org. Chem.* **1992**, *57*, 1653–1656, doi:10.1021/jo00032a011.
6. Asay, M.; Donnadieu, B.; Baceiredo, A.; Soleilhavoup, M.; Bertrand, G. Cyclic (amino)bis(ylide)carbene as an anionic bidentate ligand for transition-metal complexes. *Inorg. Chem.* **2008**, *47*, 3949–3951, doi:10.1021/ic800459p.
7. Thorn, A.; Dittrich, B.; Sheldrick, G.M. Enhanced rigid-bond restraints. *Acta Crystallogr., Sect. A: Fundam. Crystallogr.* **2012**, *68*, 448–451, doi:10.1107/S0108767312014535.
8. Sheldrick, G.M. SHELXT - integrated space-group and crystal-structure determination. *Acta Crystallogr., Sect. A: Fundam. Crystallogr.* **2015**, *71*, 3–8, doi:10.1107/S2053273314026370.
9. Sheldrick, G.M. A short history of SHELX. *Acta Crystallogr., Sect. A: Fundam. Crystallogr.* **2008**, *64*, 112–122, doi:10.1107/S0108767307043930.
10. Pennington, W.T. DIAMOND – Visual Crystal Structure Information System. *J. Appl. Crystallogr.* **1999**, *32*, 1028–1029, doi:10.1107/S0021889899011486.
11. The GIMP team. *GIMP 2.10*, 1995-2019.
12. Spek, A.L. Structure validation in chemical crystallography. *Acta Crystallogr., Sect. D: Biol. Crystallogr.* **2009**, *65*, 148–155, doi:10.1107/S090744490804362X.
13. *GaussView, Version 3.0;* Gaussian, Inc.: Pittburgh, PA, United States of America, 2000. (Available online: https://gaussview.software.informer.com/3.0/)
14. Dennington, R.; Keith, T.A.; Millam, J.M. *GaussView, Version 6.0;* Semichem Inc.: Shawnee Mission, United States of America 2016. (Available online: https://gaussian.com/gaussview6/)
15. M. J. Frisch; G. W. Trucks; H. B. Schlegel; G. E. Scuseria; M. A. Robb; J. R. Cheeseman; G. Scalmani; V. Barone; B. Mennucci; G. A. Petersson; et al. *Gaussian 09; Revision, E.01;* Gaussian, Inc.: Wallingford, CT, United States of America, 2009. (Available online: https://gaussian.com/glossary/g09/).
16. M. J. Frisch; G. W. Trucks; H. B. Schlegel; G. E. Scuseria; M. A. Robb; J. R. Cheeseman; G. Scalmani; V. Barone; G. A. Petersson; H. Nakatsuji; et al. *Gaussian 16, Revision, B.01;* Gaussian, Inc.: Wallingford, CT, United States of America, 2016. (Available online: https://gaussian.com/gaussian16/)
17. M. J. Frisch; G. W. Trucks; H. B. Schlegel; G. E. Scuseria; M. A. Robb; J. R. Cheeseman; G. Scalmani; V. Barone; G. A. Petersson; H. Nakatsuji; et al. *Gaussian 16, Revision, C.01;* Gaussian,

Inc.: Wallingford, CT, United States of America, 2016. (Available online: https://gaussian.com/gaussian16/)

18. Hohenberg, P.; Kohn, W. Inhomogeneous Electron Gas. *Phys. Rev.* **1964**, *136*, B864-B871, doi:10.1103/PhysRev.136.B864.
19. Kohn, W.; Sham, L.J. Self-Consistent Equations Including Exchange and Correlation Effects. *Phys. Rev.* **1965**, *140*, A1133-A1138, doi:10.1103/PhysRev.140.A1133.
20. Adamo, C.; Barone, V. Toward reliable density functional methods without adjustable parameters: The PBE0 model. *J. Chem. Phys.* **1999**, *110*, 6158–6170, doi:10.1063/1.478522.
21. Weigend, F.; Ahlrichs, R. Balanced basis sets of split valence, triple zeta valence and quadruple zeta valence quality for H to Rn: Design and assessment of accuracy. *Phys. Chem. Chem. Phys.* **2005**, *7*, 3297–3305, doi:10.1039/B508541A.
22. Grimme, S.; Antony, J.; Ehrlich, S.; Krieg, H. A consistent and accurate ab initio parametrization of density functional dispersion correction (DFT-D) for the 94 elements H-Pu. *The Journal of Chemical Physics* **2010**, *132*, 154104, doi:10.1063/1.3382344.
23. Grimme, S.; Antony, J.; Ehrlich, S.; Krieg, H. A consistent and accurate ab initio parametrization of density functional dispersion correction (DFT-D) for the 94 elements H-Pu. *J. Chem. Phys.* **2010**, *132*, 154104, doi:10.1063/1.3382344.
24. Smith, D.G.A.; Burns, L.A.; Patkowski, K.; Sherrill, C.D. Revised Damping Parameters for the D3 Dispersion Correction to Density Functional Theory. *J. Phys. Chem. Lett.* **2016**, *7*, 2197–2203, doi:10.1021/acs.jpclett.6b00780.
25. Deglmann, P.; Furche, F. Efficient characterization of stationary points on potential energy surfaces. *J. Am. Chem. Soc.* **2002**, *117*, 9535–9538, doi:10.1063/1.1523393.
26. Tomasi, J.; Mennucci, B.; Cammi, R. Quantum mechanical continuum solvation models. *Chem. Rev.* **2005**, *105*, 2999–3093, doi:10.1021/cr9904009.
